# Two‐Dimensional Quaternary Alloys of (RuMoVNb)Se_2_ for Superior Hydrogen Evolution Reaction Catalysis

**DOI:** 10.1002/advs.76600

**Published:** 2026-07-24

**Authors:** Junaid Ihsan, Ju Yeon Kim, Irtiqa Mishal, Jun Hyeok Choi, Jeong Eun An, Youn Jun Choi, Doyeon Kim, In Hye Kwak, Gianvito Vilé, Ik Seon Kwon, Hong Seok Kang, Jeunghee Park

**Affiliations:** ^1^ Department of Advanced Materials Chemistry Korea University Sejong Republic of Korea; ^2^ Korea Research Institute Standard and Science Daejeon Republic of Korea; ^3^ Research Center for Materials Analysis Korea Basic Science Institute Daejeon Republic of Korea; ^4^ Department of Chemistry, Materials, and Chemical Engineering ‘‘Giulio Natta’’ Politecnico di Milano Milano Italy; ^5^ Department of Energy Science & Engineering Kunsan National University Gunsan‐si Jeonbuk State Republic of Korea; ^6^ Department of Nano and Advanced Materials Jeonju University Chonju Chonbuk Republic of Korea

**Keywords:** atomic mixing, density functional theory calculation, hydrogen evolution reaction, (RuMoVNb)Se_2_ quaternary alloys, transition metal dichalcogenide

## Abstract

Alloying 2D transition metal dichalcogenides offers a promising strategy to broaden their applications. Herein, (RuMoVNb)Se_2_ quaternary alloy nanosheets (*x*_Ru_ = 0–1) were synthesized via colloidal reactions. Incorporation of Ru into the (MoVNb)Se_2_ maintained exclusively 2D layered structure over wide composition range, accompanied by a phase evolution from the hexagonal 2H to 1T. The quaternary alloying results in more metallic electronic structures than the ternary alloying. First‐principles calculations supported the homogenous atomic mixing of quaternary alloys and the favored 2H → 1T phase transition at higher *x*_Ru_. Compared to (MoVNb)Se_2_, the quaternary alloys exhibited significantly enhanced electrocatalytic activity toward the hydrogen evolution reaction (HER) across alkaline, acidic, and neutral electrolytes. Gibbs free energy calculations show that Ru atoms optimize hydrogen adsorption, directly correlating with the improved HER performance. In situ X‐ray absorption fine structure data (in 1 M KOH) reveal electrolyte adsorption on Ru as key to superior HER catalysis.

## Graphical abstract

Composition‐tuned (RuMoVNb)Se_2_ quaternary alloy nanosheets are synthesized via colloidal reactions. Incorporation of Ru into (MoVNb)Se_2_ ternary alloys results in more metallic electronic structures and significantly enhances their electrocatalytic activity for the hydrogen evolution reaction (HER) under alkaline, acidic, and neutral conditions. First‐principles calculations supported the electronic structure of quaternary alloys and their improved HER performance.

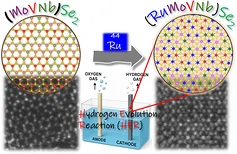

## 1 Introduction

The substantial expansion of fossil fuel consumption has resulted in significant environmental and energy challenges in recent decades. Consequently, the development of sustainable energy sources with reduced carbon emissions has become an important trend. Hydrogen energy is considered a vital contributor in this context, as it offers zero carbon emissions and high energy efficiency as an alternative to fossil fuels. Among the various technologies, green hydrogen production via electrocatalytic water splitting is regarded as the most promising clean energy approach, due to its production of high‐purity hydrogen and cost‐effectiveness. To achieve a robust hydrogen economy, Pt‐based electrodes have been commercially utilized for water splitting in recent decades. However, their widespread use is limited by high cost, as well as issues of stability and environmental concerns. As a result, many alternative catalysts have been explored to achieve efficient electrocatalysis.

Among them, 2D layered transition metal dichalcogenides (TMDs) have emerged as highly reliable candidates. In TMDs, the transition metal (TM) atomic sublayer is sandwiched between two chalcogen (X) atomic sublayers, with TMs ranging from group V to group VII elements. TMDs exhibit diverse structural polymorphs, including hexagonal (2H) and octahedrally coordinated trigonal (1T) phases. These materials have been widely studied as catalysts for the hydrogen evolution reaction (HER) [1, 2, 3, 4, 5, 6]. Nevertheless, pristine TMDs face intrinsic drawbacks for HER electrocatalysis, as sluggish reaction dynamics are caused by low conductivity and poor adsorption capability. Overcoming these limitations is challenging and has prompted the development of several strategies, such as creating chalcogen vacancies, doping, metal or molecular ion intercalation, and alloying. In particular, various alloy systems have demonstrated enhanced electrocatalytic activities [7, 8, 9, 10, 11, 12, 13, 14, 15, 16, 17, 18, 19, 20, 21, 22, 23, 24]. Our group reported that (MoVNb)Se_2_ ternary alloy nanosheets exhibit higher HER activity than their constituent binary alloys [21].

RuX_2_ belongs to the family of TMDs in which the pyrite cubic (C) phase is thermodynamically stable. Both amorphous and crystalline phases have been widely used as excellent electrocatalysts for the HER in acidic and alkaline electrolytes [25, 26, 27, 28, 29, 30, 31, 32, 33, 34, 35, 36, 37, 38, 39, 40]. Zhao et al. reported the synthesis of metastable 2H (major)/1T phase RuSe_2_ nanosheets via colloidal reaction at 200°C, which exhibited superior HER performance compared to the C phase obtained upon annealing at 600°C [28]. Zhan et al. demonstrated that the 1T phase enhances the HER catalytic activity of RuSe_2_ when coexisting with the C phase at 250°C–500°C [31]. More recently, Kim et al. achieved colloidal synthesis of 1T phase Ru(S_x_Se_1‐x_)_2_ alloy nanotubes at 280°C, reporting their enhanced catalytic performance for the oxygen reduction reaction [41]. They proposed that a smaller tube diameter stabilizes the 1T phase. Prior to these experimental findings, Ersan et al. predicted through DFT calculations that the 1T phase could be stabilized via a Peierls distortion (1T′ phase) [42]. The existence of 2H or 1T phase RuSe_2_ raises the natural question of whether alloying with 2D layered TMDs is feasible.

In this study, (RuMoVNb)Se_2_ quaternary alloy nanosheets were synthesized via colloidal reaction. By starting from (MoVNb)Se_2_, the Ru composition was systematically varied over the full range (*x*_Ru_ = 0.05–1), leading to continuous phase evolution. Since RuSe_2_ can adopt either 2H/1T layered phase or a cubic phase, phase transitions were examined by annealing up to 600°C. Atomic‐resolution scanning transmission electron microscopy (STEM) was used to probe the composition‐dependent crystal structures. The electronic structures were comprehensively analyzed using X‐ray photoelectron spectroscopy (XPS) and X‐ray absorption fine structure (XAFS) measurements. Furthermore, first‐principles calculations were carried out for both the 2H and 1T phases, considering pristine and Se‐vacancy models, to support the experimental findings. The electrocatalytic HER activity was evaluated in electrolytes covering the entire pH range, and the calculated Gibbs free energy of H adsorption (ΔG_H*_) provided theoretical insights into the enhanced catalytic performance of the quaternary alloys.

## 2 Results and Discussions

Figure 1a presents a schematic illustration of the colloidal synthesis of (RuMoVNb)Se_2_ nanosheets at 260°C, using RuCl_3_, MoCl_5_, NbCl_5_, VCl_3_, and (C_6_H_5_CH_2_)_2_Se_2_ (dibenzyl diselenide, DBDS) as precursors in oleylamine. Starting from the (MoVNb)Se_2_ ternary alloy, samples were synthesized using various mole fractions of the Ru precursor (*x*_RuCl3_ = 0, 0.05, 0.1, 0.14, 0.25, 0.4, 0.7, and 1), while maintaining equal molar ratios of the other metal precursors. One sample was prepared with *x*_RuCl3_ = 0.4, *x*_MoCl5_ = 0.4, and *x*_NbCl5_ = *x*_VCl3_ = 0.1. The products were subjected to annealing at either 400°C or 600°C. The composition of the products matched that of the starting precursors (see Table S1 and Figure S1). The nine samples (Samples **1**–**9**) were ordered according to increasing Ru content, and their compositions are summarized in the table in the bottom panel. The as‐synthesized, 400°C‐annealed, and 600°C‐annealed samples are denoted as “UA,” “400A,” and “600A,” respectively. Unless otherwise specified, 600A samples are mainly discussed throughout the paper, without the “600A” notation.

**FIGURE 1:**
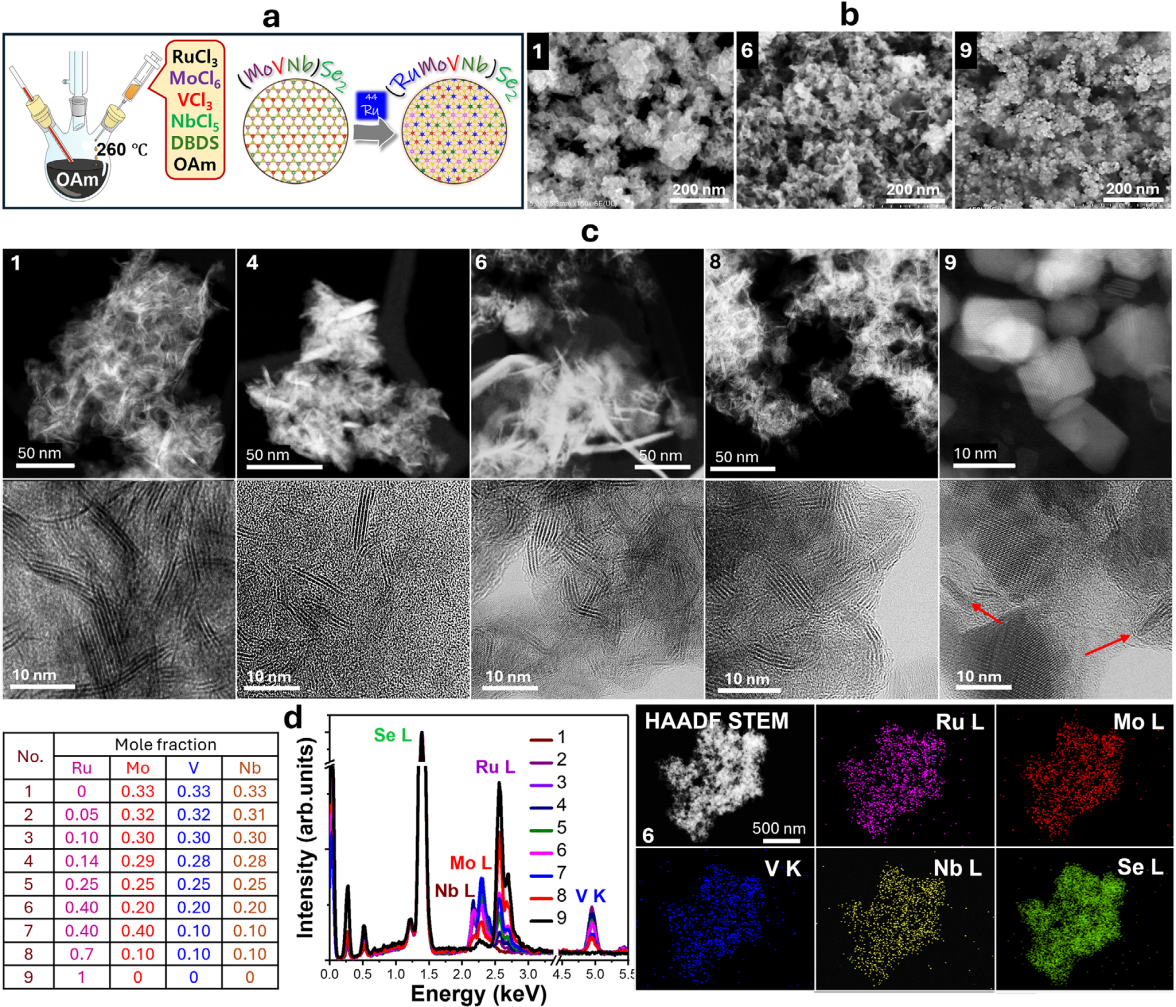
(a) Schematic diagram for the colloidal synthesis of (RuMoVNb)Se_2_ quaternary alloy nanosheets. The composition of samples **1**–**9** is summarized in the table (bottom). (b) SEM, (c) HAADF STEM, and HRTEM images of samples with *x*_Ru_ = 0 (sample **1**), 0.25 (**5**), 0.4 (**6**), 0.7 (**8**), and 1 (**9**). In sample **9,** nanosheets are indicated by arrows. (d) EDX spectra showing composition tuning in samples **1**–**9**. HAADF STEM image and EDX elemental mapping of Ru (L shell), Mo (L shell), V (K shell), Nb (L shell), and Se (L shell) for sample **6**.

Figure 1b,c shows scanning electron microscopy (SEM) and high‐angle annular dark‐field (HAADF) STEM/high‐resolution TEM (HRTEM) images of the 600A samples, respectively. Sample **1** (*x*_Ru_ = 0) consists of avg. 3 layered nanosheets (see more TEM images with statistics in Figure S1), which aggregate into flower‐like nanoparticles (nanoflowers) with sizes ranging from 50 to 100 nm. Upon Ru incorporation (samples **4** and **6**), the lateral size of the nanosheets and nanoflowers decreases, while the number of layers remains the same. At *x*_Ru_ = 0.7 (sample **8**), the nanosheet size is less than 10 nm. The interlayer distance of the nanosheets is approximately 6 Å. In sample **9** (RuSe_2_), the morphology changes dramatically to cube‐shaped nanocrystals about 10 nm in size (see the statistics in Figure S1). This nanocube morphology is characteristic of the cubic phase. However, nanosheet morphology is still observed, as indicated by arrows. The morphology evolution of nanosheets with increasing *x*_Ru_ is probably related to the phase change. Figure S1 presents SEM and HRTEM images of all samples, including UA and 400A. Annealing does not alter the nanosheet morphology of alloy samples **1** through **8**. Only RuSe_2_ exhibits a morphology change, transitioning from nanosheets (UA and 400A) to nanocubes (600A). The RuSe_2_ nanosheets have a size of approximately 5 nm and consist of about 2 layers.

Energy‐dispersive X‐ray spectroscopy (EDX) data confirmed successful composition tuning (Figure 1d). In the normalized EDX spectra, the intensity of Ru peaks increases with the sample number, demonstrating effective control of Ru content throughout the series. EDX elemental mapping and the corresponding HAADF STEM images of sample **6** reveal a homogeneous distribution of Ru, Mo, V, Nb, and Se. The 600A and 400A samples with *x*_Ru_ = 0–0.4 (samples **1**–**7**) exhibit a maximum of 10% Se vacancies (*avg*. 8%), defined as C_VSe_ = 1‐ $\frac{1}{2}\frac{\left[Se\right]}{\left[M\right]}$, where M = [Ru] + [Mo] + [V] + [Nb], which decreases to zero at *x*_Ru_ = 1 (see Table S1). The UA samples show 1%–2% Se vacancies, indicating that annealing induces more Se vacancies.

The crystal phase was examined using X‐ray diffraction (XRD) patterns, as shown in Figure 2a. Sample **1** exhibits the 2H MoSe_2_‐like ternary alloy phase (simply referred to as 2H) with lattice constants of (*a*_2H_, *c*_2H_) = (3.34, 13.0) Å. The XRD peaks of RuSe_2_ (sample **9**) correspond to the C phase with a lattice constant *a*_C_ = 5.92 Å, which is consistent with the reference value (*a*_C_ = 5.933 Å, JCPDS No. 65‐3328). All quaternary alloy samples **2**–**8** show the 2H phase with *a*_2H_ = 3.3 Å. In sample **5** (*x*_Ru_ = 0.25), the C phase RuSe_2_ begins to appear. As *x*_Ru_ increases further, the peak intensity of C phase RuSe_2_ increases. The C phase peaks are marked by green bars. Notably, sample **7** (*x*_Ru_ = *x*_Mo_ = 0.4) contains a higher Mo content than sample **6** (*x*_Ru_ = 0.4, *x*_Mo_ = 0.2), resulting in a stronger intensity of the 2H phase peaks relative to the C phase RuSe_2_ peaks. These results indicate that Ru incorporation induces a phase transition to the C phase starting at *x*_Ru_ = 0.25.

**FIGURE 2:**
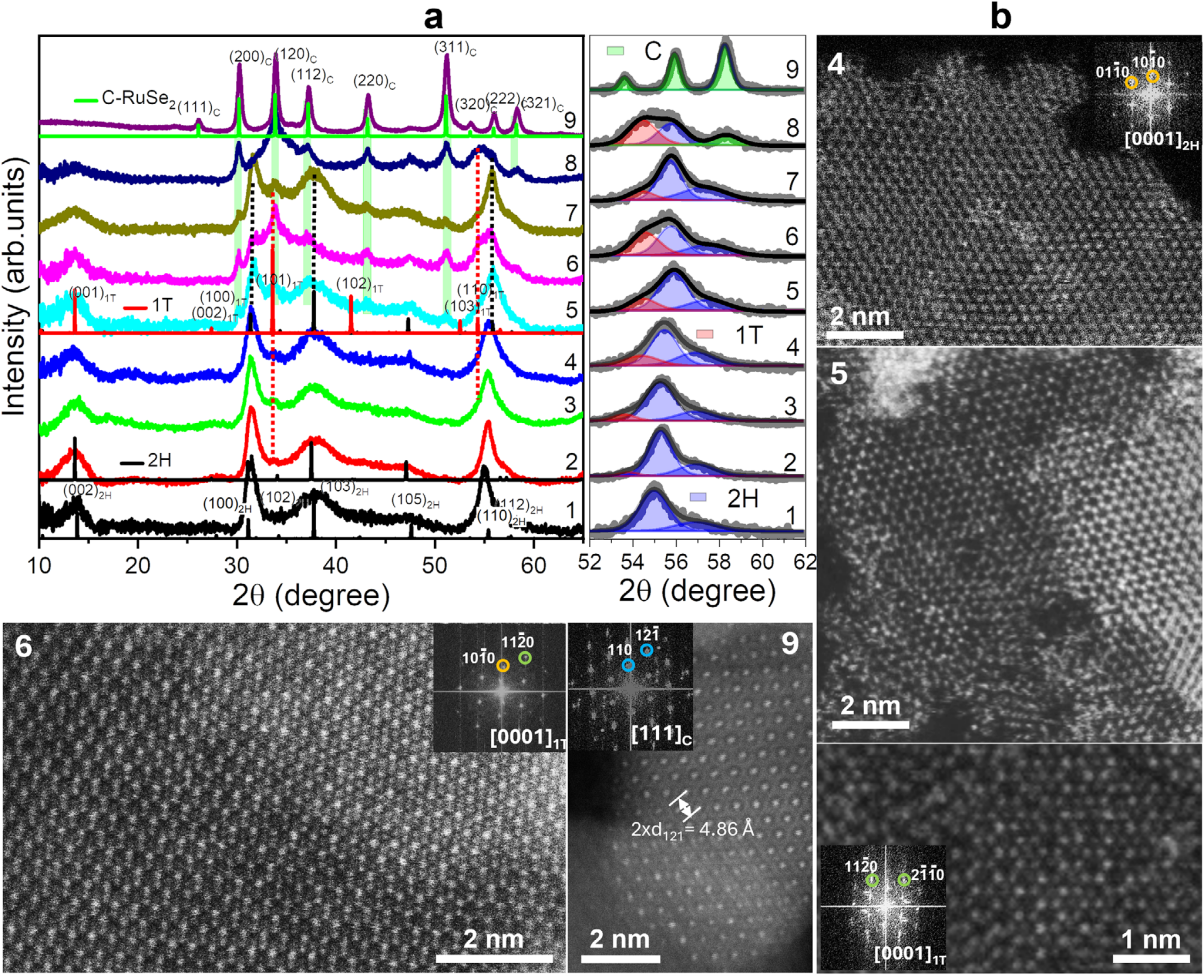
(a) Left: XRD patterns of samples **1**–**9** (600A), together with the reference peaks generated using the lattice constants of 2H phase (*a* = 3.34 Å and *c* = 13.0 Å) for sample **1**, and C phase RuSe_2_ (*a*_C_ = 5.92 Å) for sample **9**, and 2H phase (*a*_2H_ = 3.29 Å and *c*_2H_ = 13.0 Å) and 1T phase (*a*_1T_ = 3.38 Å and *c*_1T_ = 6.5 Å) for sample **5**. Right: XRD peaks at 2θ = 52°–62°, with the resolved 2H, 1T, and C phase bands. (b) Atomic‐resolution HAADF STEM images and corresponding FFT images for samples **4** (*x*_Ru_ = 0.14), **5** (*x*_Ru_ = 0.25), **6** (*x*_Ru_ = 0.4), and **9** (*x*_Ru_ = 1).

The quaternary alloy samples **3**–**8** exhibit peaks at 2θ = 54.5° that do not correspond to those of the C phase RuSe_2_. As *x*_Ru_ increases, this peak becomes intense. The XRD pattern of 1T phase with lattice constants *a*_1T_ = 3.38 Å and *c*_1T_ = 6.5 Å was plotted for sample **5** in the figure. The XRD peak of 2H phase is also plotted. The unassigned peak can be attributed to (110)_1T_ peak. The peak at 2θ = 33.7° could originate from (101)_1T_. The right panel corresponds to the phase‐resolved XRD peaks in the range of 2θ = 52°–62°. The intensity of 1T phase (110)_1T_ peak increases with increasing *x*_Ru_. The 2H phase peaks correspond to (110)_2H_ and (112)_2H_.

Figure S2 shows the XRD patterns of the entire UA and 400A samples. RuSe_2_ exhibits both 2H and 1T phases, with lattice constants *a*_2H_ = *a*_1T_ = 3.42 Å, *c*_2H_ = 12.58 Å, and *c*_1T_ = 6.29 Å. The 2H/1T → C phase transformation occurs upon annealing at elevated temperatures, in agreement with previous reports [28, 31, 41]. SEM and HRTEM images confirm nanosheet morphologies for the UA and 400A RuSe_2_ samples (Figure S1). The UA and 400A alloys exhibit a higher fraction of the 1T phase than their 600A counterparts. The phase assignments were further validated by Raman spectroscopy (Figure S3). These results suggest that the 2H → 1T phase transition of quaternary alloys originated from the 1T phase RuSe_2_. The preservation of the 2D layered structure over a wide composition range and the successful formation of the quaternary alloy are attributed to the layered TMD crystal structures of all constituents (RuSe_2_, MoSe_2_, VSe_2_, and NbSe_2_).

Figure 2b shows atomic‐resolution high‐angle annular dark‐field (HAADF) STEM images of samples **4**, **5**, **6**, and **9** Ru (Z = 44), Mo (Z = 42), and Nb (Z = 41) atoms could not be distinguished due to their similar atomic numbers. Darker regions corresponding to V atoms were not observed as separate domains at any composition. The homogeneous distribution of metal atoms suggests that quaternary alloying occurs at the atomic scale. The hexagonal atomic arrangement of the 2H phase is identified in sample **4**. The corresponding fast Fourier transform (FFT) image shows the {011_0} reflection spots of the 2H phase. The FFT analysis provides *d*_010_ = 2.9 Å, corresponding to a lattice constant *a* = 3.3 Å, in good agreement with the XRD data. In sample **5**, the major phase is the 2H phase, with the 1T phase coexisting (bottom). The atoms located at the centers of the hexagonal rings indicate the presence of the 1T phase. The corresponding FFT image reveals much stronger {112_0}_1T_ spots along the [0001]_1T_ zone axis compared to the 2H phase, indicative of 1T phase. The FFT analysis yields *d*_220_ (1T) = 1.7 Å, corresponding to *a*_1T_ = 3.4 Å. In sample **6**, the 1T phase region becomes more dominant relative to sample **5**. The FFT image over a large 1T phase region shows strong {112_0}_1T_ reflection spots at the [0001]_1T_ zone axis. Sample **9** mainly consists of the C phase. The FFT images show {110}_C_ and {121}_C_ reflection spots at the [111]_C_ zone axis, yielding *d*_121_ = 2.43 Å corresponding to *a*_C_ = 5.95 Å.

Figure S4 presents the XPS data for all samples (600A, 400A, and UA). Analysis of Ru 3*d*, Mo 3*d*, Nb 3*d*, V 2*p*, Se *3d*, and valence band spectra (VBS) indicate that incorporation of Ru into (MoVNb)Se_2_ enhances the metallic character, accompanied by lower oxidation states of V and Nb. The phase conversion into more metallic 1T phase probably contributes to increasing the metallicity at higher *x*_Ru_. The C phase RuSe_2_ of sample **9** is more metallic than the 2H/1T phase RuSe_2_, leading to quaternary alloys that are more metallic in the 600A samples than in the UA and 400A samples.

Figure 3a shows the EXAFS above the Ru, Mo, Nb, and Se K‐edge for samples **1**, **6**, and **9**. In the Ru‐Kedge X‐ray absorption near‐edge structure (XANES) spectra, sample **9** exhibits a red shift relative to sample **6**, likely due to the higher metallic character of C phase RuSe_2_ compared with the alloy. The Mo and Nb XANES spectra show a red shift for sample **6** compared to sample **1**, consistent with the increased metallicity of the quaternary alloys. The Fourier‐transform extended XAFS (FT EXAFS) of the Ru K edge shows no Ru‐Ru peak, confirming that the Ru atoms coordinated with Se atoms. The higher intensity of the Ru‐Se peak in sample **9** compared to sample **6** is attributed to the phase change. For Mo, Nb, and Se, the metal–Se peaks exhibit lower intensities in sample **6** than in sample **1**, probably due to a reduced number of bonds and a lower degree of crystallinity. The fitting curves and corresponding fitting parameters are summarized in Figure S5 and Table S2, respectively. The Se K‐edge peak was fitted using Mo‐Se, Nb‐Se, V‐Se, Nb‐Se, and Ru‐Se scattering paths, demonstrating the compositional evolution from the ternary alloy (sample **1**) to the quaternary alloys (sample **6**) and the cubic phase observed in sample **9**. As shown in Figure S5, the Se K‐edge data for samples **5**, **7**, and **8** further support this peak analysis.

**FIGURE 3:**
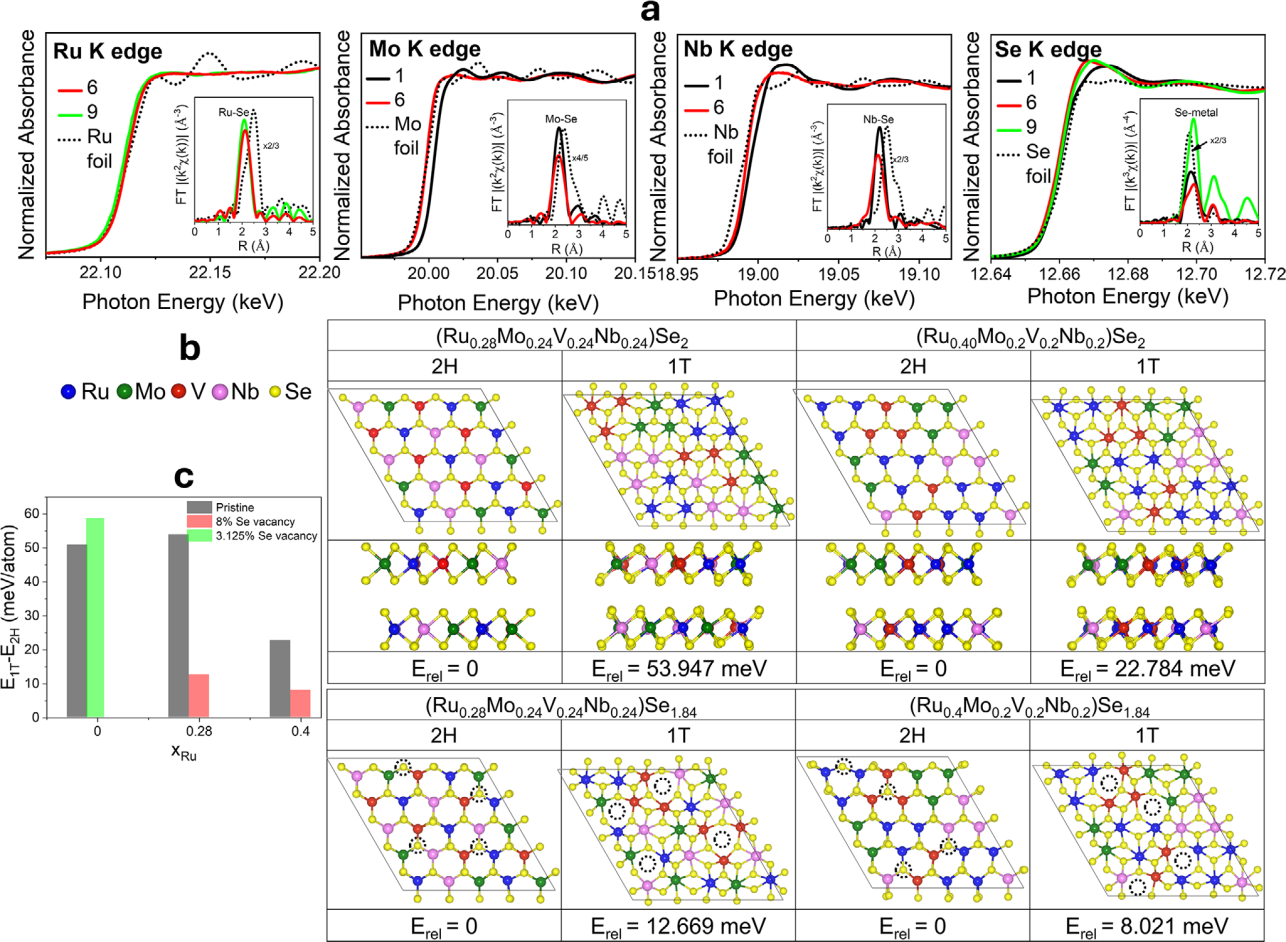
(a) XANES above the Ru K‐edge (22.1 keV), Mo K‐edge (20.01 keV), Nb K‐edge (18.98 keV), and Se K‐edge (at 12.64 keV) for samples **1**, **6**, and **9**, and corresponding non‐phase‐corrected FT EXAFS. (b) 2H and 1T phase crystal structure of the pristine and 8% Se vacancy models for (Ru_0.28_Mo_0.24_V_0.24_Nb_0.24_)Se*_x_* (*x* = 2 or 1.84 at *x*_Ru_ = 0.28) and (Ru_0.4_Mo_0.2_V_0.2_Nb_0.2_)Se*_x_* (*x* = 2 or 1.84 at *x*_Ru_ = 0.4). Their relative energies per atom are given. Blue, green, red, pink, and yellow balls represent Ru, Mo, V, Nb, and Se atoms, respectively. The Se vacancies are marked by dotted circles. (c) Relative energy (E_1T_ − E_2H_) of (Mo_0.375_V_0.3125_Nb_0.3125_)Se*_x_* (*x* = 2 or 1.94) ternary alloys [ref. 21] and quaternary alloys at *x*_Ru_ = 0.28 and 0.4 for pristine and Se vacancy models.

Density function theory (DFT) calculations incorporating spin polarization were performed for (Ru_0.28_Mo_0.24_V_0.24_Nb_0.24_)Se_2_ (*x*_Ru_ = 0.28) and (Ru_0.4_Mo_0.2_V_0.2_Nb_0.2_)Se_2_ (*x*_Ru_ = 0.4) using a (5 × 5 × 2) supercell containing 150 atoms. The model consisted of two atomic layers, and a vacuum region was added along the *c*‐axis instead of infinite replication. These two compositions were selected to represent samples **5** and **6**. Figure 3b illustrates the atomic arrangements of the 2H and 1T phases, viewed along the *c*‐axis (top view) and *a*‐axis (side view). Structural models were constructed by examining a minimum of six different configurations to identify the most energetically favorable atomic arrangements, as depicted in Figure S6. The lattice parameters are summarized in Table S3. Both the 2H and 1T phases exhibit a random distribution of all metal atoms, consistent with the STEM images. At *x*_Ru_ = 0.28 and 0.4, the 2H phase is stabilized over the 1T phase by 53.947 and 22.784 meV/atom, respectively. Therefore, as *x*_Ru_ increases, the phase conversion to the 1T phase becomes more favorable, in agreement with the experimental observations.

EDX analysis revealed an average of 8% Se vacancies for *x* = 0–0.4 samples, so configurations with four Se vacancies in each layer (corresponding to 8%) were examined. With Se vacancies included, the relative phase energies (E_1T_–E_2H_) are 12.669 and 8.021 meV/atom at *x*_Ru_ = 0.28 and 0.4, respectively. The configuration without Se vacancies is referred to as the pristine model. Se vacancies preferentially occupy V sites, which explains the significant reduction of Se vacancies at *x*_Ru_ = 0.7. In previous work, the relative energies for the 1T phase in pristine and 3.125% Se‐vacancy ternary alloy models were calculated using two‐layer (4 × 4 × 1) supercells. The E_1T_ – E_2H_ values for both ternary and quaternary alloys are summarized in Figure 3c. The Se vacancies of quaternary alloys substantially increase the stability of the 1T phase.

The electrocatalytic HER performance of the samples was measured using 1 M KOH, 0.5 M H_2_SO_4_, and 1 M PBS electrolytes. Table S4 summarizes the measured parameters. Figure 4a presents linear sweep voltammetry (LSV) curves of the samples in 1 M KOH electrolytes. The current density (*J*) was measured at a scan rate of 2 mV s^−1^ as the applied potential (vs. reversible hydrogen electrode, RHE) was varied. The overpotential (η) required to achieve *J* = 10 mA cm^−2^ is denoted as η*_J_*_= 10_. The ternary alloy (sample **1**) exhibits the highest η*_J_*_= 10_ value (265 ± 5 mV). In contrast, the quaternary alloying at *x*_Ru_ = 0.16–0.4 (samples **4**–**7**) significantly reduces η*_J_*_= 10_ to 36–40 mV. The Tafel slope (*b*) was determined by linear fitting of the Tafel plot (η vs. log* J*). Samples **4**–**7** display much lower *b* values (61–72 mV dec^−1^) compared to sample **1** (106 mV dec^−1^). Sample **6** is the best sample, which is characterized with η*_J_*_= 10_ = 36 ± 2 mV and *b* = 61 ± 1 mV dec^−1^. These values are similar to those of sample **9**. By comparison, commercial 20 wt.% Pt/C exhibits η*_J_*_= 10_ = 21 ± 1 mV and *b* = 41 ± 2 mV dec^−1^.

**FIGURE 4:**
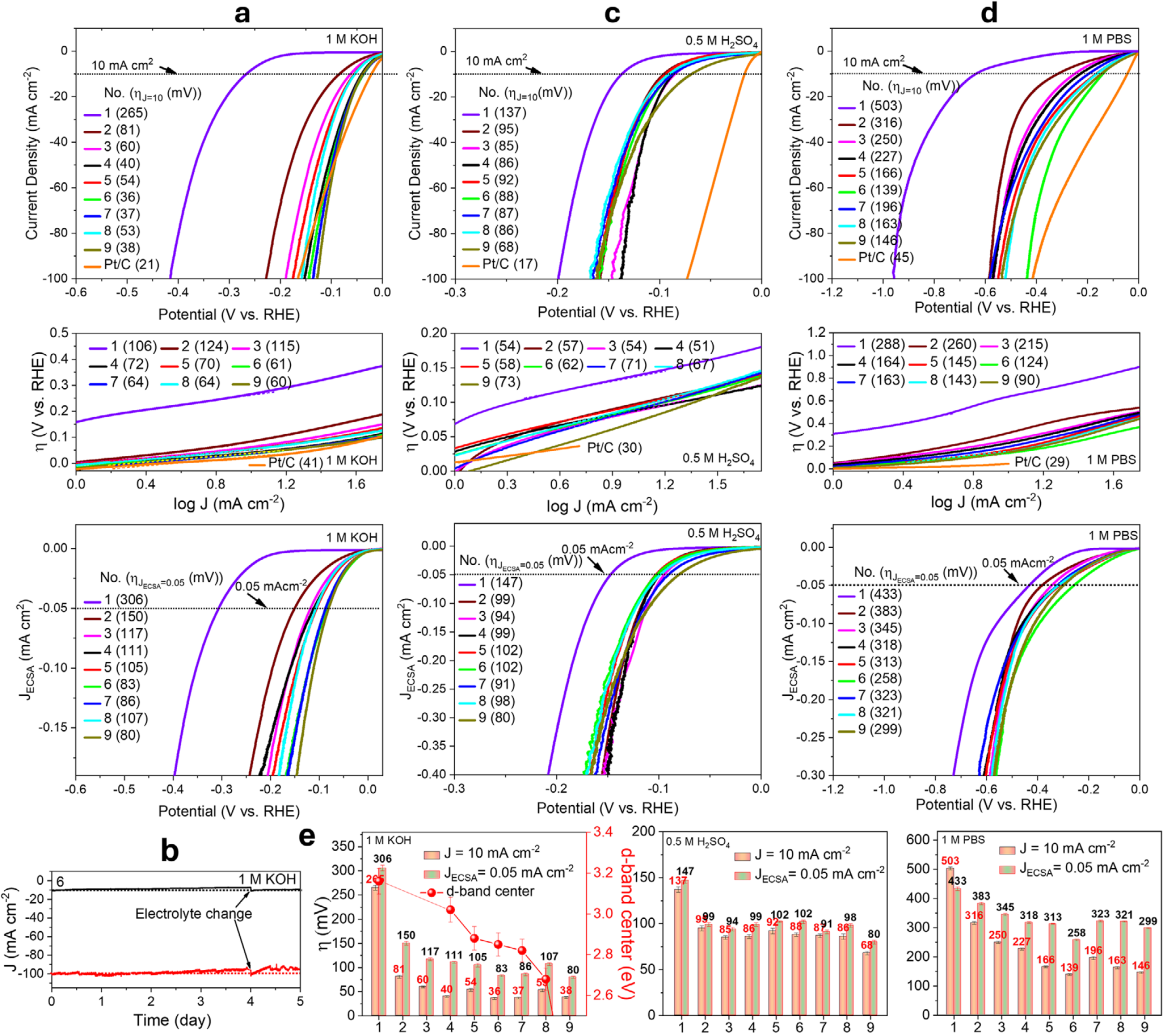
LSV curves (scan rate: 2 mV s^−1^) vs. RHE, Tafel plot, and ECSA‐corrected LSV curves vs. RHE for samples **1**–**9** and Pt/C toward HER in H_2_‐saturated (a) 1 M KOH, (c) 0.5 M H_2_SO_4_, and (d) 1 M PBS (pH 7.4). Tafel plots (η vs. log *J*) derived from the LSV curves, based on the equation η = *b* log(*J*/*J*_0_), where *b* is the Tafel slope, and *J*_0_ is the exchange current density (extrapolated value at η = 0). Linear fit provides the *b* value. The values in parenthesis correspond to η*_J_*_= 10,_ *b*, and η*_J_*_ECSA = 0.05_. (b) CA responses of *x*_Ru_ = 0.4 (sample **6)** at η*_J_*_= 10_ (36 ± 2 mV) and at η*_J_*_= 100_ (144 ± 3 mV) in 1 M KOH for 5 days. After 4 days, the electrolyte was replaced with a fresh one. (e) η*_J_*_= 10_ and η*_J_*_ECSA = 0.05_ of sample **1**−**9** for 1 M KOH, 0.5 M H_2_SO_4_, and 1 M PBS electrolytes. The *d*‐band center values are plotted using the right axis in plot of the KOH data. The data presents mean ± standard deviation from an average of measurements of at least three samples, repeating three or more times.

Figures S7 and S8 present electrochemical impedance spectroscopy and cyclic voltammetry data, respectively, which provide the charge transfer resistance (*R*_ct_) and double‐layer capacitance (*C*_dl_). Samples **2**–**9** exhibit lower *R*_ct_ values than sample **1**, indicating that improved HER performance is attributed to more efficient charge transfer. The electrochemically active surface area (ECSA) was estimated from *C*_dl_, as described in the Supporting Information (Experimental Section). The LSV curves were normalized by ECSA (*J*_ECSA_, defined as *J* divided by ECSA). The overpotential (η*_J_*_ECSA = 0.05_) required for a current density of 0.05 mAcm^−2^ confirmed the HER enhancement of the quaternary alloying. Chronoamperometry (CA) measurements for sample **6** at η*_J_*_= 10_ and η*_J_*_= 100_ revealed negligible current attenuation after 5 days (Figure 4b). The current recovered upon replacing the electrolyte after 4 days, indicating that the performance degradation is likely caused by a pH decrease in electrolyte. XRD, EDX, and XPS analyses confirmed that the composition and phase of the samples remained unchanged, with no Pt contamination detected after the CA test (Figure S9). Literature comparison of RuX_2_ catalysts is provided in Table S5.

The LSV curves measured in 0.5 M H_2_SO_4_ are shown in Figure 4c. Sample **1** exhibits the highest η_J = 10_ value (137 ± 3 mV). Quaternary alloying enhances the HER performance, as evidenced by lower η_J = 10_ values of 82–95 mV. The exchange‐current density (at η = 0) decreases significantly upon quaternary alloying. The η*_J_*_= 10_ and *b* values for samples **2**–**9** are similar. By comparison, commercial 20 wt.% Pt/C displays η*_J_*_= 10_ = 17 ± 1 mV and *b* = 30 ± 2 mV dec^−1^. The ECSA‐normalized LSV curves with η*_J_*_ECSA = 0.05_ values also showed the significantly enhanced HER performance of samples **2**–**9** compared to sample **1**. The HER performance data measured in 1 M phosphate buffer solution (PBS, pH 7.4) is shown in Figure 4d. Samples **1** and **6** exhibit the highest and lowest η_J = 10_ value (503 ± 5 and 139 ± 3 mV, respectively). Quaternary alloying markedly decreases the Tafel slope. For comparison, commercial 20 wt.% Pt/C shows η*_J_*_= 10_ = 145 ± 3 mV and *b* = 29 ± 1 mV dec^−1^. The ECSA‐normalized LSV curves and η at a normalized current density of *J*_ECSA_ = 0.05 mAcm^−2^ also confirm the significantly enhanced HER performance of the quaternary alloy samples. The corresponding *R*_ct_ and *C*_dl_ values are presented in Figures S7 and S8, respectively.

The η*_J_*_= 10_ and η*_J_*_ECSA = 0.05_ measured in three different electrolytes are summarized in Figure 4e. Overall, sample **6** shows significantly enhanced HER activity compared to sample **1**, with a performance similar to sample **9**. Increasing *x*_Ru_ beyond 0.4 does not further improve HER performance, indicating that alloying at *x*_Ru_ = 0.4 is optimal for maximizing HER activity, which highlights a key advantage of quaternary alloying. The HER performance of the UA and 400A samples is shown in Figure S10. Quaternary alloy samples exhibit markedly improved HER activity over ternary alloy samples. The HER performance of the UA and 400A samples is a little lower than that of the 600A samples.

The *d*‐band center (ε_d_) theory has often been used to explain optimal HER performance [21, 22, 23, 24, 35, 43, 44, 45] As ε_d_ decreases, the catalyst–hydrogen interaction becomes stronger due to enhanced metallic character. We estimated the ε_d_ values by integration of the VBS from 0 to 10 eV (see Figure S4). Ru incorporation reduces the ε_d_ value, as shown in the left panel of Figure 4e. The lower η*_J_*_= 10_ or η*_J_*_ECSA = 0.05_ values observed for the quaternary alloys compared to the ternary alloy correlate with their smaller ε_d_ values. Samples **1** and **6** display ε_d_ values of 3.16 ± 0.09 eV and 2.85 ± 0.09 eV, respectively. The increased catalytic activity of the 600A samples relative to the 400A and UA samples similarly results from their smaller ε_d_ values. Although the ε_d_ value of sample **6** is larger than that (2.08 ± 0.06 eV) of sample **9**, their HER performance is comparable, likely due to the nanosheet morphology maximizing surface area and exposing more active sites for HER. Another contributing factor is the Se vacancies, which expose highly active metal sites for HER. The Se vacancies (*avg*. 8% for *x*_Ru_ = 0.05–0.4) enhance the HER performance of the quaternary alloys. The decreased Se vacancies (5%) at *x*_Ru_ = 0.7 would reduce catalytic activity, diminishing the metallicity enhancement effect.

The Gibbs free energy of hydrogen adsorption (ΔG_H*_) serves as a key descriptor for evaluating HER catalytic activity, with ideal catalysts exhibiting |ΔG_H*_| values close to zero. Figure 5a presents the adsorption configurations of H atoms on basal Se sites of pristine 2H phase (Ru_0.28_Mo_0.24_V_0.24_Nb_0.24_)Se_2_ (*x*_Ru_ = 0.28) and 2H/1T phase (Ru_0.4_Mo_0.2_V_0.2_Nb_0.2_)Se_2_ (*x*_Ru_ = 0.4). The ΔG_H*_ values are 0.96, 0.74, and 0.47 eV, corresponding to H adsorption on Se atoms coordinated with 2Ru‐Mo (*x*_Ru_ = 0.28, 2H phase), 3V (*x*_Ru_ = 0.4, 2H phase), and 3Ru (*x*_Ru_ = 0.4, 1T phase), respectively. Figure S11 presents various H‐adsorbed intermediates for both the 2H and 1T phases. The ΔG_H*_ values for all 32 adsorption sites are plotted in the right panel of Figure 5a. For the 2H phase, ΔG_H*_ ranges from 0.96 to 1.55 eV at *x*_Ru_ = 0.28 and from 0.74 to 1.25 eV at *x*_Ru_ = 0.4, indicating that increasing the Ru content enhances HER activity. Furthermore, for the 1T phase at *x*_Ru_ = 0.4, the ΔG_H*_ values further decrease to 0.47–1.08 eV, suggesting that the 1T phase exhibits superior catalytic activity compared to the 2H phase. These results suggest that the phase transition to the more metallic 1T phase plays a crucial role in promoting HER performance.

**FIGURE 5:**
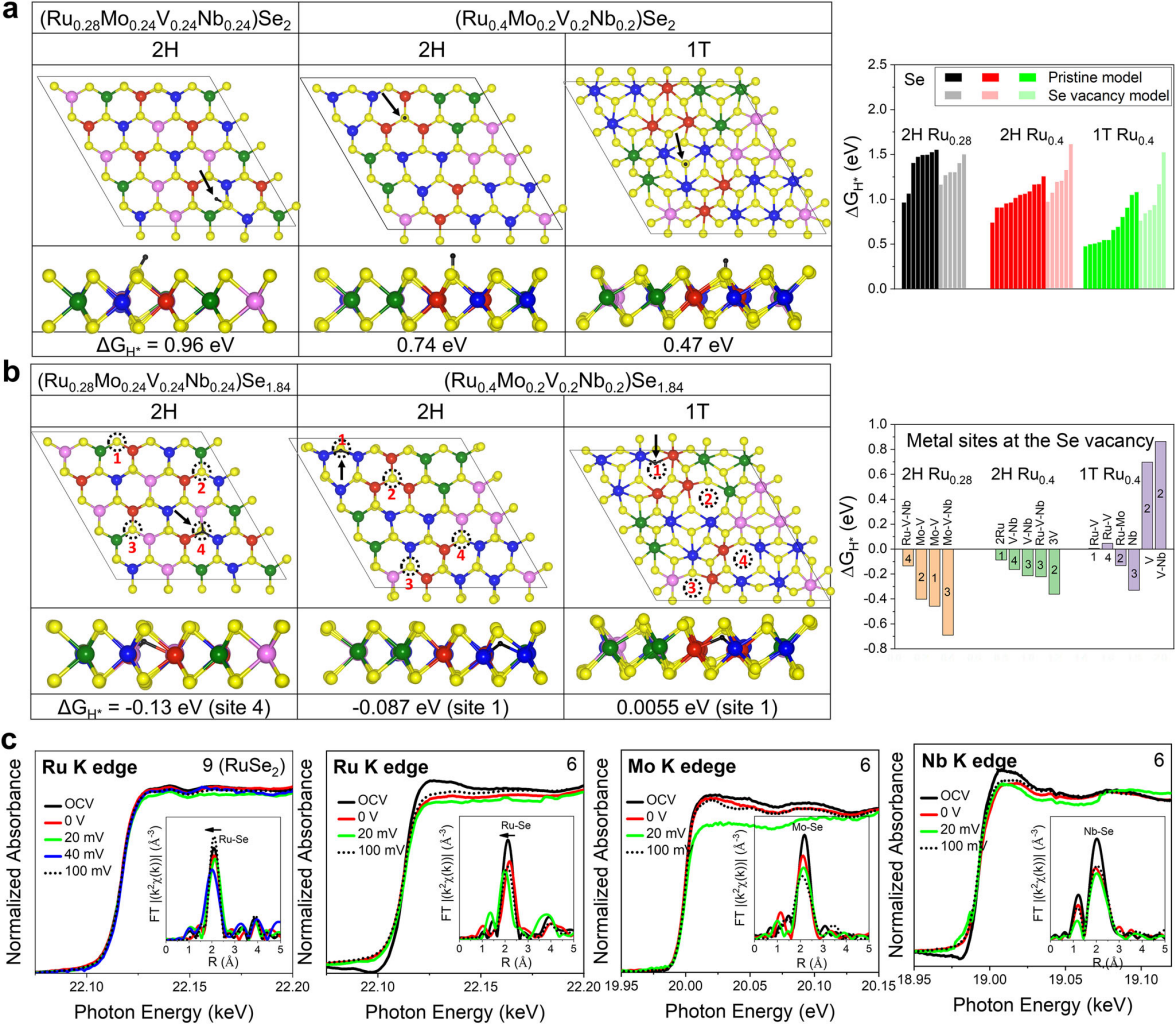
Geometry of the H adsorption on (a) the Se atoms of pristine model and (b) Se vacancy sites. The ΔG_H*_ value is given for each site. The Se vacancies are marked by dotted circles and numbered by sites **1**–**4**. Blue, green, red, pink, and yellow balls represent Ru, Mo, V, Nb, and Se atoms, respectively. The H atoms (black balls) are marked by arrows. Histograms on the right panel represent the ΔG_H*_ value for all sites calculated in the present work. For the Se vacancy model, H‐coordinated metals at the sites **1**–**4** are marked in the histogram. (c) In situ/ex situ XANES above the Ru K‐edge, Mo K‐edge, and Nb K‐edge for samples **6** and **9**, and their corresponding non‐phase‐corrected FT EXAFS, under (in situ) 𝜂 = 0–40 mV (vs. RHE) and (ex situ) 𝜂 = 100 mV after 20 min, in H_2_‐saturated 1 M KOH.

Using the Se vacancy model of the 2H phase (*x*_Ru_ = 0.28 and 0.4) and the 1T phase (*x*_Ru_ = 0.4), H adsorption was investigated at four Se vacancy sites (labeled **1**–**4**), as shown in Figure 5b. When the H atom binds to Ru─V─Nb atoms (site **4** of the 2H phase at *x*_Ru_ = 0.28), 2Ru atoms (site **1** of the 2H phase at *x*_Ru_ = 0.4), and Ru─V atoms (site **1** of the 1T phase at *x*_Ru_ = 0.4), the corresponding ΔG_H*_ values approach zero: −0.13, −0.087, and 0.0055 eV, respectively.  All these configurations involve coordination with at least one Ru atom, highlighting Ru as the most catalytically active metal species. H adsorption at other Se vacancy sites also yields comparably small |ΔG_H*_| values (see Figure S11). The right panel of Figure 5b plots the ΔG_H*_ values for H adsorption at sites **1**–**4**, which are significantly lower than those for adsorption on basal Se atoms (plotted in the right panel of Figure 5a). These results demonstrate that the presence of Se vacancies markedly enhances HER performance. Overall, the calculated |ΔG_H*_| values confirm that Ru incorporation in the 1T phase optimizes HER catalytic activity, achieving the best performance at *x*_Ru_ = 0.4 due to the optimal Se‐vacancy concentration, before declining at higher Ru contents (*x*_Ru_ = 0.7).

To elucidate the electronic structures around metal atoms during alkaline HER, in situ XAFS measurements were conducted on samples **6** and **9**. Figure 5c displays in situ XANES and FT EXAFS spectra at the Ru, Mo, and Nb K‐edge under applied overpotentials (η) of 0–20 mV (sample **6**) or 0–40 mV (sample **9**) in 1 M KOH. Open‐circuit voltage (OCV) data were acquired prior to potential application. Ex situ data were collected after 20 min at η = 100 mV, as excessive H_2_ evolution precluded in situ measurements at higher η; notably, η = 40 mV data for sample **6** were also unobtainable due to H_2_ bubble interference. For Ru XANES, the white‐line intensity decreases with increasing η, more markedly for sample **6** than sample **9**. This suggests that sample **6** becomes more metallic under applied potential, probably related with nanosheet morphology.

EXAFS fitting (Figure S5 and Table S2) reveals a Ru─Se bond contraction (Δ*d*_Re‐Se_ = 0.02 Å at η = 40 mV) for sample **9**, likely attributable to electrolyte adsorption such as H_2_O or OH^−^. In contrast, sample **6** exhibits significant Ru─O formation (*d*_Re‐O_ = 1.94 Å) arising from H_2_O/OH^−^ adsorption, along with Ru─Se bond contraction (Δ*d*_Re‐Se_ = 0.02 Å at η = 20–100 mV). These results indicate that electrolyte adsorption is more effective in sample **6** than in sample **9**. In the XANES spectra of Mo and Nb (sample **6**), the white‐line intensity similarly decreases with increasing overpotential, signaling enhanced metallicity for both elements during HER. Negligible change in the Mo─Se and Nb─Se bond distance suggests that these atoms are involved in the reduction processes rather than in electrolyte adsorption. Thus, the superior HER performance of the quaternary alloy samples arises from synergistic metallicity enhancement across metals, coupled with Ru‐mediated electrolyte (H_2_O/OH^−^) adsorption under reductive potentials—effects amplified by the nanosheet morphology. These findings align with DFT‐derived ΔG_H*_ trends, confirming Ru incorporation as a key driver of optimized HER activity.

## 3 Conclusions

Composition‐tuned (RuMoVNb)Se_2_ quaternary alloy nanosheets with *x*_Ru_ ranging from 0 to 1 were synthesized via a colloidal reaction. Ru Incorporation into (MoVNb)Se_2_ ternary alloys induced a 2H → 1T phase transition while preserving nanosheet morphology across the composition range. RuSe_2_ exhibited a 2H/1T → C phase upon annealing (up to 600°C); thus, annealing increases the C‐phase fraction in quaternary alloys. The 2H → 1T phase transition of alloys probably originated from the 1T phase RuSe_2_. Atomic‐resolution STEM images confirmed homogeneous atomic mixing within the layered structure, and the production of 1T phase with increasing *x*_Ru_. XPS and XAFS analyses indicated that Ru incorporation into the ternary alloy produced a more metallic phase with less surface oxidation. DFT calculations validate uniform atomic mixing at *x*_Ru_ = 0.25 and 0.4 and thermodynamically favored 2H → 1T transition at higher *x*_Ru_. Quaternary alloying enhanced the electrocatalytic HER activity across 1 M KOH (η*_J =_*_10_ = 36 mV vs. RHE), 0.5 M H_2_SO_4_, and 1 M PBS. The ΔG_H*_ calculations using pristine and Se vacancy models confirmed Ru‐optimized H adsorption. In situ XAFS data (in 1 M KOH) suggested that superior HER performance is attributed to multi‐metal metallicity and Ru‐facilitated electrolyte adsorption.

## 4 Methods

### 4.1 Synthesis of (RuMoVNb)Se_2_ Nanosheets

Ruthenium chloride (RuCl_3_), molybdenum chloride (MoCl_5_), niobium chloride (NbCl_5_), vanadium chloride (VCl_3_,) (total amount: 0.5 mmol) were prepared as metal precursors. The metal precursor and 1 mmol of dibenzyl diselenide (PhCH_2_)_2_Se_2_) were dissolved in 5 mL of oleylamine (OAm) and degassed at 70°C. 5 mL of oleylamine (OAm; C_18_H_35_NH_2_) in a three‐necked flask was degassed and heated at 260°C. The precursor solution (2 mL) was injected into the preheated OAm with an injection rate of 0.4 mL min^−1^ for 5 min, and the mixture was reacted for 1 h. After washing, the sulfide/selenide products were annealed in a quartz tube inside electricaly heated furnace under Ar flow at 400°C or 600°C for 1 h.

## Author Contributions

**Ju Yeon Kim**: conceptualization, investigation, software, data curation. **Jeong Eun An**: formal analysis. **Ik Seon Kwon**: formal analysis, resources, investigation, software, writing – review and editing, validation, data curation. **Youn Jun Choi**: formal analysis. **Junaid Ihsan**: conceptualization, investigation, methodology, data curation. **Hong Seok Kang**: conceptualization, investigation, resources, supervision. **Doyeon Kim**: resources, formal analysis, software. **Irtiqa Mishal**: investigation, software, data curation, methodology. **Jun Hyeok Choi**: formal analysis. **Jeunghee Park**: conceptualization, investigation, resources, supervision, software, writing – review and editing, writing – original draft, funding acquisition. **In Hye Kwak**: resources, formal analysis. **Gianvito Vilé**: formal analysis, resources, funding acquisition.

## Conflicts of Interest

The authors declare no conflicts of interest.

## Supporting information




**Supporting File**: advs76600‐sup‐0001‐SuppMat.docx.

## Data Availability

The data that support the findings of this study are available in the supplementary material of this article.
