# Supplementary material for: Two‐Dimensional Quaternary Alloys of (RuMoVNb)Se2 for Superior Hydrogen Evolution Reaction Catalysis
Source: Adv Sci (Weinh). 2026 Jul 24:e76600. Online ahead of print. doi: 10.1002/advs.76600 (PMC13397828; doi:10.1002/advs.76600)
Supplement: Supplementary file 1 — Supporting File: advs76600‐sup‐0001‐SuppMat.docx. [file ADVS-9999-e76600-s001.docx]

*Supplementary Information*

Two-Dimensional Quaternary Alloys of (RuMoVNb)Se₂ for Superior Hydrogen Evolution Reaction Catalysis

Junaid Ihsan,^†^ Ju Yeon Kim,^†^ Irtiqa Mishal,^†^ Jun Hyeok Choi,^†^ Jeong Eun An,^†^ Youn Jun Choi,^†^ Doyeon Kim, ^‡^ In Hye Kwak,*^,≠^ Gianvito Vilé,^$^ Ik Seon Kwon,*^,§^ Hong Seok Kang,*^,#^ and Jeunghee Park*^,‡^

^‡^ Department of Advanced Materials Chemistry, Korea University, Sejong 339-700, Republic of Korea; *E-mail: [parkjh@korea.ac.kr](mailto:parkjh@korea.ac.kr)

^‡^ Korea Research Institute Standard and Science Daejeon 34133, Republic of Korea

^≠^ Research Center for Materials Analysis, Korea Basic Science Institute (KBSI), Daejeon 34133, Republic of Korea; Email: [ihkwak318@kbsi.re.kr](mailto:ihkwak318@kbsi.re.kr)

^$^ Department of Chemistry, Materials, and Chemical Engineering ‘‘Giulio Natta’’, Politecnico di Milano, Piazza Leonardo da Vinci 32, 20133 Milano, Italy

^§^ Department of Energy Science & Engineering, Kunsan National University, 558 Daehak-ro, Gunsan, Republic of Korea; Email: [iskwon@kunsan.ac.kr](mailto:iskwon@kunsan.ac.kr)

*^#^* Department of Nano and Advanced Materials, Jeonju University, Chonju, Chonbuk 55069, Republic of Korea; Email: [hsk@jj.ac.kr](mailto:hsk@jj.ac.kr)

^†^ J. Ihsan, J. Y. Kim, and I. Mishal contributed equally as the first author.

**Contents**

**Experimental Section**

**Calculation Section**

**Statistical Analysis**

**Table S1**. Composition of nanosheets.

**Table S2**. Fitting parameters of EXAFS data.

**Table S3**. Calculated parameters of (5 × 5 × 2) supercell for (Ru_0.28_Mo_0.24_V_0.24_Nb_0.24_)Se*_x_* and (Ru_0.4_Mo_0.2_V_0.2_Nb_0.2_)Se*_x_*.

**Table S4**. Summary of HER performance of (RuMoVNb)Se_2_ nanosheets.

**Table S5**. Comparison of HER performance with the previous works.

**Figure S1**. SEM, HRTEM, and EDX data of (RuMoVNb)Se_2_ samples.

**Figure S2**. XRD of (RuMoVNb)Se_2_-400A and -UA samples.

**Figure S3**. Raman spectrum of (RuMoVNb)Se_2_ samples.

**Figure S4**. XPS data of (RuMoVNb)Se_2_ samples.

**Figure S5**. EXAFS fitting with XAFS data of sample 5, 7, and 8.

**Figure S6**. Various configurations of Ru_0.28_Mo_0.24_V_0.24_Nb_0.24_)Se*_x_* and (Ru_0.4_Mo_0.2_V_0.2_Nb_0.2_)Se*_x_*.

**Figure S7**. Nyquist plots of (RuMoVNb)Se_2_ samples.

**Figure S8**. Cyclic voltammetry curves of (RuMoVNb)Se_2_ samples.

**Figure S9**. Characterization of sample **6** after CA test.

**Figure S10**. HER performance of (RuMoVNb)Se_2_-400A and -UA samples.

**Figure S11**. Crystal structures of the HER intermediates.

**References**

**Experimental Section**

***Reagents***: All reagents were purchased (mainly from Sigma-Aldrich or Alfa Co) and used as received without further purification.

***Colloidal synthesis****.* A hot injection colloidal reaction was conducted using Schlenk line under Ar flow. 5 mL of oleylamine (OAm; C_18_H_35_NH_2_; molecular weight (MW) = 267.493 g mol^-1^, technical grade 70%, density = 0.813 g mL^-1^) in a three-necked flask was degassed at 110 °C for 30 min, then the temperature was raised to 260 °C. 0.5 mmol of RuCl_3_ (MW = 207.43 g mol^-1^, 99.98%), MoCl_5_ (MW = 273.21 g mol^-1^, 95%), VCl_3_ (MW = 157.3 g mol^-1^, 97%), and NbCl_5_(MW = 270.17 g mol^-1^, 99.995%) mixture, and 1 mmol of (PhCH_2_)_2_Se_2_ (dibenzyl diselenide; MW = 340.2 g mol^-1^, 95%) were dissolved in 5 mL OAm and the mixture was kept at 70 °C during 2h degassing. The molar ratio of metal precursors was changed by keeping a total 0.5 mmol. 2 mL of precursor solution was injected into the OAm solution in flask (prepared with an injection rate of 0.4 mL min^-1^ for 5 min, and the mixture was stirred at 260 °C. Total reaction time is 1 h. The reaction solution was cooled down to room temperature and the black products were separated by centrifugation. The products were washed with 1:1 (v/v) ethanol:toluene mixture by four times and dried using an evaporator. The product (powders) was placed in a quartz tube inside electrically heated furnace and annealed under Ar flow (flow rate = 200 sccm) at 400 or 600 °C for 1 h.

***Characterization****.* The products were characterized by high-resolution scanning electron microscopy (HR-SEM; SU8230, Hitachi; instrument ID: CJ113) and by field-emission transmission electron microscopy (FE-TEM; Libra 200 MC, Carl Zeiss; instrument ID: AE12) at the Korea Basic Science Institute (KBSI), Daejeon, Korea. Energy dispersive X-ray fluorescence spectroscopy (EDX) with elemental maps was measured using an Ultra Corrected Energy Filtering TEM operated at 200 kV that equipped with ZrO/W-field emitter system (Schottky emitter), EDS detector system (X-Max 80T, Oxford), and side CCD camera (ORIUS SC200D. Gatan). Fast Fourier-transform (FFT) images were generated by the inversion of the TEM images using Digital Micrograph GMS1.4 software (Gatan Inc.).

Spherical Aberration (Cs)-corrected scanning TEM (STEM) analysis was carried out using a Titan 80-300TM (FEI, The Netherlands) microscope operated at 200 kV. The STEM convergence semi-angle (α) used was ~18 mrad. The minimum and maximum acceptance semi-angles (β) were ~20 and 122 mrad, respectively. The dwell time per pixel was set to 0.7 µs for imaging. We used the low total dose and dwell time while measuring the STEM. The image was usually taken at a dose rate of 6 × 10^4^ e-/nm^2^·s with a total dose of 1.8 × 10^5^ e-/nm^2^. Elemental analysis was performed on an elemental analyzer (Elementar Analysensysteme GmbH, Model No. Elementar Vario EL cube). Raman spectra were measured with a micro-Raman spectrometer (Alpha 300R, WITecGmbH, Germany), using a 532 nm diode laser with a power of 0.5 mW.

High-resolution X-ray diffraction (XRD) patterns were obtained using the 3D beamlines of the Pohang Light Source (PLS)-II with monochromatic radiation (λ = 1.52150 Å). XRD pattern measurements were also carried out in a Rigaku D/MAX-2500 V/PC using Cu K_α_ radiation (λ = 1.54056 Å). X-ray photoelectron spectroscopy (XPS) measurements were performed using the 10A2 beam lines of the PLS-II. The photon energy of synchrotron radiation was calibrated using the standard sample; Au film, Au 4*f*_7/2_ peak at 84.0 eV. X-ray absorption near edge structure (XANES) measurements were performed using the 7D and 8C XAFS beamlines of the PLS-II. The X-ray beam was monochromatic using a Si(111) double crystal, where the harmonic rejection was attained by detuning the beamline optics so that the intensity of the incident beam was reduced by 30%. For all the measurements, the slits had an opening of 0.5 mm (vertical) × 1 mm (horizontal). All the measurements were performed at room temperature in transmission mode and the detectors were ionization-chamber-based. The monochromator energies were calibrated using Ru, Mo, V, and Nb foils, respectively. XANES analysis and EXAFS fitting were performed using the Athena and Artemis packages, which provide an interface to IFEFFIT.

***Electrochemical Measurements*.** Experiments were carried in a three-electrode cell connected to an electrochemical analyser (CompactStat, Ivium Technologies). HER electrocatalysis in 0.5 M H_2_SO_4_, 1 M KOH, and 1 M phosphate buffer solution (PBS) electrolyte was measured using a linear sweeping from 0 to -0.6 V (vs. RHE) with a scan rate of 2 mV s^–1^. Reference electrode was a saturated calomel electrode (SCE, KCl saturated, Pine Instrument) for 0.5 M H_2_SO_4_ electrolyte and an Ag/AgCl electrode (saturated with 4 M KCl, Pine Co.) for 1 M KOH/1 M PBS. A Pt coil (with fritted glass) was used as a counter electrode. The electrolyte was purged with H_2_ (ultrahigh grade purity 99.999%) during the measurement. The Pt counter electrode was encapsulated with a fritted glass tube (**Figure E1**), so that the Pt deposition on the working electrode was prevented.


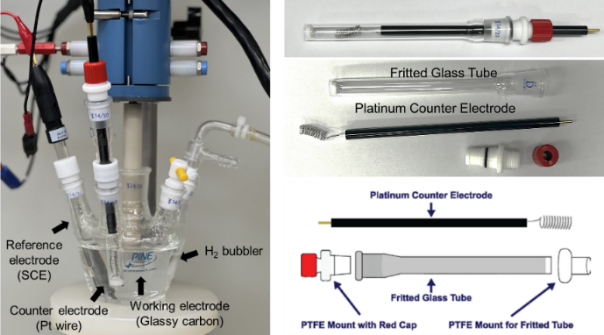


**Figure E1**. Photographs for our three-electrodes cell and Pt counter electrode encapsulated with a fritted glass tube, as shown on the right panel.

The applied potential (E) reported in our work was referenced to the reversible hydrogen electrode (RHE) through standard calibration as described elsewhere. We calibrate the potential of the reference electrode *vs.* standard hydrogen electrode (SHE). Cyclic voltammetry (CV) curves were obtained at a scan rate of 2 mV s^−1^, in the high-purity H_2_ saturated electrolyte with a Pt wire as the working electrode. The SCE and Ag/AgCl electrodes were used as reference electrodes for 0.5 M H_2_SO_4_ and 1 M KOH electrolytes, so the average value of the potential at which the current crossed at zero was -0.278 V and -0.197 V, respectively. In 0.5 M H_2_SO_4_, E (*vs.* RHE) = E (*vs.* SCE) + 0.278 V. In 1 M KOH, the measurements were referred to the RHE by using the relationship: E (*vs.* RHE) = E (*vs.* Ag/AgCl) + 0.197 V + 0.0592 V × pH 14 = E (*vs.* Ag/AgCl) +1.0258 V. In 1 M PBS, the measurements were referred to the RHE by using the relationship: E (*vs.* RHE) = E (*vs.* Ag/AgCl) + 0.197 V + 0.0592 V × pH 7 = E (*vs.* Ag/AgCl) + 0.6114 V.

4 mg sample was mixed with 2 mg carbon black (Vulcan XC-72) dispersed in Nafion (50 μL) and isopropyl alcohol (0.95 mL). The catalyst materials (0.65 mg cm^-2^) were deposited on a glassy carbon rotating disk electrode (RDE, area = 0.1963 cm^2^, Pine Instrument),), and a rotation speed of 1600 rpm was used for the linear sweep voltammetry (LSV) measurements. The Pt/C (20 wt.% Pt in Vulcan carbon black, Sigma-Aldrich) tested as reference sample using the same procedure. The LSV curves were reproducible for four separate loadings of samples on the GC RDE electrode. For chronoamperometric stability test and *in-situ/ex-situ* XAFS measurements, we fabricated the working electrode by depositing the samples (1 mg cm^-2^) on 1 × 1 cm^2^ area of hydrophilic/waterproof carbon cloth (WIZMAC Co., thickness = 0.35 mm, through-plane resistance = 1 mΩ) that was cut with a size of 1 × 3 cm^2^.

Electrochemical impedance spectroscopy (EIS) measurements were carried out for the electrode in an electrolyte by applying an AC voltage of 10 mV in the frequency range of 100 kHz to 0.1 Hz at a bias voltage of ~~-~~0.10 V and -0.20 V (vs. RHE) under 0.5 M H_2_SO_4_ and 1 M KOH/1 M PBS, respectively. To measure double-layer capacitance *via* cyclovoltammetry (CV), a potential range in which no apparent Faradaic processes occur was determined from static CV. All measured current in this non-Faradaic potential region is assumed to be due to double-layer capacitance. The charging current, *i_c_*, is then measured from CVs at multiple scan rates. The working electrode was held at each potential vertex for 10 s before beginning the next sweep. The charging current density (*i_c_*) is equal to the product of the scan rate (ν) and the electrochemical double-layer capacitance (C*_dl_*), as given by equation *i_c_* = ν C*_dl_*. The difference (ΔJ_0.15_) between the anodic charging and cathodic discharging currents measured at 0.15 V or 0.05 V (vs. RHE) was used for *i_c_* under 0.5 M H_2_SO_4_ and 1 M KOH/1 M PBS, respectively. Thus, a plot of ΔJ_0.15_ or ΔJ_0.05_ as a function of ν yields a straight line with a slope equal to 2 × C*_dl_* under 0.5 M H_2_SO_4_ and 1 M KOH, respectively. The scan rates were 20−100 mV s^-1^.

***ECSA calculation****.* To estimate the actual electrochemical surface area (ESCA), we used the *C*_dl_ value. The roughness factor (= ECSA) is defined as the surface area ratio between the catalyst and the flat TMD electrodes. This can be obtained using$\frac{C_{dl}}{C_{s}} ,$where C*_dl_* and C*_s_* are electrochemical double-layer capacitance of the catalysts (measured by the procedure described above) and the flat surface, respectively. The *C_s_* value is assumed to be 0.060 mFcm^-2^ for all samples.^S1^ The specific surface of the electrode can be estimated by multiplying the geometrical surface (A_geom_ = 0.1963 cm^2^) to ECSA; A_geom_ × ECSA.

**Table E1**. ECSA of (a) 600A and (c) 400A/UA samples, calculated using the equation of ECSA = *C_dl_*/*C*_s_, where C_s_ is the flat standard capacitor (60 μF cm^-2^).

(a)

| No. | 1 M KOH | | 0.5 M H_2_SO_4_ | | 1 M PBS | |
| --- | --- | --- | --- | --- | --- | --- |
|  | *C_dl_* (mF cm^-2^) | ECSA | *C_dl_* (mF cm^-2^) | ECSA | *C_dl_* (mF cm^-2^) | ECSA |
| 1 | 24.50 ± 0.81 | 408.16 | 18.85 ± 0.63 | 314.25 | 3.90 ± 0.29 | 65.00 |
| 2 | 38.09 ± 1.10 | 634.91 | 13.72 ± 0.42 | 228.75 | 17.69 ± 0.93 | 294.83 |
| 3 | 39.07 ± 1.21 | 651.25 | 18.34 ± 0.33 | 305.66 | 19.92 ± 0.64 | 332.00 |
| 4 | 45.19 ± 1.40 | 761.58 | 22.68 ± 0.77 | 378.08 | 23.56 ± 1.25 | 392.66 |
| 5 | 40.00 ± 1.22 | 666.66 | 13.61 ± 0.63 | 226.83 | 28.25 ± 1.43 | 470.83 |
| 6 | 64.96 ± 1.93 | 1082.75 | 20.43 ± 0.44 | 340.50 | 33.05 ± 1.72 | 550.83 |
| 7 | 46.65 ± 2.02 | 777.50 | 17.22 ± 0.58 | 287.05 | 25.19 ± 1.31 | 419.83 |
| 8 | 42.20 ± 1.34 | 703.33 | 15.67 ± 0.55 | 261.25 | 26.94 ± 1.35 | 449.00 |
| 9 | 43.60 ± 1.43 | 726.66 | 16.62 ± 0.42 | 277 | 29.89 ± 1.26 | 498.16 |

(b)

| No. | 1 M KOH | | 0.5 M H_2_SO_4_ | |
| --- | --- | --- | --- | --- |
|  | *C_dl_* (mF cm^-2^) | ECSA | *C_dl_* (mF cm^-2^) | ECSA |
| 1-400A | 20.09 ± 0.73 | 334.83 | 16.79 ± 0.57 | 279.83 |
| 6-400A | 41.60 ± 1.23 | 693.33 | 16.23 ± 0.48 | 270.50 |
| 9-400A | 42.85 ± 1.35 | 714.15 | 12.30 ± 0.43 | 205.08 |
| 6-UA | 42.60 ± 1.33 | 710.0 | 15.16 ± 0.54 | 252.66 |
| 9-UA | 32.39 ± 1.07 | 539.91 | 7.16 ± 0.36 | 119.41 |

**Calculation Section**

First-principles calculations were performed through spin-polarized density functional theory (First-principles calculations were performed through spin-polarized density functional theory (DFT). The Vienna Ab-initio Simulation Package (VASP)^S2,S3^ is mainly used for the calculations. The electron-ion interactions were described using the projector-augmented wave (PAW)^S4^ method with a plane-wave kinetic energy cutoff of 350 eV. The effect of attractive van der Waals (vdW) interaction was taken into account by employing Grimme’s D3 correction (PBE-D3).^S5,S6^ Both ionic and lattice relaxation were performed using the PBE-D3 exchange-correlation functional and PBE+U method. Monkhorst-Pack *k*-point sampling of 3×3×1 was used for geometry optimization. Structural optimization was performed until when the total-energy change between cycles is less than 1×10^-5^ eV.

The change in Gibbs free energy during the reaction provides useful information about the energy and spontaneity of the reaction (whether it can happen without additional energy). Under standard conditions, the HER consists of two steps, the adsorption of H on the catalyst in the Volmer reaction (*), followed by the Heyrovsky/Tafel reaction to release hydrogen molecules (1/2 H_2_ +*). Therefore, we should construct the Gibbs free energy profile along the reaction coordinate by calculating the relative free energies of the reactant and intermediates in the Volmer reaction. The Gibbs free energy profile along the reaction coordinate can be calculated according to the equation.^S7^

$$\Delta G= {\Delta E}_{DFT}+{\Delta E}_{ZPE}+ {\Delta H}_{corr}-T\Delta S$$

where $E_{DFT}$ is the total energy of hydrogen atom adsorption calculated from DFT, $\Delta E_{ZPE}$ is the zero point energy change, ${\Delta H}_{corr}$ is H-correction, *i.e.*, the reaction enthalpy change from 0 to 298 K, and $T\Delta S$ is the entropy change between adsorbed hydrogen and hydrogen in the gas phase under standard conditions, based on the Debye model. $H_{corr}=\int_{0}^{298} C_{V}\mathrm{dT}$ was calculated from the vibrational heat capacity using the calculated vibrational frequencies by Harmonic Approximation.^S8^ The H_2_ molecule is treated as an ideal gas, while the adsorbed H is treated using the harmonic approximation. VASP calculations were used to determine the vibrational frequencies of adsorbed H atom on the system. Following **Table E2** gives each contribution to the free energy of the H_2_ molecule.

**Table E2.** Zero-point energy correction (ΔE_ZPE_), enthalpy correction ($\int_{0}^{298} C_{V}dT$), and entropy correction (TΔS) for gaseous H_2_ molecule at the partial pressure of 1. All values are given in eV.

| Species | Δ$E_{ZPE}$ | $\int_{0}^{298} C_{V}dT$ | TΔS |
| --- | --- | --- | --- |
| H_2_ | 0.27 | 0.060 | 0.40 |

**Statistical Analysis**

All statistical analyses, unless stated, were performed with Origin program (Version 2018). The EDX data was evaluated in 3 identical locations of the samples, and averaged. FFT images from TEM were generated using Digital Micrograph (Gatan). The size of nanosheets (or nanoparticles) and the number of layers were measured directly from TEM images with statistical analysis performed on over 50 ones to calculate the mean value and standard deviation (SD). XPS spectra were processed using CASA XPS program. Peak deconvolution was performed by Shirley background subtraction and Voigt functional peak fitting. Electrochemical measurements were repeated three or more times to present mean ± SD from an average of measurements of at least 3 samples. DFT calculations were executed using VASP (Version 5.4.4).

**Table S1**. Composition (mole fraction and mole ratio) of (a) 600A and (b) 400A and UA samples determined using EDX data. The mole fraction of Ru, Mo, V and Nb precursors is defined as mole fraction of corresponding precursor (RuCl_3_, MoCl_5_, VCl_3_, and NbCl_5_). The Se/metal ratio corresponds to the Se vacancies (V_Se_ in %) calculated using the definition as (1 – ½ [Se]/([Ru]+[Mo]+[V] +[Nb]). The data presents mean ± SD from an average of measurements of at least 3 samples, repeating three or more times. The composition of the samples is well correlated with that of the precursors, as shown in the graphs. The Se vacancies *vs. x*_Ru_ is plotted in the last graph.

(a) (RuMoVNb)Se_2_-600A

| No. | Precursors | | | | Products | | | | | |
| --- | --- | --- | --- | --- | --- | --- | --- | --- | --- | --- |
|  | RuCl_3_ MoCl_5_ VCl_3_ NbCl_5_ | | | | Ru Mo V Nb | | | | Se/M | V_Se_ (%) |
| 1 | 0 | 0.33 | 0.33 | 0.33 | 0 | 0.36 | 0.32 | 0.32 | 1.89 | 5.5±0.3 |
| 2 | 0.05 | 0.32 | 0.32 | 0.31 | 0.05 | 0.35 | 0.31 | 0.29 | 1.84 | 8.0±0.4 |
| 3 | 0.10 | 0.30 | 0.30 | 0.30 | 0.10 | 0.30 | 0.31 | 0.29 | 1.84 | 8.0±0.4 |
| 4 | 0.14 | 0.29 | 0.29 | 0.28 | 0.14 | 0.28 | 0.30 | 0.28 | 1.80 | 10.0±0.5 |
| 55 | 0.25 | 0.25 | 0.25 | 0.25 | 0.23 | 0.27 | 0.27 | 0.23 | 1.85 | 7.5±0.4 |
| 6 | 0.40 | 0.20 | 0.20 | 0.20 | 0.35 | 0.18 | 0.22 | 0.25 | 1.80 | 10.0±0.5 |
| 7 | 0.40 | 0.40 | 0.10 | 0.10 | 0.35 | 0.42 | 0.14 | 0.09 | 1.87 | 6.5±0.3 |
| 8 | 0.7 | 0.10 | 0.10 | 0.10 | 0.68 | 0.13 | 0.10 | 0.09 | 1.89 | 5.5±0.3 |
| 9 | 1 | 0 | 0 | 0 | 1 | 0 | 0 | 0 | 2.01 | 0.0±0.2 |

(b) (RuMoVNb)Se_2_-400A and -UA

| No. | 400A | | | | | |
| --- | --- | --- | --- | --- | --- | --- |
|  | Ru Mo V Nb | | | | Se/M | V_Se_ (%) |
| 1 | 0 | 0.36 | 0.32 | 0.32 | 1.88 | 6.0±0.3 |
| 2 | 0.05 | 0.35 | 0.31 | 0.29 | 1.84 | 8.0±0.4 |
| 3 | 0.10 | 0.30 | 0.31 | 0.29 | 1.84 | 8.0±0.3 |
| 4 | 0.13 | 0.26 | 0.31 | 0.30 | 1.80 | 10.0±0.3 |
| 5 | 0.23 | 0.26 | 0.27 | 0.24 | 1.83 | 7.5±0.3 |
| 6 | 0.35 | 0.18 | 0.22 | 0.25 | 1.80 | 10±0.3 |
| 7 | 0.35 | 0.42 | 0.14 | 0.09 | 1.89 | 5.5±0.3 |
| 8 | 0.68 | 0.12 | 0.10 | 0.09 | 1.92 | 4.0±0.3 |
| 9 | 1 | 0 | 0 | 0 | 2.17 | 0.0±0.1 |

| No. | UA | | | | | |
| --- | --- | --- | --- | --- | --- | --- |
|  | Ru | Mo | V | Nb | Se/M | V_Se_ (%) |
| 1 | 0 | 0.38 | 0.33 | 0.29 | 1.95 | 2.5±0.1 |
| 2 | 0.05 | 0.35 | 0.31 | 0.29 | 2.0 | 0.0±0.1 |
| 3 | 0.10 | 0.30 | 0.31 | 0.29 | 1.99 | 0.5±0.1 |
| 4 | 0.13 | 0.27 | 0.28 | 0.32 | 1.99 | 0.5±0.1 |
| 5 | 0.24 | 0.27 | 0.25 | 0.24 | 1.98 | 0.0±0.1 |
| 6 | 0.36 | 0.20 | 0.21 | 0.22 | 2.00 | 0.0±0.1 |
| 7 | 0.36 | 0.41 | 0.14 | 0.09 | 1.96 | 2.0±0.1 |
| 8 | 0.70 | 0.32 | 0.09 | 0.99 | 2.07 | 0.0±0.1 |
| 9 | 1 | 0 | 0 | 0 | 2.40 | 0.0±0.1 |


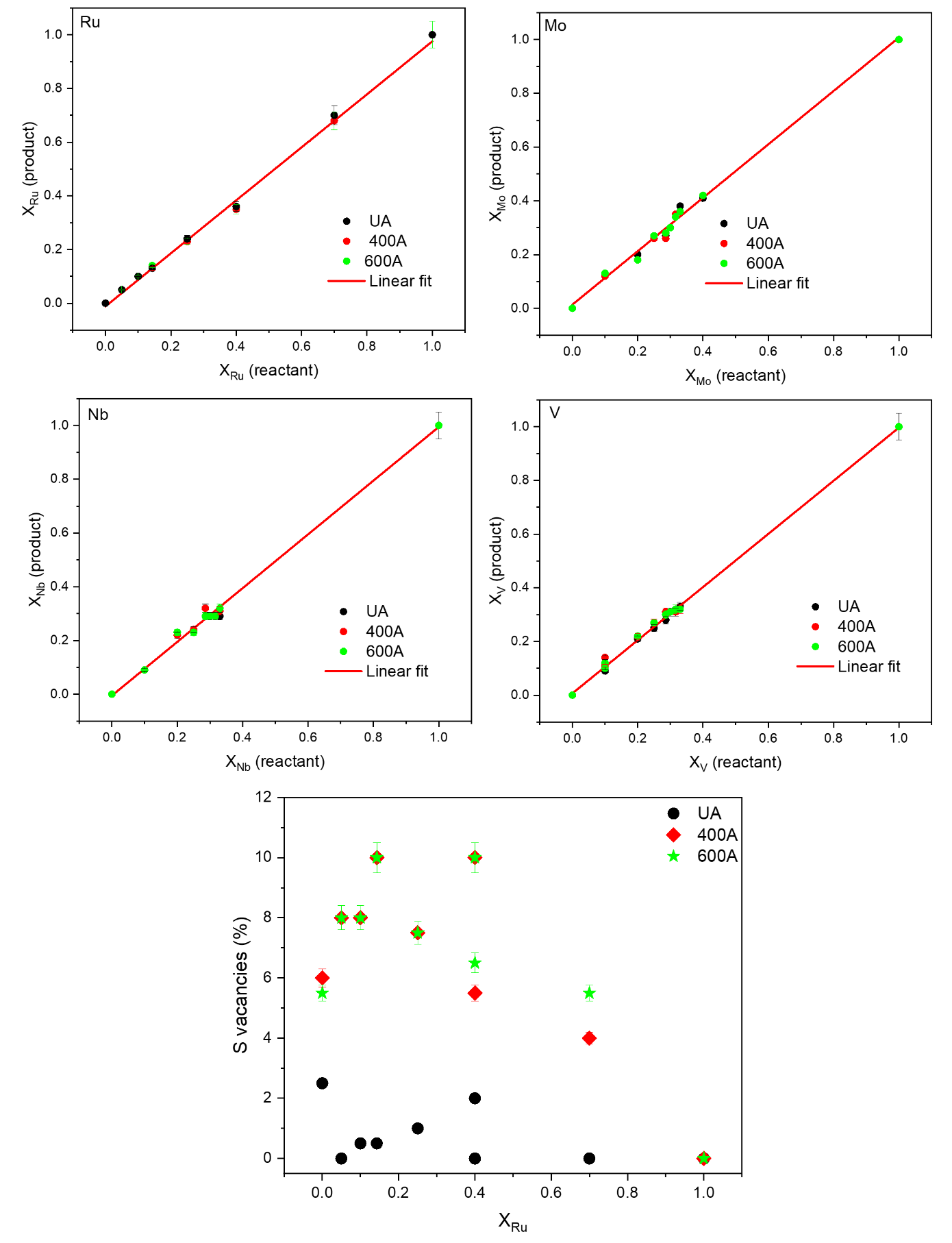


**Table S2**. Fitting parameters of EXAFS data for (a) samples **1**, **6**, and **9** (**Figure 3a** and **Figure S5a**), (b) MoSe_2_, NbSe_2_, VSe_2_, samples **5**, **7**, and **8** (Figure **S5b**), and (c) *in-situ*/*ex situ* samples **6** and **9** (**Figure 5c** and **Figure S5d**); *^a^* Coordination number; *^b^* Distance between scattering atoms; *^c^* Debye-Waller factor, which measures static and thermal disorder. *^d^* Edge energy shift between the energy grids of experimental and theoretical data. *^e^* A percentage misfit between the fitted function and the data.

(a) Samples **1**, **6**, and **9** (600A)

Ru K edge

| Sample No. | Scattering Path | CN*^a^* | R (Å)*^b^* | σ^2^ (10^-3^Å^2^)*^c^* | ΔE_0_ (eV)*^d^* | R factor |
| --- | --- | --- | --- | --- | --- | --- |
| Ru foil | Ru-Ru | 12 | 2.676±0.004 | 4.69±0.85 | 2.87±0.59 | 0.006 |
| 6 | Ru-Se | 4.5±0.5 | 2.493±0.017 | 2.92±0.37 | 5.92±4.32 | 0.016 |
| 9 | Ru-Se | 6.0±0.6 | 2.462±0.008 | 3.32±0.32 | 0.09±2.17 | 0.001 |

Mo K edge

| Sample No. | Scattering Path | CN*^a^* | R (Å)*^b^* | σ^2^ (10^-3^Å^2^)*^c^* | ΔE_0_ (eV)*^d^* | R factor |
| --- | --- | --- | --- | --- | --- | --- |
| Mo foil | Mo-Mo | 8 | 2.722±0.005 | 3.12±0.52 | -5.46±1.00 | 0.006 |
|  | Mo-Mo | 6 | 3.139±0.006 | 2.76±0.66 |  |  |
| 1 | Mo-Se | 5.1±0.3 | 2.533±0.005 | 2.81±0.22 | 0.33±1.76 | 0.002 |
|  | Mo-Metal | 3.0±0.8 | 3.294±0.011 | 6.70±0.87 |  |  |
| 6 | Mo-Se | 3.6±0.5 | 2.509±0.016 | 2.80±0.20 | -1.46±1.25 | 0.014 |
|  | Mo-Metal | 1.7±0.7 | 3.272±0.010 | 6.70±0.82 |  |  |

Nb K edge

| Sample No. | Scattering Path | CN*^a^* | R (Å)*^b^* | σ^2^ (10^-3^Å^2^)*^c^* | ΔE_0_ (eV)*^d^* | R factor |
| --- | --- | --- | --- | --- | --- | --- |
| Nb foil | Nb-Nb | 8 | 2.854±0.004 | 6.64±0.39 | -5.58±0.67 | 0.003 |
|  | Nb-Nb | 6 | 3.286±0.006 | 7.16±0.47 |  |  |
| 1 | Nb-Se | 3.4±0.2 | 2.582±0.005 | 4.69±0.18 | -1.84±0.86 | 0.005 |
|  | Nb-Nb | 2.1±1.1 | 3.321±0.032 | 14.2±2.28 |  |  |
| 6 | Nb-Se | 2.8±0.4 | 2.543±0.023 | 4.73±0.15 | -5.85±1.89 | 0.014 |

Se K edge

| Sample No. | Scattering Path | CN*^a^* | R (Å)*^b^* | σ^2^ (10^-3^Å^2^)*^c^* | ΔE_0_ (eV)*^d^* | R factor |
| --- | --- | --- | --- | --- | --- | --- |
| Se foil | Se-Se | 2 | 2.377±0.004 | 4.52±0.39 | 5.83±1.18 | 0.002 |
| 1 | Se-Mo | 1.4±0.1 | 2.536±0.011 | 2.40±0.61 | 2.27±2.65 | 0.009 |
|  | Se-Nb | 0.8±0.3 | 2.592±0.015 | 2.81±0.74 |  |  |
|  | Se-V | 1.6±0.2 | 2.442±0.003 | 3.02±0.93 |  |  |
|  | Se-Se | 4.3±1.3 | 3.388±0.034 | 7.28±4.15 |  |  |
| 6 | Se-Mo/Nb | 0.8±0.1 | 2.533±0.012 | 2.38±0.69 | 3.90±1.96 | 0.010 |
|  | Se-V | 0.4±0.3 | 2.464±0.017 | 3.02±0.85 |  |  |
|  | Se-Ru | 0.9±0.2 | 2.454±0.004 | 3.42±0.96 |  |  |
|  | Se-Se | 2.6±0.9 | 3.412±0.037 | 7.80±4.20 |  |  |
| 9 | Se-Se (cubic) | 1.0±0.3 | 2.564±0.007 | 2.19±1.06 | 1.23±0.44 | 0.001 |
|  | Se-Ru | 2.0±0.4 | 2.455±0.010 | 3.75±0.59 |  |  |
|  | Se-Se | 2.3±0.4 | 3.350±0.023 | 2.61±1.39 |  |  |

(b) Samples **5, 7**, and **8** (600A)

Se K edge

| Sample No. | Scattering Path | CN*^a^* | R (Å)*^b^* | σ^2^ (10^-3^Å^2^)*^c^* | ΔE_0_ (eV)*^d^* | R factor |
| --- | --- | --- | --- | --- | --- | --- |
| MoSe_2_ bulk | Se-Mo | 3 | 2.536±0.008 | 2.38±0.16 | 5.12±1.43 | 0.006 |
|  | Se-Se | 6 | 3.306±0.005 | 6.30±0.44 |  |  |
| NbSe_2_ bulk | Se-Nb | 3 | 2.591±0.006 | 2.81±0.19 | 2.13±1.84 | 0.004 |
|  | Se-Se | 6 | 3.407±0.014 | 9.66±0.90 |  |  |
| VSe_2_ bulk | Se-V | 3 | 2.468±0.011 | 3.01±0.54 | 4.93±2.65 | 0.015 |
|  | Se-Se | 6 | 3.344±0.024 | 11.1±1.92 |  |  |
| Sample 5 | Se-Mo/Nb | 1.1±0.1 | 2.534±0.011 | 2.37±0.61 | 4.28±2.65 | 0.002 |
|  | Se-V | 0.8±0.3 | 2.466±0.015 | 3.00±0.74 |  |  |
|  | Se-Ru | 0.6±0.2 | 2.454±0.003 | 3.80±0.93 |  |  |
|  | Se-Se | 5.0±1.3 | 3.395±0.034 | 11.5±4.15 |  |  |
| Sample 7 | Se-Mo/Nb | 1.2±0.1 | 2.537±0.010 | 2.37±0.58 | 5.09±1.96 | 0.004 |
|  | Se-V | 0.6±0.3 | 2.469±0.015 | 3.00±0.71 |  |  |
|  | Se-Ru | 0.6±0.2 | 2.454±0.003 | 4.04±0.89 |  |  |
|  | Se-Se | 5.3±1.9 | 3.405±0.036 | 11.3±2.24 |  |  |
| Sample 8 | Se-Mo/Nb | 0.7±0.5 | 2.535±0.010 | 2.37±0.54 | 5.89±1.77 | 0.007 |
|  | Se-Ru | 1.4±0.5 | 2.454±0.023 | 3.40±1.80 |  |  |
|  | Se-Se | 1.4±0.7 | 3.356±0.029 | 4.91±0.89 |  |  |

(c) *In-situ*/*ex-situ* samples **6** and **9** (600A)

Ru K edge

| Sample No. | η (mV) | Scattering Path | CN*^a^* | R (Å)*^b^* | σ^2^ (10^-3^Å^2^)*^c^* | ΔE_0_ (eV)*^d^* | R factor |
| --- | --- | --- | --- | --- | --- | --- | --- |
| 6 | OCV | Ru-Se | 5.9±0.6 | 2.467±0.009 | 2.90±0.33 | -0.26±1.98 | 0.006 |
|  | 0 | Ru-Se | 5.7±0.6 | 2.501±0.017 | 2.90±0.38 | 2.37±2.75 | 0.008 |
|  | 20 | Ru-O | 1.1±0.6 | 1.937±0.011 | 2.86±0.84 | -0.49±3.69 | 0.017 |
|  |  | Ru-Se | 4.8±0.7 | 2.482±0.013 | 2.98±0.54 |  |  |
|  | 100 | Ru-Se | 4.9±0.6 | 2.482±0.011 | 3.12±0.51 | -0.75±2.53 | 0.016 |
| 9 | OCV | Ru-Se | 5.9±0.6 | 2.467±0.009 | 2.90±0.33 | -0.26±1.98 | 0.006 |
|  | 0 | Ru-Se | 5.8±0.6 | 2.473±0.010 | 2.97±0.39 | -0.09±2.25 | 0.013 |
|  | 20 | Ru-Se | 5.6±0.8 | 2.476±0.010 | 2.25±0.41 | 0.13±2.27 | 0.001 |
|  | 40 | Ru-Se | 5.7±1.0 | 2.454±0.016 | 2.87±0.40 | -0.84±2.71 | 0.017 |
|  | 100 | Ru-Se | 6.3±0.6 | 2.464±0.010 | 2.88±0.37 | 0.06±2.11 | 0.002 |

Mo K edge

| Sample No. | η (mV) | Scattering Path | CN*^a^* | R (Å)*^b^* | σ^2^ (10^-3^Å^2^)*^c^* | ΔE_0_ (eV)*^d^* | R factor |
| --- | --- | --- | --- | --- | --- | --- | --- |
| 6 | OCV | Mo-Se | 4.4±0.7 | 2.545±0.016 | 2.78±0.20 | 2.90±2.27 | 0.013 |
|  | 0 | Mo-Se | 3.5±0.5 | 2.534±0.015 | 2.80±0.25 | 2.24±0.64 | 0.007 |
|  | 20 | Mo-Se | 3.3±0.9 | 2.530±0.023 | 2.77±0.20 | 0.04±1.99 | 0.008 |
|  | 100 | Mo-Se | 4.6±1.4 | 2.536±0.014 | 1.39±0.22 | 3.38±2.53 | 0.001 |

Nb K edge

| Sample No. | η (mV) | Scattering Path | CN*^a^* | R (Å)*^b^* | σ^2^ (10^-3^Å^2^)*^c^* | ΔE_0_ (eV)*^d^* | R factor |
| --- | --- | --- | --- | --- | --- | --- | --- |
| 6 | OCV | Nb-Se | 4.0±0.8 | 2.548±0.018 | 4.71±0.13 | -1.84±0.69 | 0.011 |
|  | 100 | Nb-Se | 3.1±0.8 | 2.549±0.018 | 4.72±0.14 | -1.82±0.77 | 0.009 |

**Table S3**. Lattice constant and energy of (5 × 5 × 2) supercells for (Ru_0.28_Mo_0.24_V_0.24_Nb_0.24_)Se*_x_* (*x*_Ru_ = 0.28) and (Ru_0.4_Mo_0.2_V_0.2_Nb_0.2_)Se*_x_* (*x*_Ru_ = 0.4). using (a) pristine (*x* = 2) and (b) Se vacancy (*x* = 1.92) models. E_1T-2H_ corresponds to the energy difference between the most stable configurations of 1T sand 2H phases.

(a)

| *x*_Ru_ | phase | No. | *a* = *b* (Å) | E_rel_ (eV) | E_rel_/atom (meV) | E_1T-2H_ (eV) | E_1T-2H_/atom (meV) |
| --- | --- | --- | --- | --- | --- | --- | --- |
| 0.28 | 2H | 1 | 16.63 | 0 | 0 | 0 | 0 |
|  |  | 2 | 16.59 | 2.403 | 16.019 |  |  |
|  |  | 3 | 16.63 | 3.742 | 24.943 |  |  |
|  |  | 4 | 16.65 | 4.297 | 28.649 |  |  |
|  |  | 5 | 16.65 | 4.524 | 30.161 |  |  |
|  |  | 6 | 16.61 | 5.647 | 37.644 |  |  |
|  |  | 7 | 16.68 | 5.827 | 38.844 |  |  |
|  | 1T | 1 | 16.93 | 0 | 0 | 8.092 | 53.947 |
|  |  | 2 | 16.80 | 0.161 | 1.073 |  |  |
|  |  | 3 | 16.89 | 0.289 | 1.926 |  |  |
|  |  | 4 | 16.89 | 0.458 | 3.054 |  |  |
|  |  | 5 | 16.92 | 0.694 | 4.629 |  |  |
|  |  | 6 | 16.94 | 1.310 | 8.731 |  |  |
|  |  | 7 | 16.87 | 2.067 | 13.781 |  |  |
| 0.4 | 2H | 1 | 16.65 | 0 | 0 | 0 | 0 |
|  |  | 2 | 16.65 | 2.258 | 15.055 |  |  |
|  |  | 3 | 16.60 | 3.209 | 21.390 |  |  |
|  |  | 4 | 16.62 | 3.567 | 23.783 |  |  |
|  |  | 5 | 16.66 | 5.257 | 35.049 |  |  |
|  | 1T | 1 | 16.87 | 0.000 | 0.000 | 3.418 | 22.784 |
|  |  | 2 | 16.84 | 1.387 | 9.246 |  |  |
|  |  | 3 | 16.85 | 1.453 | 9.689 |  |  |
|  |  | 4 | 16.89 | 1.824 | 12.161 |  |  |
|  |  | 5 | 16.90 | 2.834 | 18.896 |  |  |

(b)

| *x*_Ru_ | phase | *a* = *b* (Å) | E_1T-2H_ (eV) | E_1T-2H_/atom (meV) |
| --- | --- | --- | --- | --- |
| 0.28 | 2H | 16.32 | 0 | 0 |
|  | 1T | 16.84 | 1.799 | 12.669 |
| 0.4 | 2H | 16.35 | 0 | 0 |
|  | 1T | 16.80 | 1.139 | 8.021 |

**Table S4**. HER performance of (a) 600A and (b) 400A/UA samples in 1 M KOH, 0.5 M H_2_SO_4_, and 1M PBS electrolytes. The data presents mean ± SD from an average of measurements of at least 3 samples, repeating three or more times. *^a^* Overpotential (mV vs. RHE) at *J* = 10 mA cm^-2^; *^b^* Tafel slope for HER; *^c^* Charge transfer resistance obtained using Nyquist plot of EIS data; *^d^* Double layer capacitance; *^e^* Overpotential (mV vs. RHE) at *J_ECSA_* = 0.05 mA cm^-2^.

(a)

[1 M KOH]

| Sample No. | η_J=10_ (mV)*^a^* | *b* (mV dec^-1^)*^b^* | *R_ct_* (Ω)*^c^* | *C_dl_* (mF cm^-2^)*^d^* | η_JECSA=0.05_ (mV)*^e^* |
| --- | --- | --- | --- | --- | --- |
| 1 | 265 ± 5 | 106 ± 3 | 207.8 ± 4.30 | 24.50 ± 0.81 | 306 ± 6 |
| 2 | 81 ± 3 | 124 ± 2 | 10.48 ± 0.52 | 38.09 ± 1.10 | 150 ± 4 |
| 3 | 60 ± 2 | 115 ± 2 | 7.10 ± 0.31 | 39.07 ± 1.21 | 117 ± 3 |
| 4 | 40 ± 2 | 72 ± 2 | 5.70 ± 0.23 | 45.19 ± 1.40 | 111± 2 |
| 5 | 54 ± 3 | 70 ± 2 | 6.36 ± 0.23 | 40.00 ± 1.22 | 105 ± 4 |
| 6 | 36 ± 2 | 61 ± 1 | 5.18 ± 0.12 | 64.96 ± 1.93 | 83 ± 2 |
| 7 | 37 ± 2 | 64 ± 1 | 5.19 ± 0.11 | 46.65 ± 2.02 | 86 ± 3 |
| 8 | 53 ± 3 | 64 ± 2 | 5.28 ± 0.15 | 42.20 ± 1.34 | 107 ± 3 |
| 9 | 38 ± 2 | 60 ± 1 | 4.01 ± 0.16 | 43.60 ± 1.43 | 80 ± 2 |

[0.5 M H_2_SO_4_]

| Sample No. | η_J=10_ (mV)*^a^* | *b* (mV dec^-1^)*^b^* | *R_ct_* (Ω)*^c^* | *C_dl_* (mF cm^-2^)*^d^* | η_JECSA=0.05_ (mV)*^e^* |
| --- | --- | --- | --- | --- | --- |
| 1 | 137 ± 3 | 54 ± 2 | 11.98 ± 0.43 | 18.85 ± 0.63 | 147 ± 3 |
| 2 | 95 ± 3 | 57 ± 2 | 6.11 ± 0.33 | 13.72 ± 0.42 | 99 ± 2 |
| 3 | 85 ± 2 | 54 ± 1 | 4.54 ± 0.21 | 18.34 ± 0.33 | 94 ± 2 |
| 4 | 86 ± 2 | 51 ± 1 | 4.08 ± 0.10 | 22.68 ± 0.77 | 99 ± 2 |
| 5 | 92 ± 3 | 58 ± 1 | 7.75 ± 0.25 | 13.61 ± 0.63 | 102 ± 1 |
| 6 | 88 ± 2 | 62 ± 2 | 5.72 ± 0.13 | 20.43 ± 0.44 | 102 ± 2 |
| 7 | 87 ± 2 | 71 ± 1 | 6.19 ± 0.15 | 17.22 ± 0.58 | 91 ± 2 |
| 8 | 86 ± 3 | 67 ± 1 | 6.74 ± 0.15 | 15.67 ± 0.55 | 98 ± 2 |
| 9 | 68 ± 2 | 73 ± 1 | 7.41 ± 0.23 | 16.62 ± 0.42 | 80 ± 2 |

[1 M PBS]

| Sample No. | η_J=10_ (mV)*^a^* | *b* (mV dec^-1^)*^b^* | *R_ct_* (Ω)*^c^* | *C_dl_* (mF cm^-2^)*^d^* | η_JECSA=0.05_ (mV)*^e^* |
| --- | --- | --- | --- | --- | --- |
| 1 | 503± 5 | 288 ± 3 | 44.70 ± 2.21 | 3.90 ± 0.29 | 433 ± 6 |
| 2 | 316± 5 | 260 ± 3 | 24.34 ± 1.25 | 17.69 ± 0.93 | 383 ± 4 |
| 3 | 250± 4 | 215 ± 2 | 22.80 ± 1.13 | 19.92 ± 0.64 | 345 ± 4 |
| 4 | 227± 4 | 164 ± 2 | 21.41 ± 1.14 | 23.56 ± 1.25 | 318 ± 3 |
| 5 | 166± 3 | 145 ± 1 | 20.30 ± 1.08 | 28.25 ± 1.43 | 313 ± 2 |
| 6 | 139± 3 | 124 ± 1 | 17.70 ± 0.95 | 33.05 ± 1.72 | 258 ± 3 |
| 7 | 196± 5 | 163 ± 2 | 22.70 ± 1.04 | 25.19 ± 1.31 | 323 ± 3 |
| 8 | 163± 4 | 143 ± 2 | 19.91 ± 1.17 | 26.94 ± 1.35 | 321 ± 2 |
| 9 | 146± 3 | 90 ± 1 | 18.01 ± 0.83 | 29.89 ± 1.26 | 299 ± 2 |

(b)

[1 M KOH]

| Sample No. | η_J=10_ (mV)*^a^* | *b* (mV dec^-1^)*^b^* | *R_ct_* (Ω)*^c^* | *C_dl_* (mF cm^-2^)*^d^* | η_JECSA=0.05_ (mV)*^e^* |
| --- | --- | --- | --- | --- | --- |
| 1-400A | 265 ± 5 | 88 ± 2 | 203.0 ± 4.0 | 20.09 ± 0.73 | 292 ± 5 |
| 6-400A | 38 ± 2 | 53 ± 1 | 4.86 ± 0.15 | 41.60 ± 1.23 | 79 ± 2 |
| 9-400A | 54 ± 3 | 79 ± 2 | 4.77 ± 0.15 | 42.85 ± 1.35 | 102 ± 3 |
| 6-UA | 40 ± 2 | 73 ± 1 | 5.41 ± 0.23 | 42.60 ± 1.33 | 80 ± 2 |
| 9-UA | 49 ± 3 | 70 ± 1 | 4.99 ± 0.16 | 32.39 ± 1.07 | 86 ± 3 |

[0.5 M H_2_SO_4_]

| Sample No. | η_J=10_ (mV)*^a^* | *b* (mV dec^-1^)*^b^* | *R_ct_* (Ω)*^c^* | *C_dl_* (mF cm^-2^)*^d^* | η_JECSA=0.05_ (mV)*^e^* |
| --- | --- | --- | --- | --- | --- |
| 1-400A | 133 ± 3 | 71 ± 2 | 7.99 ± 0.30 | 16.79 ± 0.57 | 140 ± 3 |
| 6-400A | 104 ± 3 | 66 ± 2 | 8.04 ± 0.30 | 16.23 ± 0.48 | 137 ± 3 |
| 9-400A | 81 ± 2 | 50 ± 1 | 7.66 ± 0.25 | 12.30 ± 0.43 | 82 ± 2 |
| 6-UA | 107 ± 2 | 81 ± 2 | 7.79 ± 0.25 | 15.16 ± 0.54 | 127 ± 3 |
| 9-UA | 91 ± 2 | 67 ± 2 | 7.31 ± 0.20 | 7.16 ± 0.36 | 80 ± 2 |

**Table S5**. Comparison of HER performance (in 0.5 M H_2_SO_4_ and 1 M KOH electrolyte of RuX_2_-based materials in the literatures. The “temperature” is referred to as the annealing temperature or the temperature of chemical vapor deposition (CVD) or selenization/sulphuration. The notation “*a*” was used for amorphous phase, “c” for cubic phase, “h” for hexagonal phase.

| Ref. No. | Materials | Working electrode | 1 M KOH | | 0.5 M H_2_SO_4_ | |
| --- | --- | --- | --- | --- | --- | --- |
|  |  |  | η_J=10_ (mV) | Tafel slope (mV dec^-1^) | η_J=10_ (mV) | Tafel slope  (mV dec^-1^) |
| S9 | *a*-RuS*_x_*/S–doped graphene oxide (hydrothermal) | CP (carbon  Paper) | 58 | 56 | 31 | 40 |
| S10 | c-RuS_2_-NiS_2_-carbon-450 ℃ (ball-milling) | GCE (glassy carbon electrode) | 25 | 34 | 43 | 39 |
| S11 | c-Se-rich RuSe_2_-400 ℃ (hydrothermal) | CP | 45 | 31.9 | -- | -- |
| S12 | *h*-RuSe_2_ (colloidal)-400 ℃ | GCE | 34 | 95 | -- | -- |
| S13 | c-RuSe_2_-CNT-700 ℃ (CVD) | GCE | 29.5 | 39.2 | 87.8 | 70.3 |
| S14 | c-RuSe_2_ -600 ℃  c-RuSe_2_-N-doped C (grinding) | -- | 57  30 | 64  32 | -- | -- |
| S15 | c*-*RuS_x_-650 ℃  c-Ru-RuS_2_ (colloidal) | CP | 29  16 | 151  29 | -- | -- |
| S16 | c-RuSe_2_-CNTs-650 ℃  (microwave) | GCE | 48 | 30 | 64 | 42 |
| S17 | c/1T phase-RuSe_2_-500 ℃ (microwave) | GCE | 29 | 64 | -- | -- |
| S18 | c-RuSe_2_-400 ℃  c-RuSe_2_-Co-N–doped C-400 ℃  (hydrothermal) | GCE | 90  26.4 | 77.6  53.2 | -- | -- |
| S19 | Cu-doped Ru/c-RuSe_2_  (solvothermal and selenization at 300 ℃) | GCE | 23 | 58.5 | -- | -- |
| S20 | c-RuSe_2_-400 ℃  c-RuSe_2_-CoTe (hydrothermal) | Ni foam | 65  25.4 | 113.6  67.8 | -- | -- |
| S21 | *a*-RuS_x_  *a*-RuS_x_ on CNTs (solvothermal) | GCE | 137  17 | 149  35 | -- | -- |
| S22 | c-RuSe_2_-400 ℃  c-RuSe_2_/CeO_2_ (hydrothermal) | GCE | 58  16 | 75.4  61.1 | -- | -- |
| S23 | c-RuTe_2_-B/N-doped graphene-500~700 ℃ (freeze-drying and pyrolysis) | GCE | 16 | 57.6 | 34 | 59.8 |
| S24 | *a*-RuSe_1.5_  *a*-RuSe_2_ (solvothermal) | GCE | 12  32 | 25  42 | 24  59 | 34  52 |
| S25 | RuO_2-x_/c-RuSe_2_-C-600 ℃ (electrospinning/selenization) | GCE | 10 | 37.6 | 25 | 32.1 |
| S26 | Ru-doped MoSe_2_ (selenization at 450 ^o^C) | GCE | 147.8 | 109.1 | -- | -- |
| S27 | *a*-RuSe_2_ (colloidal) | GCE | -- | -- | 75 | 47 |
| S28 | c-RuSe_2_-WO_x_-C-650 ℃ (hydrothermal) | GCE | 60 | 97 | 58 | 69 |
| S29 | c-RuSe (room temperature reaction) | GCE | 53 | 79 | 37 | 48 |
| S30 | c-RuSe_2_-500 ℃  c-Mo-doped RuSe_2_ (solvothermal) | GCE | 72  27 | 74  39 | -- | -- |
| This Work | c-RuSe_2_-600 ℃  Ru_0.40_Mo_0.20_V_0.20_Nb_0.20_Se_2_  (colloidal) | GCE | 38  36 | 60  61 | 68  88 | 73  62 |


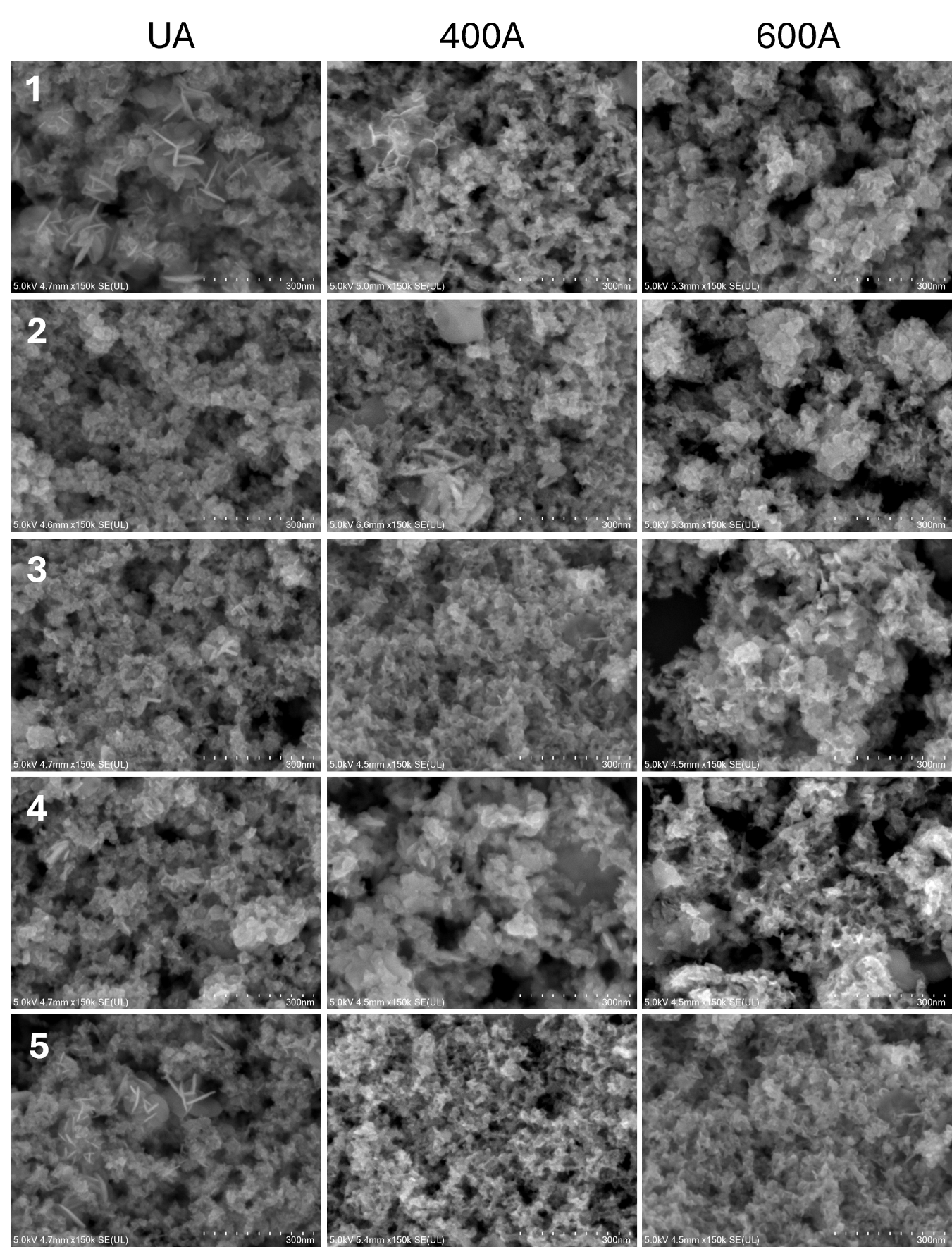


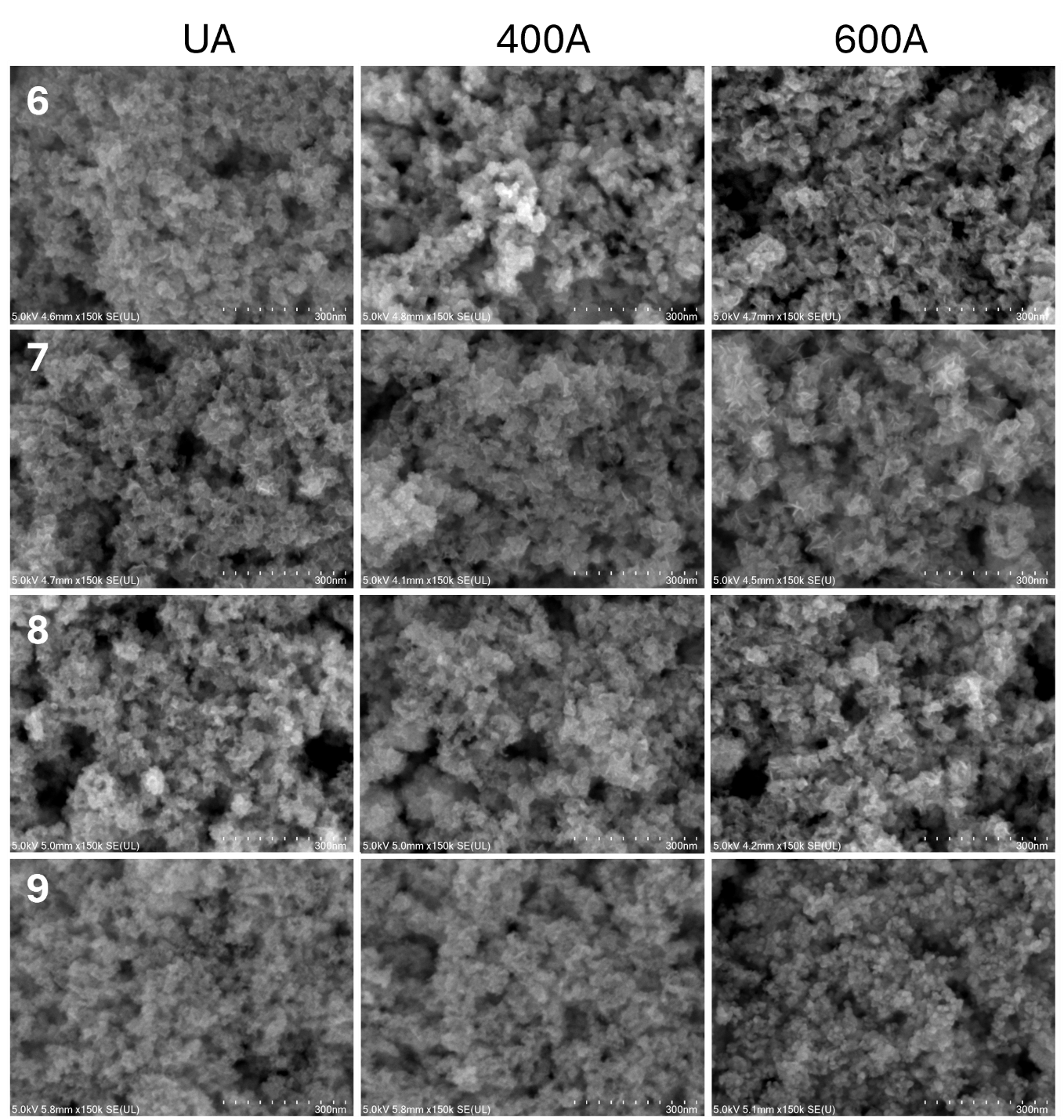


600A
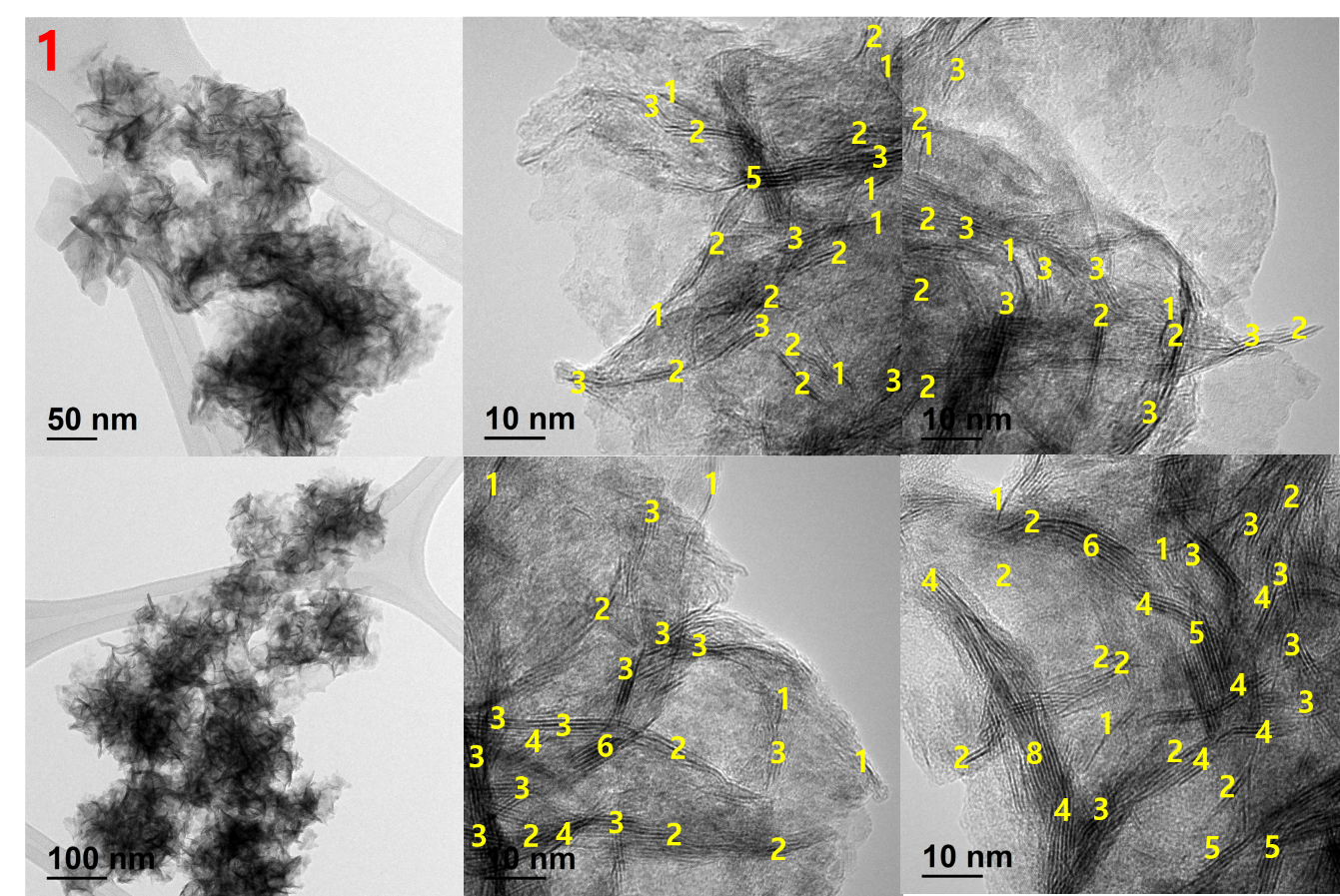


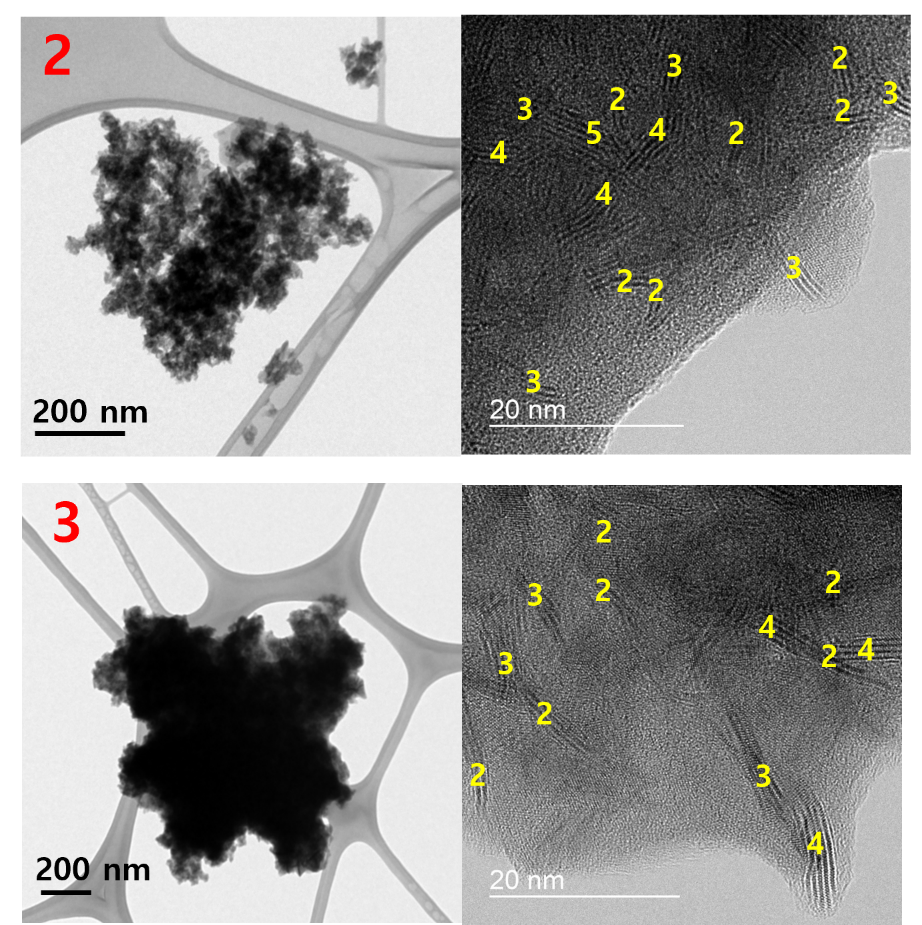


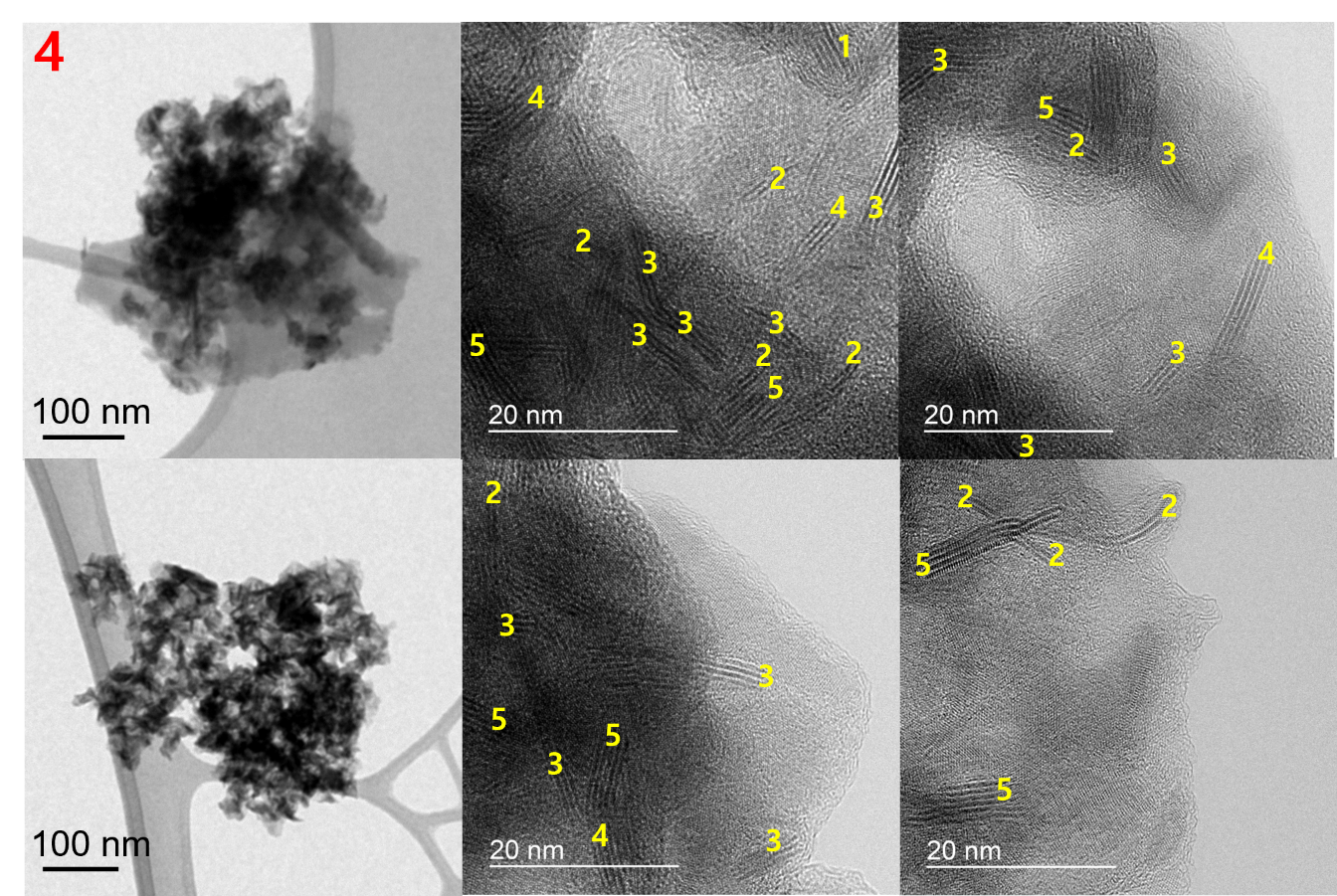


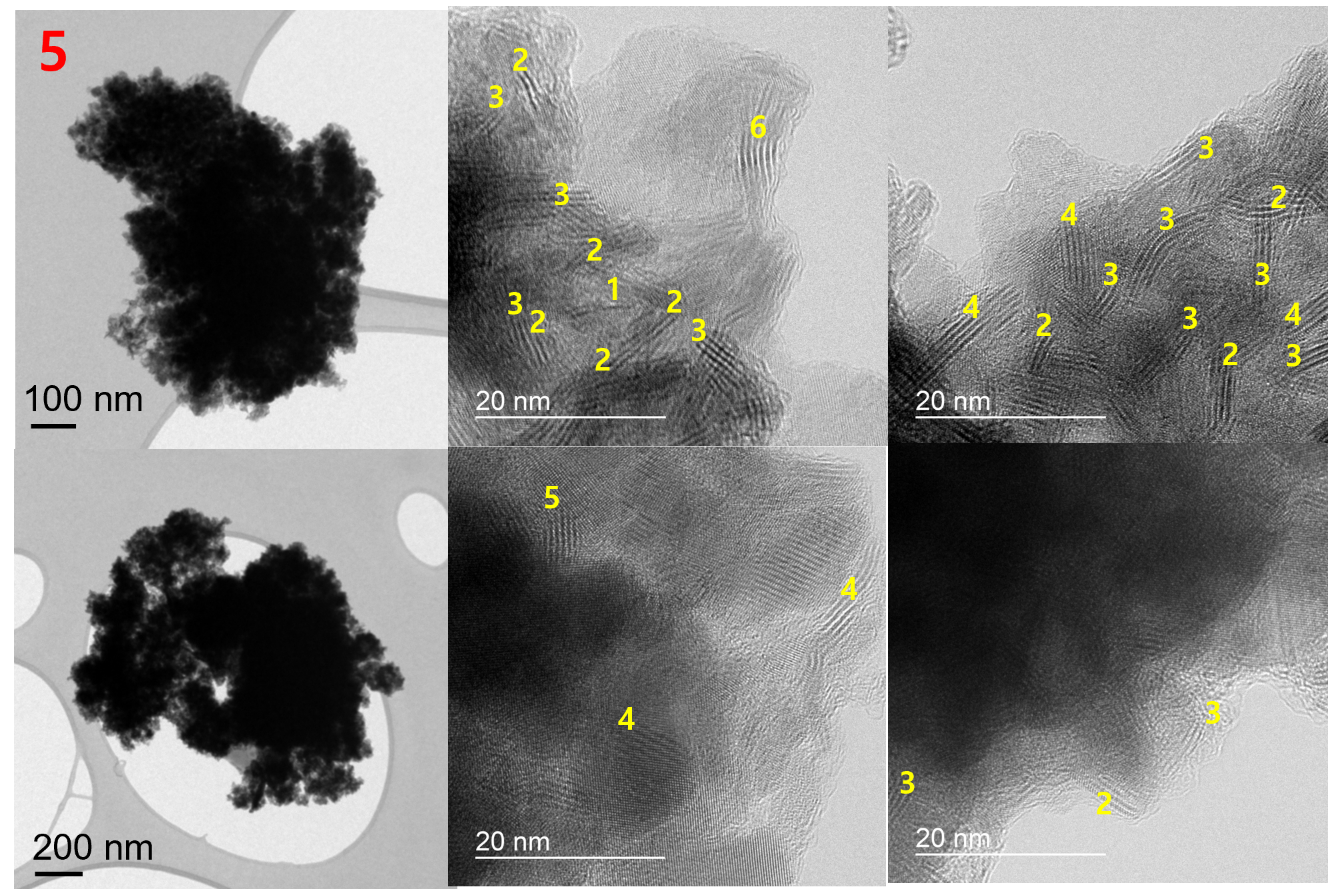


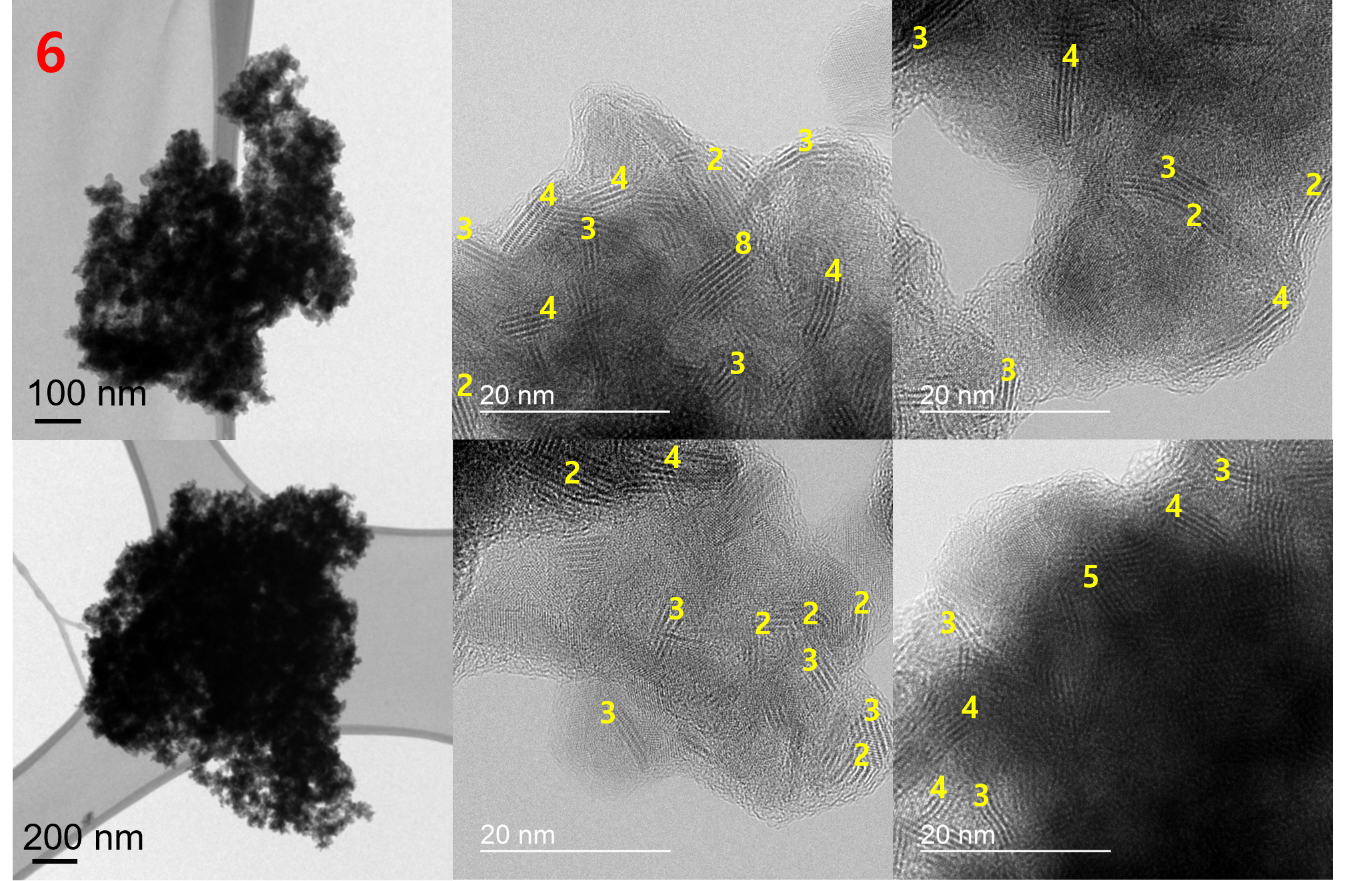


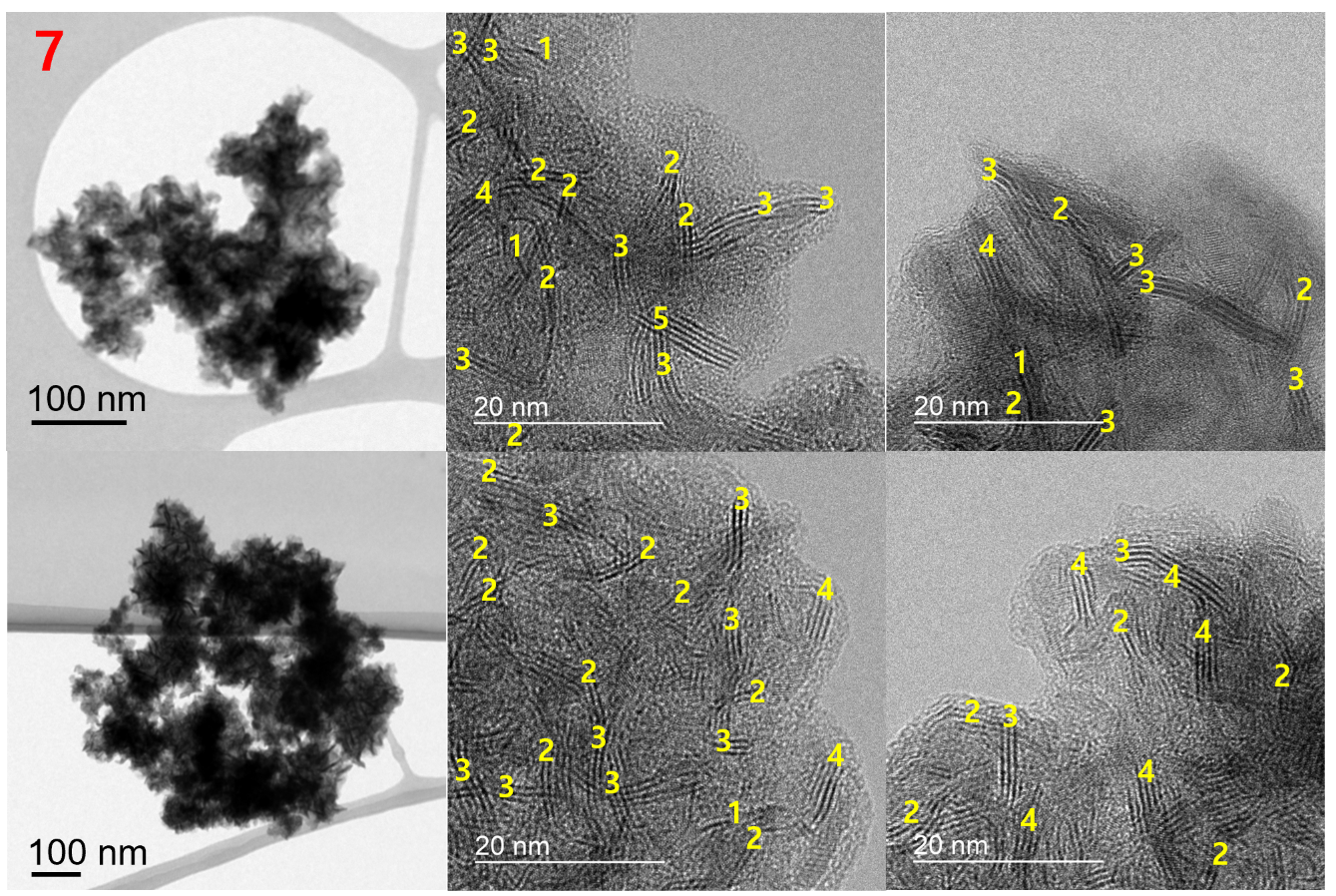


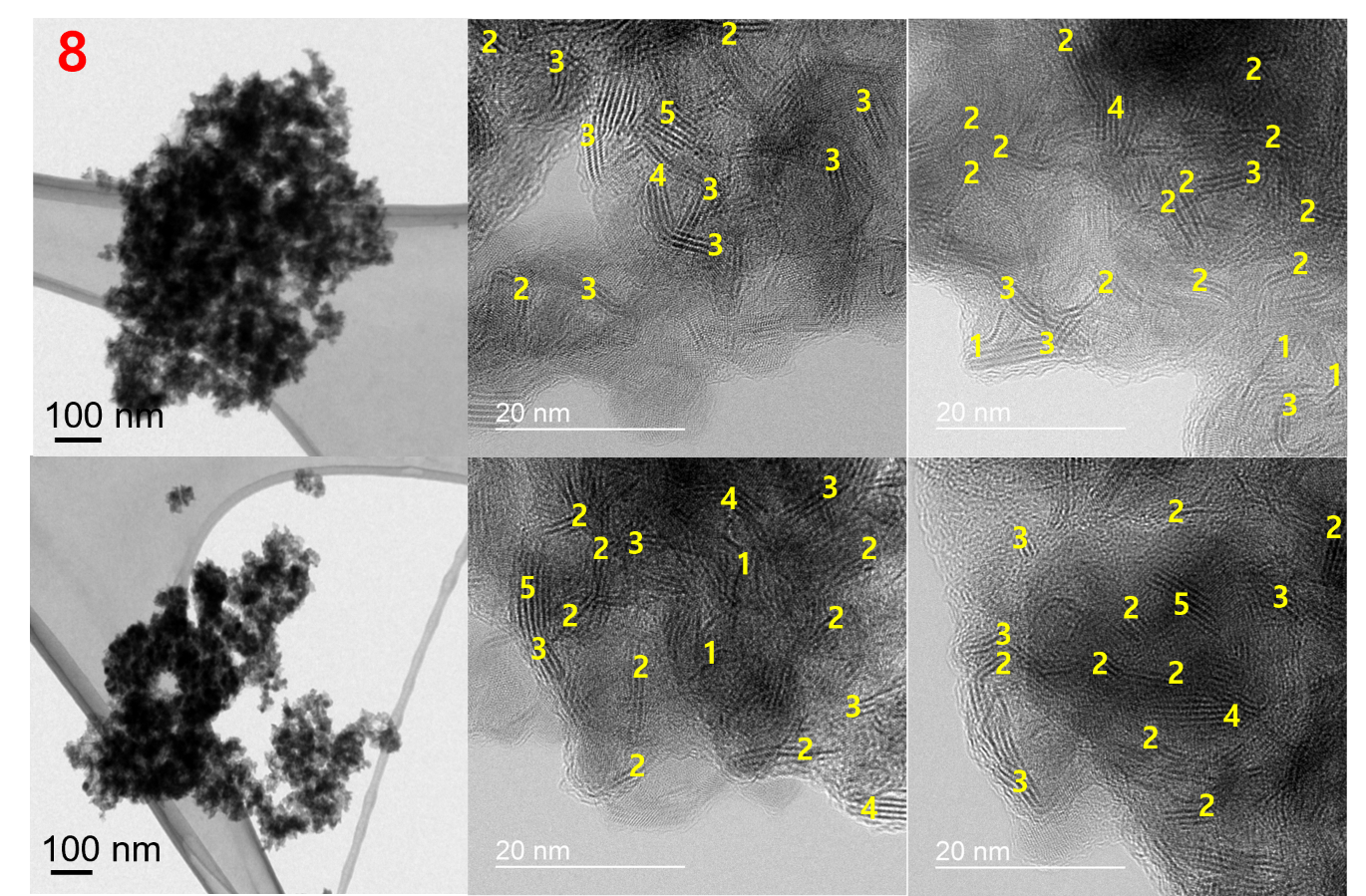


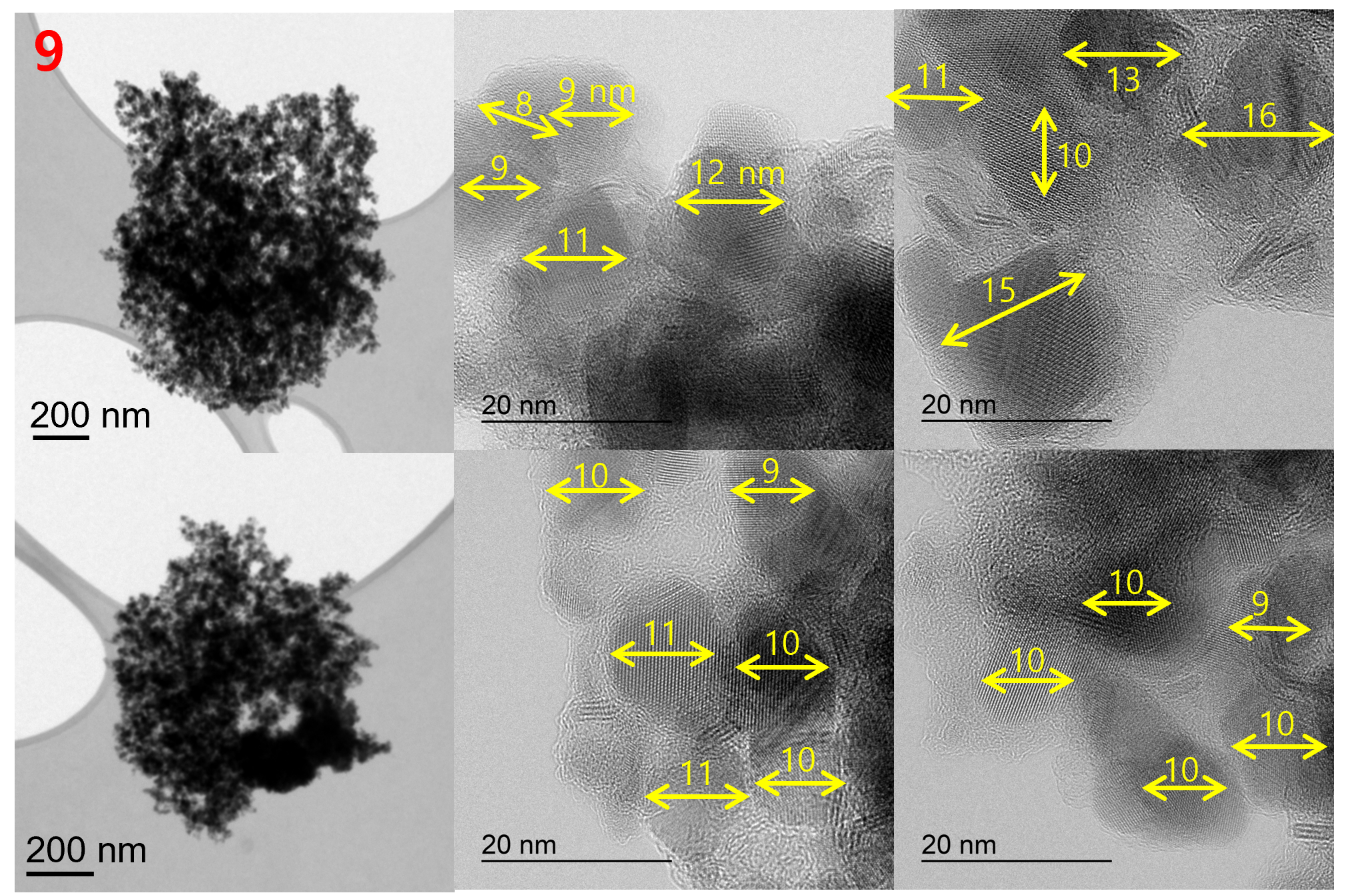


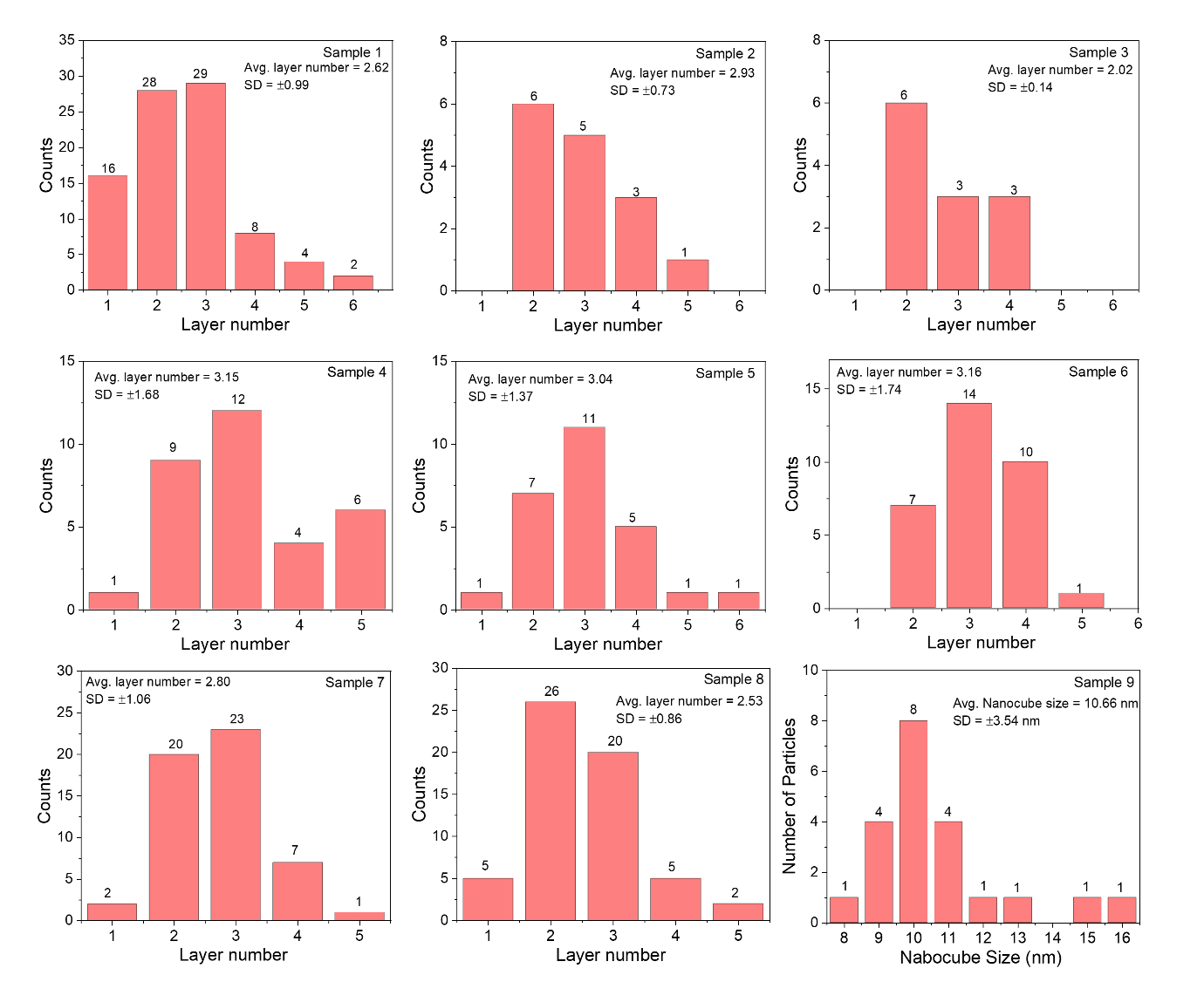


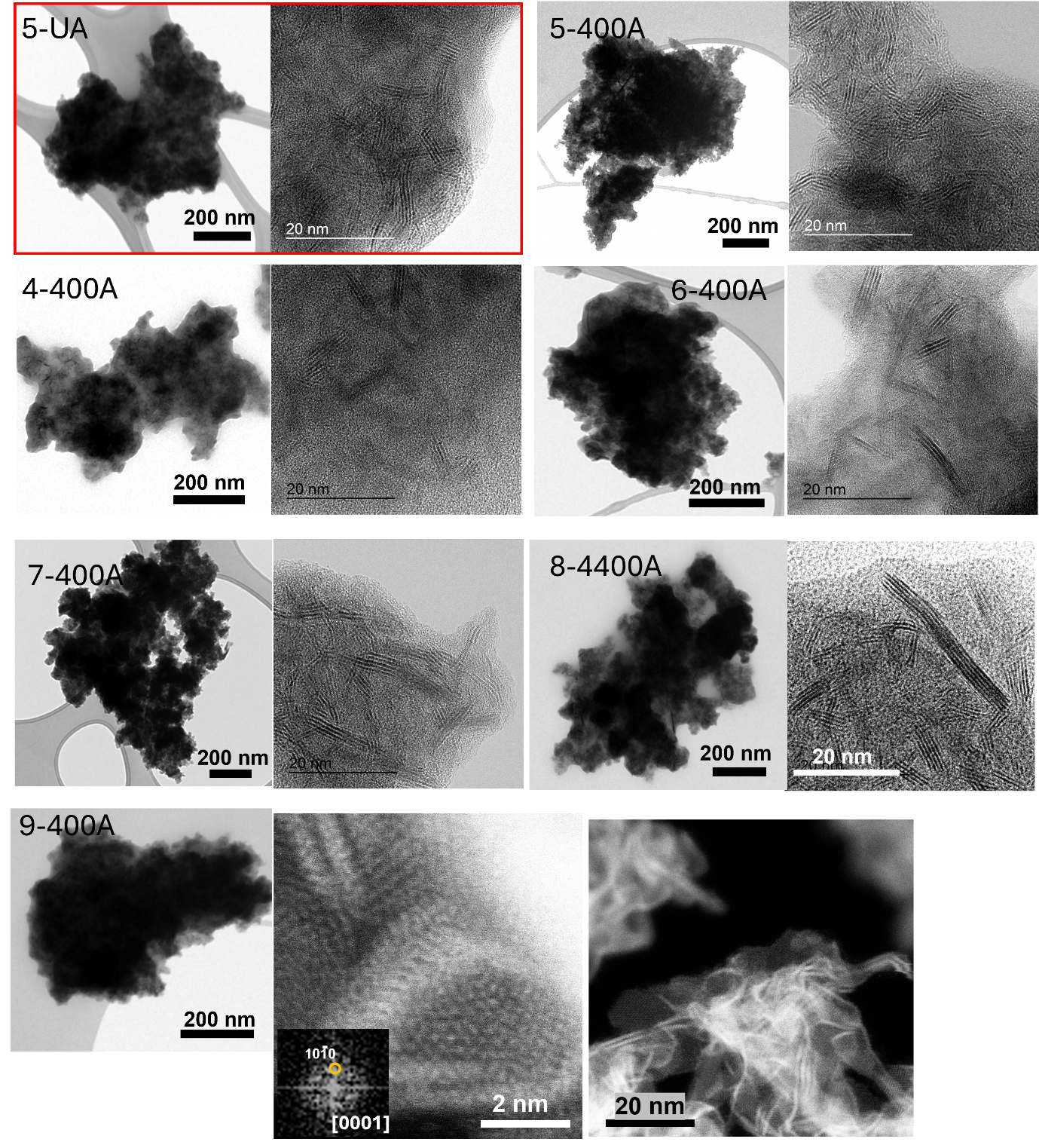


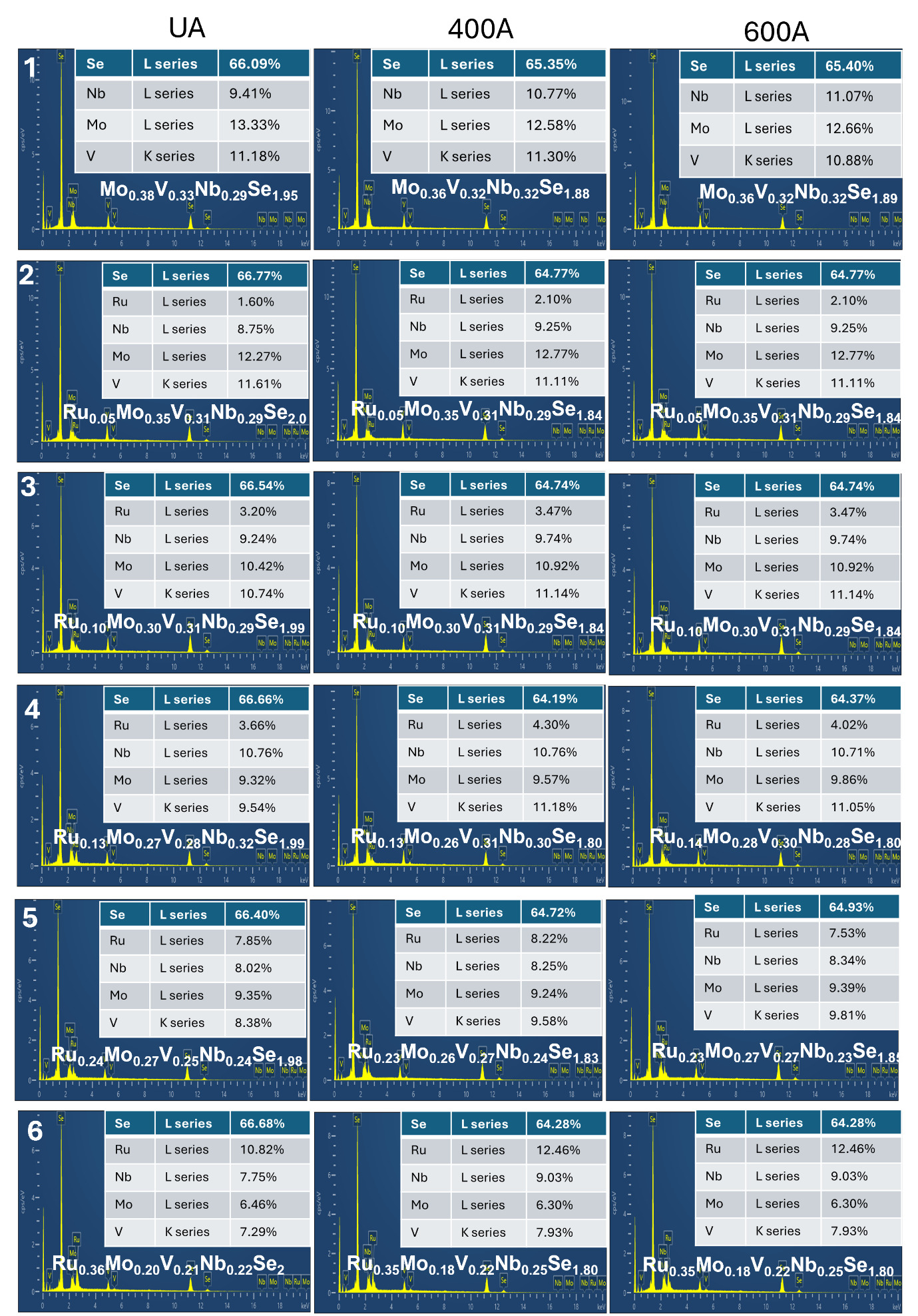

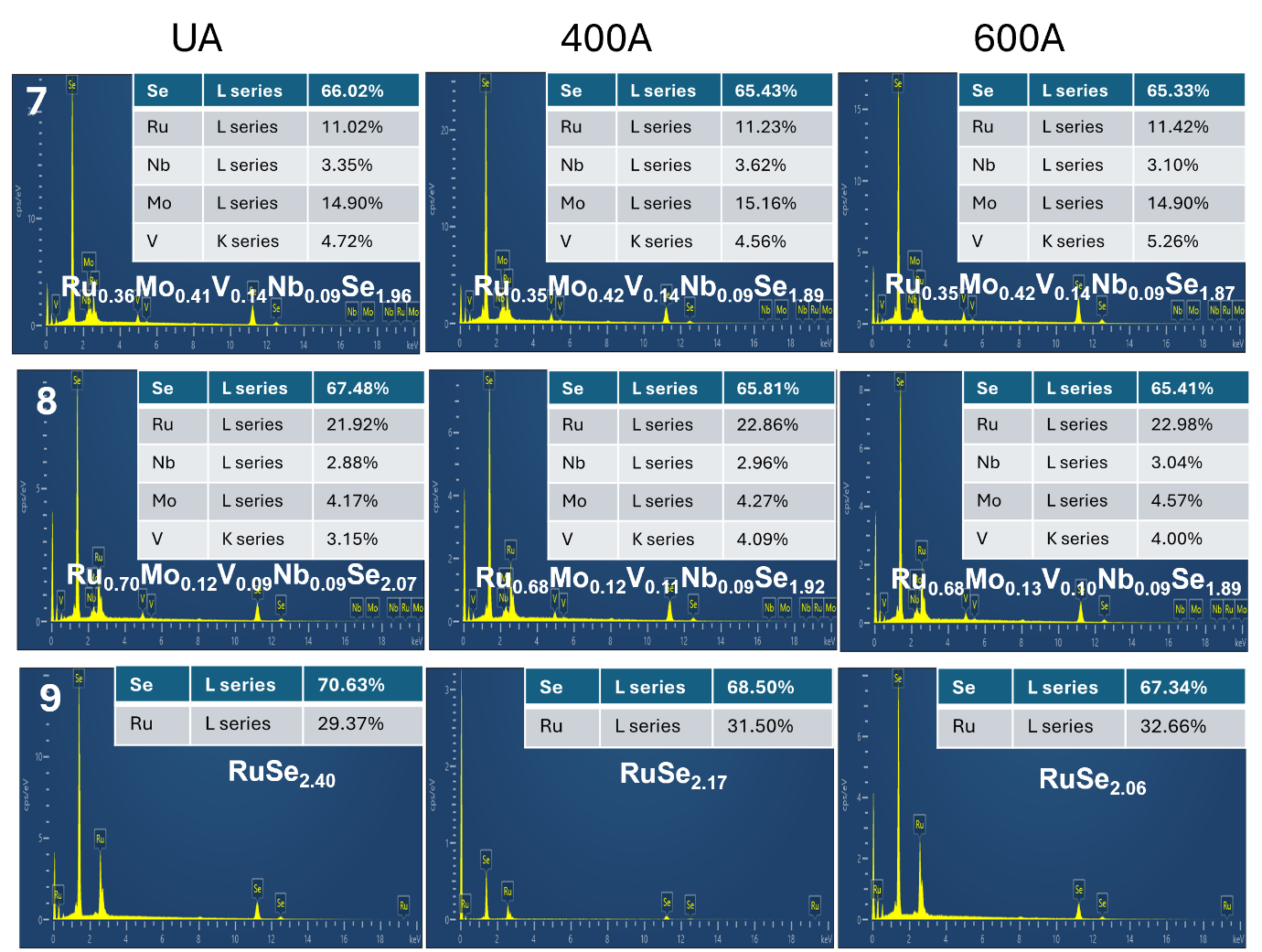
 **Figure S1**. SEM, HRTEM, and EDX data of samples **1**-**9** (600A, 400A, and UA). HAADF STEM images and EDX spectrum. The Ru (L shell), Mo (L shell), V (K shell), Nb (L shell), and Se (L shell) showed that that the composition was successfully controlled.

The SEM and HRTEM images show that the annealing didn’t change the morphology and metal composition (EDX spectrum) of the alloy samples (**1**-**8**). As shown in statistics, the nanosheets comprised *avg.* 3 layers (thickness: ~2 nm) that aggregated into nanoflowers. The interlayer distance (*d*_001_) of nanosheets was 6 Å. The 400A samples of RuSe_2_ (sample **9**) consisted of 1-2 layered nanosheets (*d*_001_ = 6 Å), suggesting the layer structures with 2H or 1T phase. The RuSe_2_-600A sample shows the cube-type nanocrystal because of the cubic phase.

The UA samples show [Se]/[Metal] = 1.95~2.40, indicating 1-2% Se vacancies. The annealing of sample **9** (RuSe_2_) didn’t produce the Se vacancies. In contrast, the annealing increases the Se vacancies (see Table S1). Annealed samples **1**–**7** have *avg.* 8 % Se vacancies (max. 10%), and sample **8** has 4% Se vacancies (see Table S1).

Atomic resolved STEM images reveal the nanosheet morphology of sample **9**-400A. Its corresponding FFT image displays the {010} reflection spots of the 2H phase with *d*_010_ = 2.9 Å.


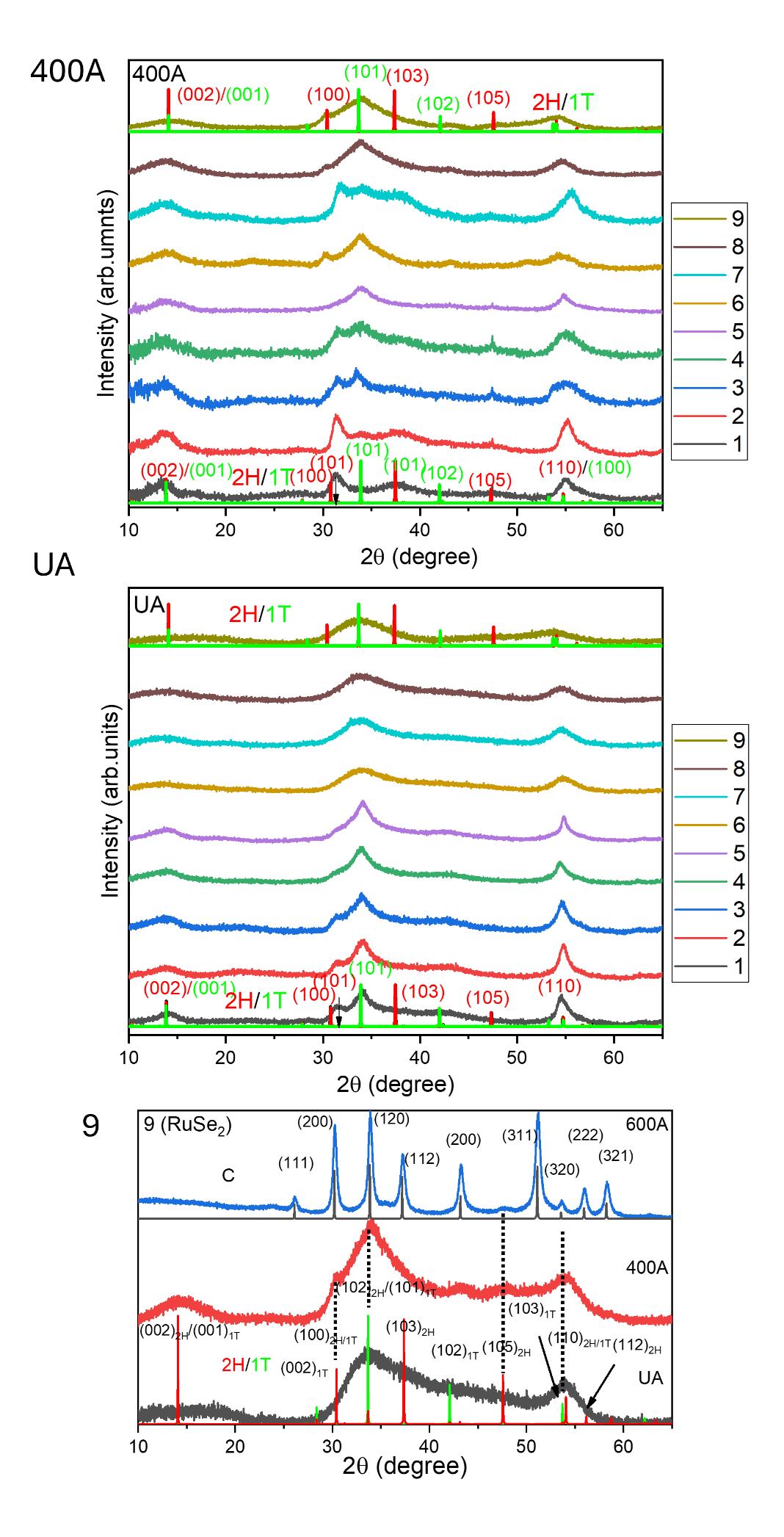


**Figure S2**. XRD data of samples **1**-**9** (400A and UA), together with the reference peaks generated using the lattice constants of 2H/1T MoSe_2_-like phase for sample **1**, and 2H/1T phase RuSe_2_ for sample **9**. XRD patterns of RuSe_2_ sample **9**-UA, **9**-400A, and **9**-600A, with the reference peaks of C, 2H, and 1T phase.

The 400A samples of (MoVNb)Se_2_ ternary alloy nanosheets (sample **1**) show the 2H MoSe_2_-like phase peaks, where their lattice constants are *a*_2H_ = 3.3 Å and *c*_2H_ = 13.0 Å. The XRD peaks of RuSe_2_ (sample **9**) are matched with the lattice constant of 2H or 1T; (*a*_2H_, *c*_2H_) = (3.42, 12.58) Å and (*a*_1T_, *c*_1T_) = (3.42, 6.29) Å. As *x*_Ru_ increases to 0.4 (sample **6**), the XRD pattern becomes closer to that of RuSe_2_.

The **1**-UA sample shows that the 1T peaks coexist with the 2H peaks, where the lattice constants are *a*_1T_ = 3.3 Å and *c*_1T_ = 6.5 Å. The annealing converted the 1T phase into the 2H phase. As *x*_Ru_ increases to 0.4 (sample **6**), the phase becomes closer to the RuSe_2_ phase.

The lower panel shows that the XRD patterns of samples **9**-UA and **9**-400A (RuSe_2_) are distinct from that of **9**-600A. The peaks for the UA and 400A samples can be assigned to either the 2H or 1T phases, with lattice constants *a*_2H/1T_ = 3.42 Å, *c*_2H_ = 12.58 Å, and *c*_1T_ = 6.29 Å. The peak of **9**-600A corresponds to the C phase with a lattice constant *a*_C_ = 5.92 Å, which is consistent with the reference value (*a*_C_ = 5.933 Å, JCPDS No. 65-3328). The peak at 2θ = 45.6° is assigned to (105)_2H_, indicating that the 2H phase remains.


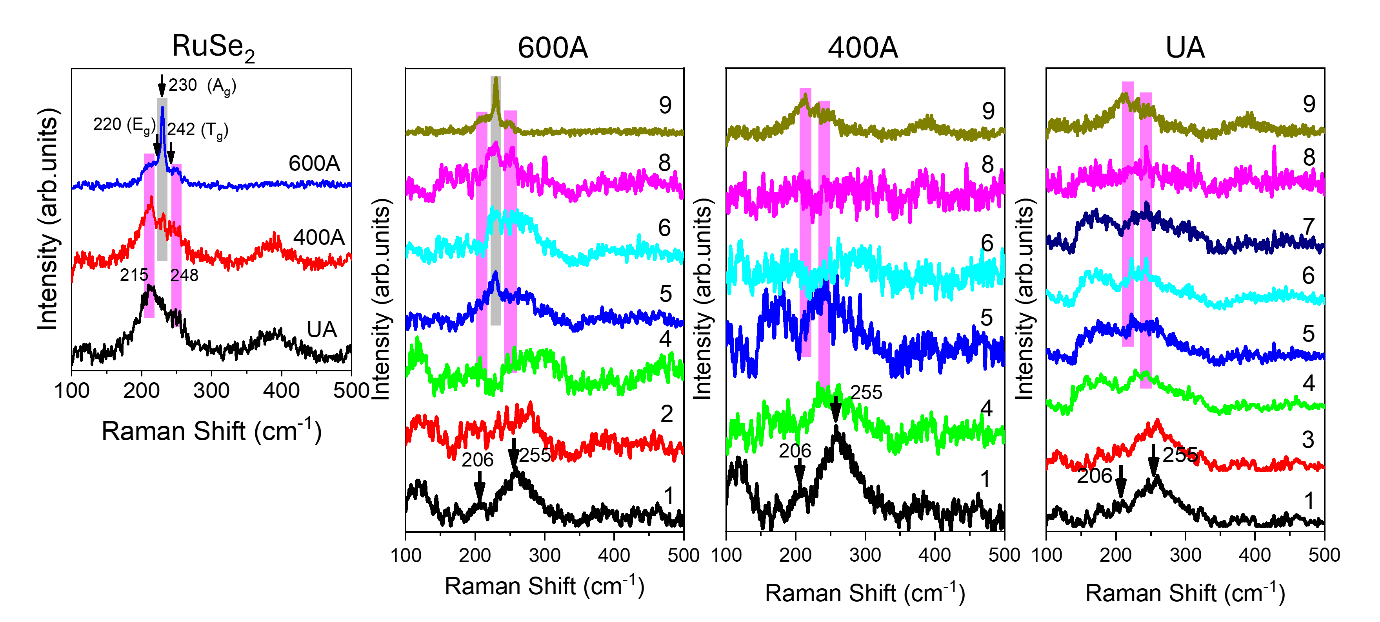


**Figure S3**. Raman spectrum of RuSe_2_ (sample **9**) and 600A/400A/UA samples of **1**~**9**. The excitation source is 532 nm diode laser.

The phase of RuSe_2_ was investigated by Raman spectroscopy. The three peaks are found in the spectrum of 600A sample. The 230 cm^-1^ peak is ascribed to the A_g_ mode of the cubic phase. The E_g_ and T_g_ mode peak should be appeared at 220 and 242 cm^-1^, respectively*.*^S31,S32^ The UA and 400A samples showed the Raman peaks at 215 and 248 cm^−1^, which were assigned to the 1T and 2H phase RuSe_2_, respectively, by two research groups.^S12,S19^ For 600A sample, these two peaks still remains, indicating the 2H/1T phase coexists with the C phase, consistently with the XRD data shown in Figure S3.

The sample **1** shows the peaks at 206 and 255 cm^-1^. In our previous works, the A_g_ mode of 2H phase MoSe_2_, 2H phase NbSe_2_, and 1T phase VSe_2_ located at 244, 243, and 210 cm^-1^, respectively.^S33-S35^ Therefore, the two peaks can be assigned to the A_g_ vibration modes of V-Se and Mo-Se/Nb-Se bonds, respectively. As *x*_Ru_ increases to 0.25 (sample **5**), the intensity of those peaks decreases significantly, while the intensity of RuSe_2_ peak increases.

**(a)**


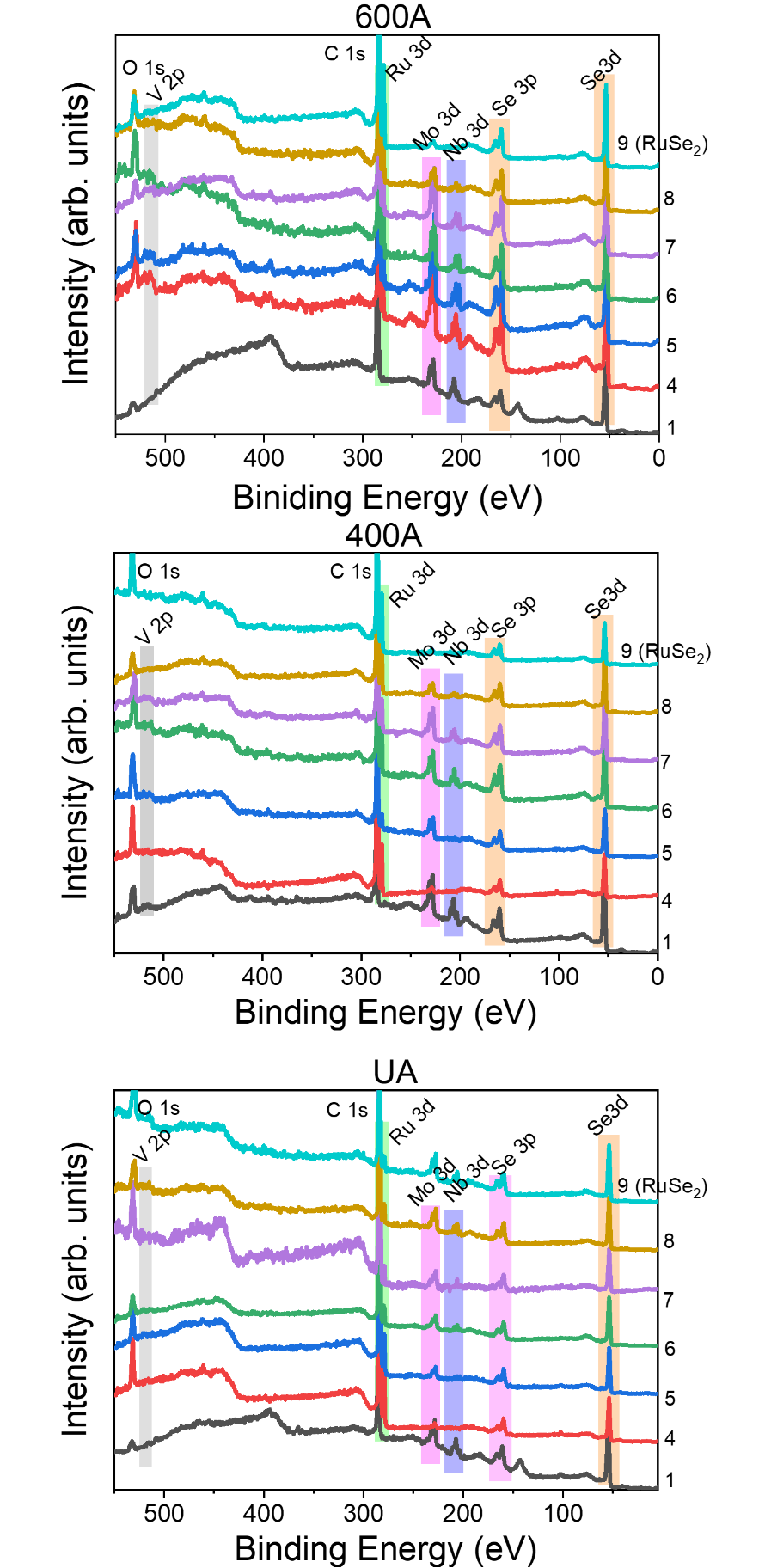


**(b)**


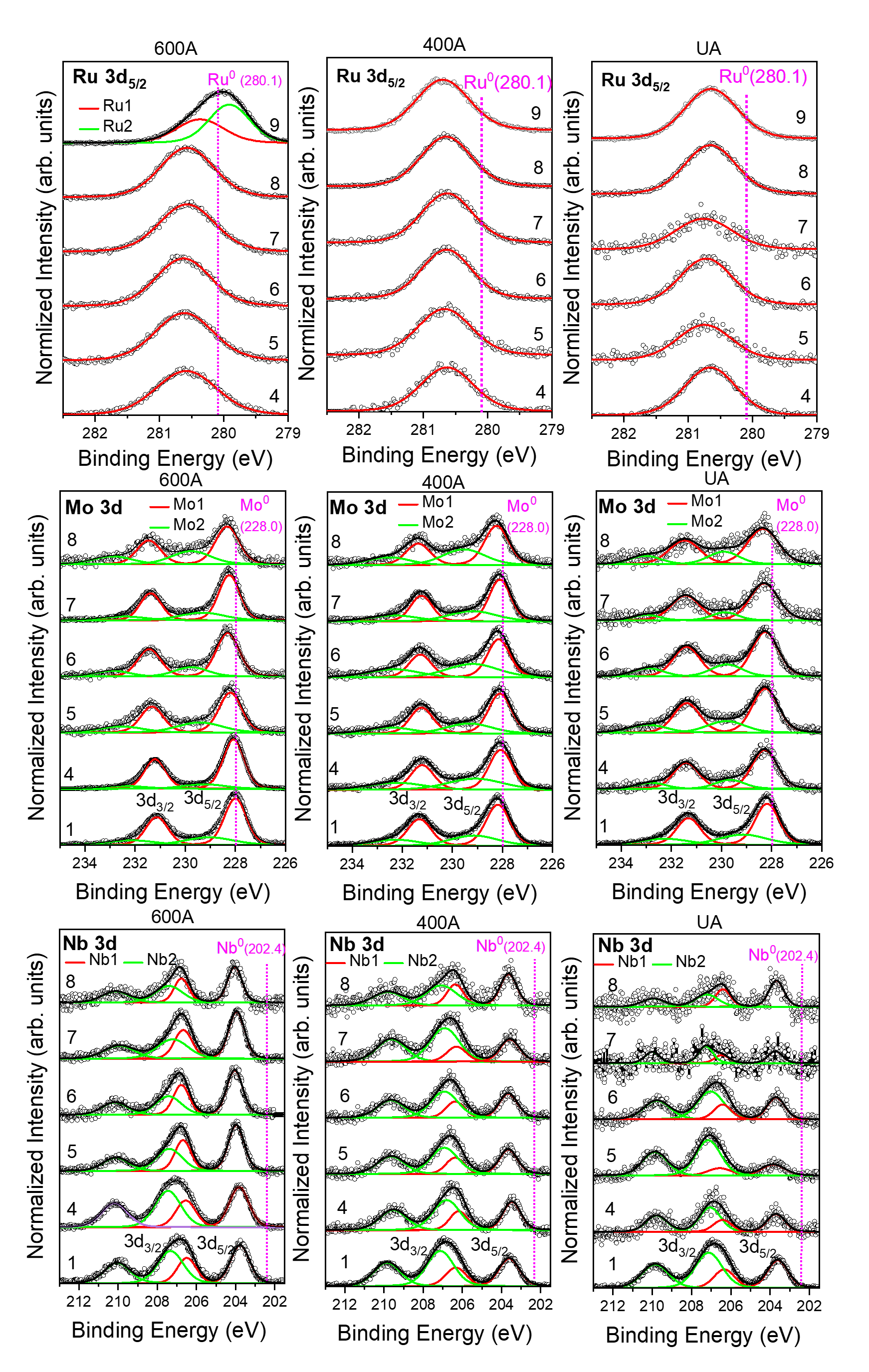


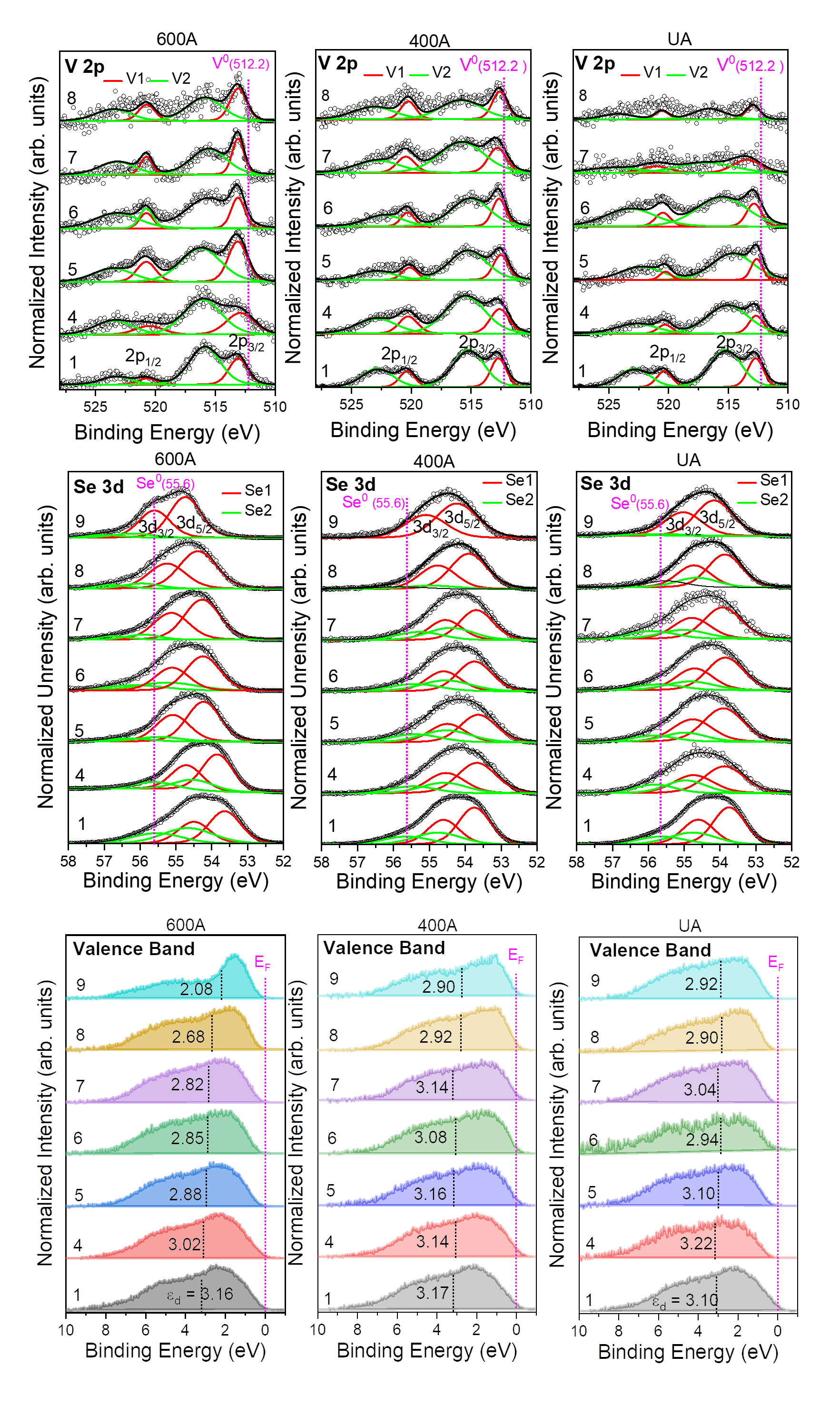


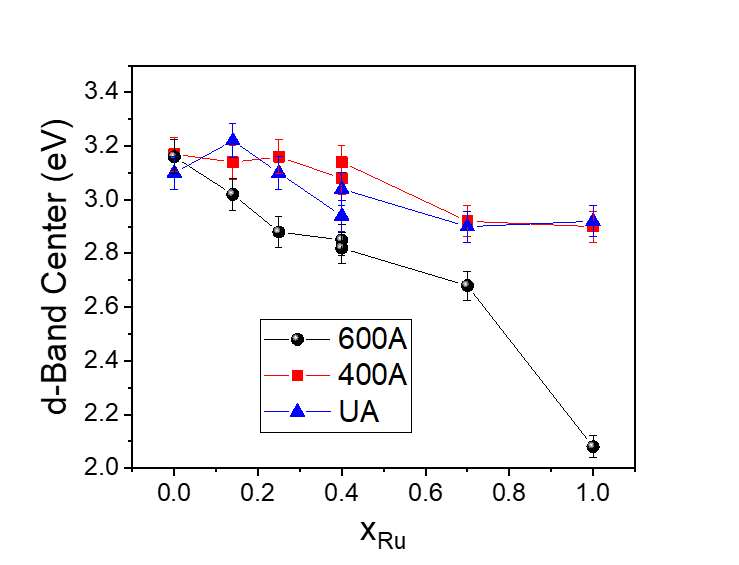


(c)


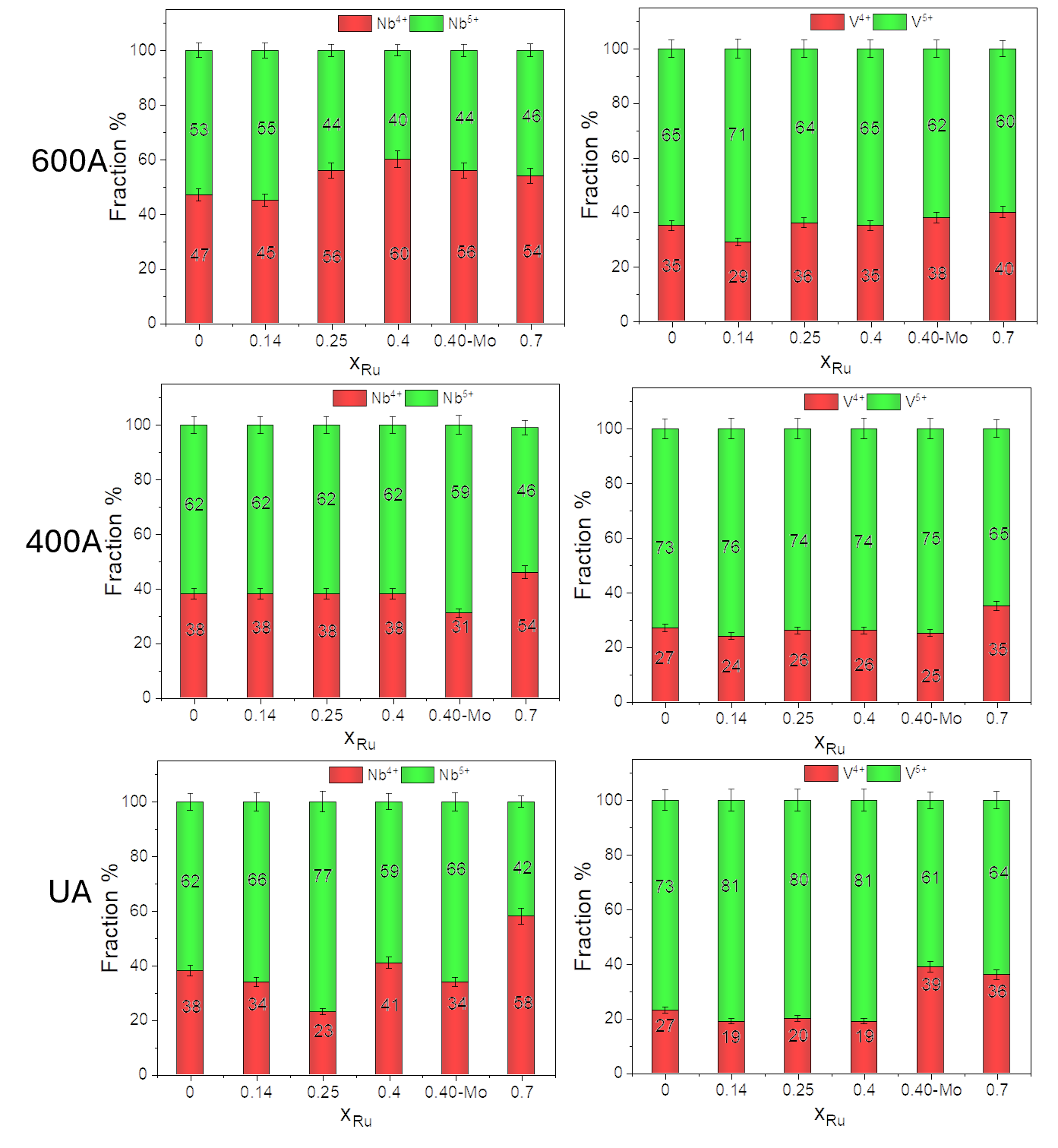


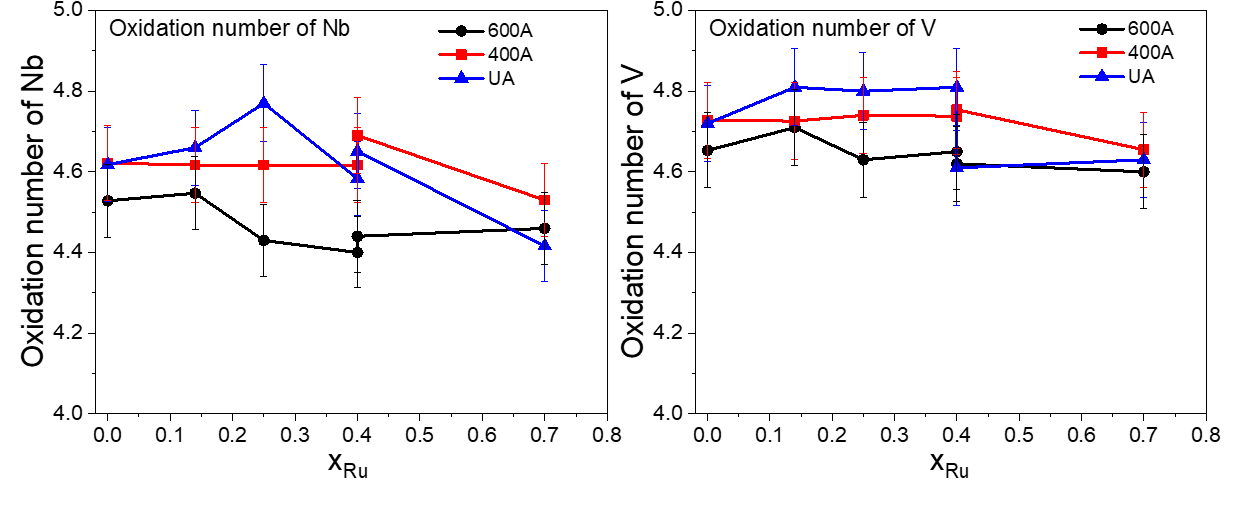


**Figure S4**. (a) Survey scanned XPS of UA, 400A, and 600A samples. (b) Fine-scan XPS peaks of Ru 3*d*_5/2_, Mo 3*d* (3*d*_5/2_ and 3*d*_3/2_ separated by 3.13 eV), V 2*p* (2*p*_3/2_ and 2*p*_1/2_ separated by 7.64 eV), Nb 3*d* (3*d*_5/2_ and 3*d*_3/2_ separated by 2.72 eV), Se 3*d* (3*d*_5/2_ and 3*d*_3/2_ separated by 0.86 eV), and VBS. Positions of neutral Ru^0^ (3*d*_5/2_ at 280.1 eV), Mo^0^ (3*d*_5/2_ at 228.0 eV), V^0^ (2*p*_3/2_ at 512.2 eV), Nb^0^ (3*d*_5/2_ at 202.4 eV), and Se^0^ (3*d*_5/2_ at 55.6 eV) are marked by dotted vertical lines. The experimental data (open circles) are fitted by a Voigt function after a Shirley-type baseline correction. The sum of the resolved bands is represented by a black line. In VBS, the Fermi level (E_F_) is marked by a dotted vertical line. The *d*-band center (ε_d_) value is also labeled with a dotted line arrow to emphasize the redshift with increasing *x*_Ru_. The ε_d_ value (eV) *vs.* *x*_Ru_ is plotted for UA, 400A, and 600A samples. (c) Fraction of resolved bands for Nb 3*d*_5/2_ and V 2*p*_3/2_ peaks. Oxidation number of Nb and V atoms *vs.* *x*_Ru_. The error bar represents ±SD obtained from an average of measurements of at least 3 samples.

(a) Survey scan shows that the intensity of Ru 3*d*_5/2_ peak increases with *x*_Ru_, while that of Mo, V, and Nb peaks decreases.

(b) The first spectrum corresponds to the Ru 3*d*_5/2_ peak; since the Ru 3*d*_3/2_ peak overlaps with the C 1*s* peak, only the Ru 3*d*_5/2_ peak was analyzed. This peak arises from Ru-Se bonding structures. In all alloy samples, the peak appears at 280.5 eV, which is blue-shifted relative to that of neutral Ru (Ru^0^, 280.1 eV). The peak for sample **9-600A** appears at 280.2 eV, indicating a higher degree of metallicity for the C phase RuSe_2_ compared to the alloy phase. The peak was deconvoluted into the Ru1 (56%) and Ru2 components at 280.6 eV and 280.1 eV, respectively, which is assigned to Ru-Se bonding in the 2H/1T and C phase. All 400A and UA samples show the Ru 3*d*_5/2_ peak at ~280.6 eV, which is the same as that of 600A samples of **2** ~ **8**. Therefore, the electronic state of Ru in the alloys is close to the 2H/1T phase RuSe_2_. The annealing at higher temperature (600 ℃) blue shifted the peak of sample **9**, due to the phase conversion into the more metallic C phase.

The Mo 3*d*_5/2_ and 3*d*_3/2_ peaks (separated by 3.13 eV.) were resolved into the Mo1 (main) and Mo2 bands that correspond to Mo-Se bonds and defects, respectively. The Mo1 and Mo2 components of 3*d*_5/2_ peak appeared at 228.0-228.2 eV and 229.5-219.7 eV, respectively, for all 600A, 400A and UA samples. The Mo1 peak exhibits a negligible shift relative to neutral Mo (Mo^0^, 228.0 eV), suggesting that all alloys exhibit metallic character. The metallic electronic state of Mo retains upon the annealing.

The Nb 3*d*_5/2_ and 3*d*_3/2_ peaks are separated by 2.72 eV. The 3*d*_5/2_ peak includes two components at 203.7-204.0 eV (Nb1) and 207.3-207.5 eV (Nb2), which are blue shifted from neutral Nb (202.4 eV) by 1.3-1.6 eV and 4.9-5.1 eV, respectively. The Nb1 band is assigned to Nb–Se bonding with Nb^4+^ states, while the Nb2 band is attributed to surface defects (Nb^5+^ state). Incorporation of Ru leads to a decrease in the Nb^5+^ fraction, indicating that quaternary alloying suppresses Nb oxidation. As shown in (c), the calculated oxidation state of Nb (based on the fraction of Nb1 and Nb2 bands of Nb 3*d*_5/2_) decreases as *x*_Ru_ increases to 0.7.

The V 2*p* peaks were deconvoluted into V1 (V-Se bond, V^4+^) and V2 (V-O bond, V^5+^) components; the V1 peak appears at 513 eV and the V2 component at 517-518 eV for the V 2*p*_3/2_ peak. The fraction of the V2 band decreases with increasing *x*_Ru_. As shown in (c), the Ru incorporated, and then the fraction of the V2 band (V 2*p*_3/2_) decreased, indicating that that the alloying protects the oxidation of V atoms.

The Se 3*d* peak (with 3*d*_5/2_ and 3*d*_3/2_ components separated by 0.8 eV), located at 54-55 eV, is red-shifted relative to neutral Se (Se^0^, 55.6 eV). This peak was deconvoluted into Se1 (and Se2 components, corresponding to Se-metal (M) bonds and surface defects, respectively. As *x*_Ru_ increases, the fraction of the Se2 component decreases. For 600A samples, the Se1 band blue-shifts from 53.7 eV to 54.7 eV with increasing *x*_Ru_, which is likely associated with the phase transition to the C phase RuSe_2_ phase. The 400A and UA alloy samples show the Se1 band of 3*d*_5/2_ at 54 eV. In the case of sample **9**, the UA and 400 A samples show the peak at 54.0 and 54.3 eV. The red shift of 600 A sample is originated from the 2H → C phase transition.

The final XPS is the valence band spectrum (VBS). The onset of the VBS indicates the valence band maximum (VBM) below the Fermi level (E_F_). All samples exhibit VBM = 0 eV, consistent with a metallic character. Integration of the VBS from 0 to 10 eV yields the *d*-band center (ε_d_). Incorporation of Ru shifts ε_d_ closer to E_F_, indicating an increase in metallicity. The phase conversion into more metallic 1T probably contributes to increasing the metallicity at higher *x*_Ru_. The 600A samples exhibited the significant redshift of ε_d_, probably due to the phase conversion from the 2H/1T phase into the more metallic C phase RuSe_2_. The ε_d_ value *vs.* *x*_Ru_ is plotted in (c).

In summary, the quaternary alloy nanosheets possess greater metallic character than the (MoVNb)Se_2_, with this effect becoming more pronounced at higher *x*_Ru_. The 2H/1T phase RuSe_2_ is less metallic than the C phase, resulting in quaternary alloys being less metallic in UA and 400A samples compared to the 600A samples. Incorporation of Ru into (MoVNb)Se_2_ enhances the metallic character, accompanied by lower oxidation states of V and Nb.

(a)
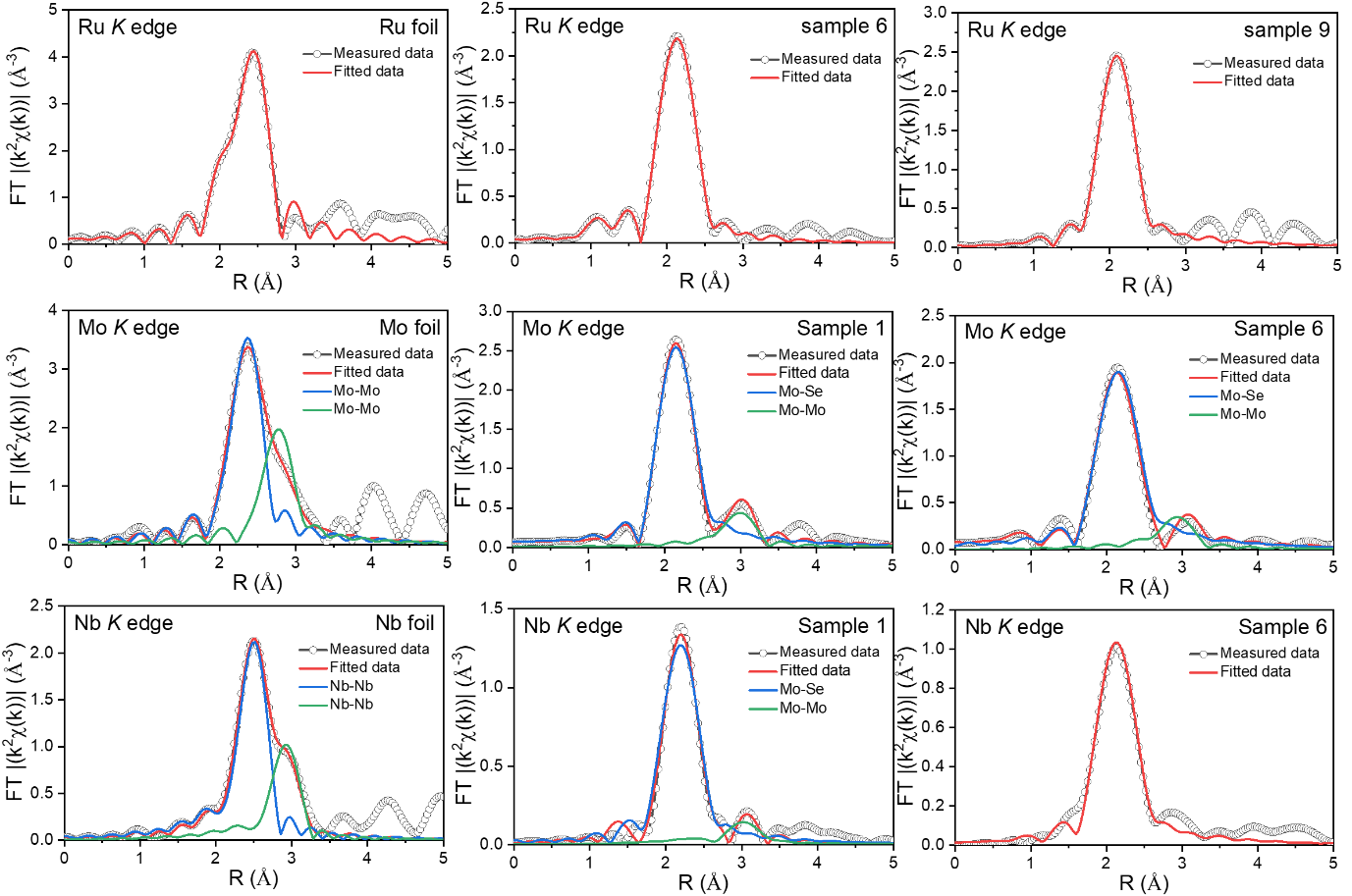


(b)


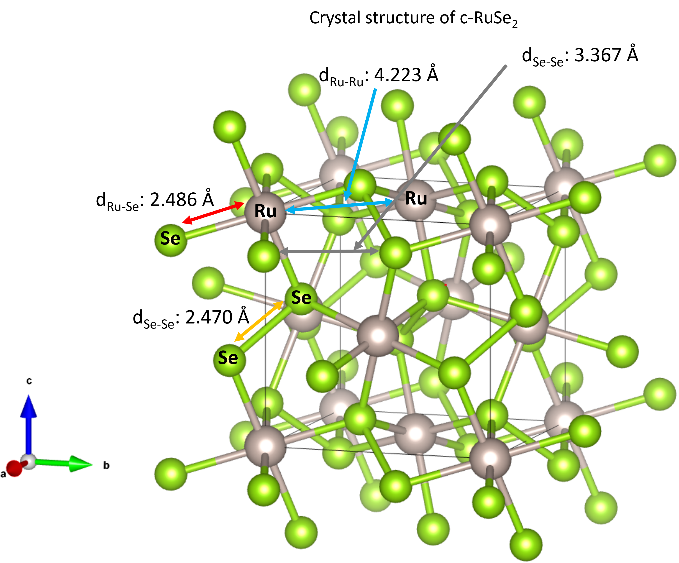


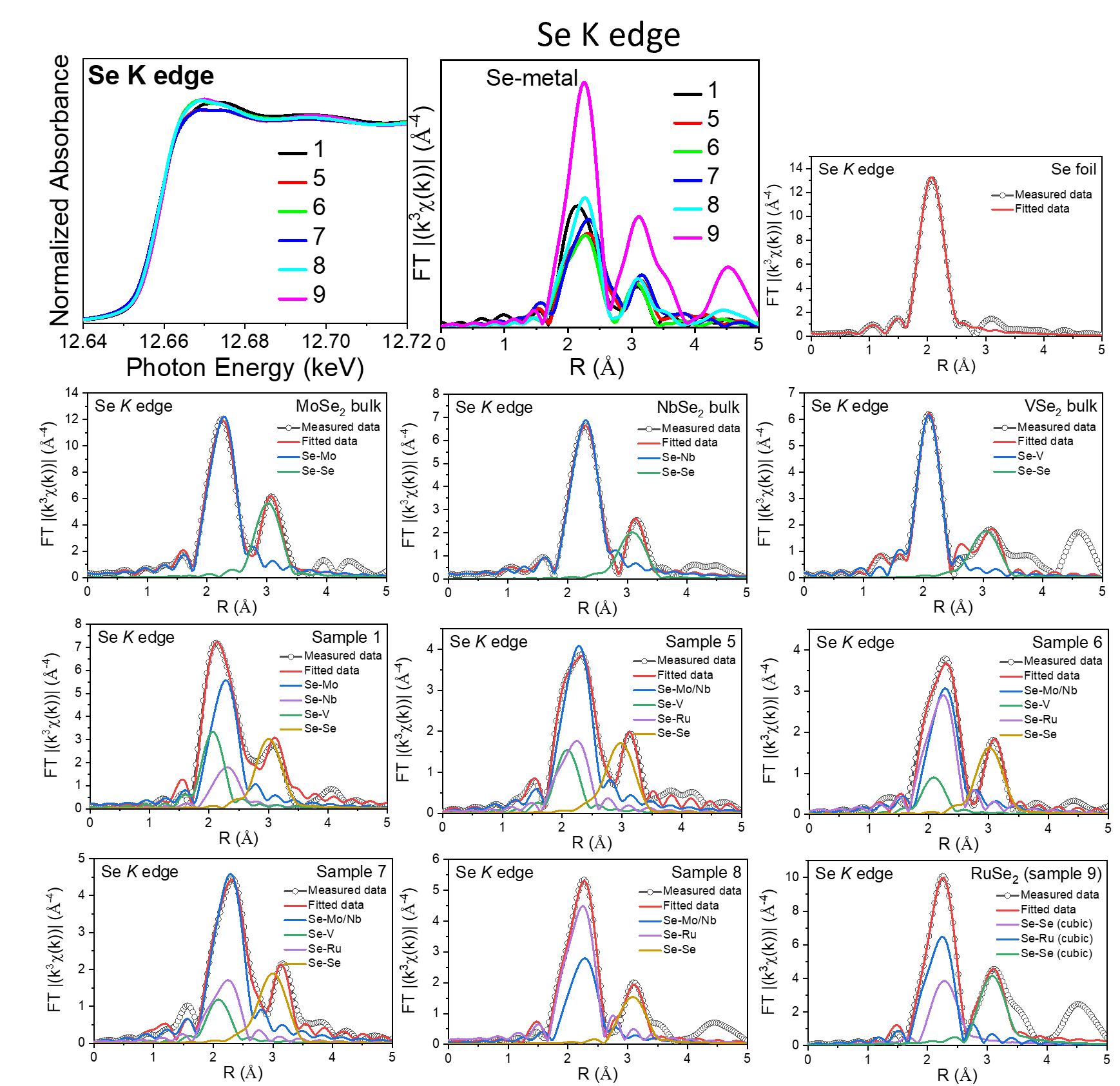


(c)


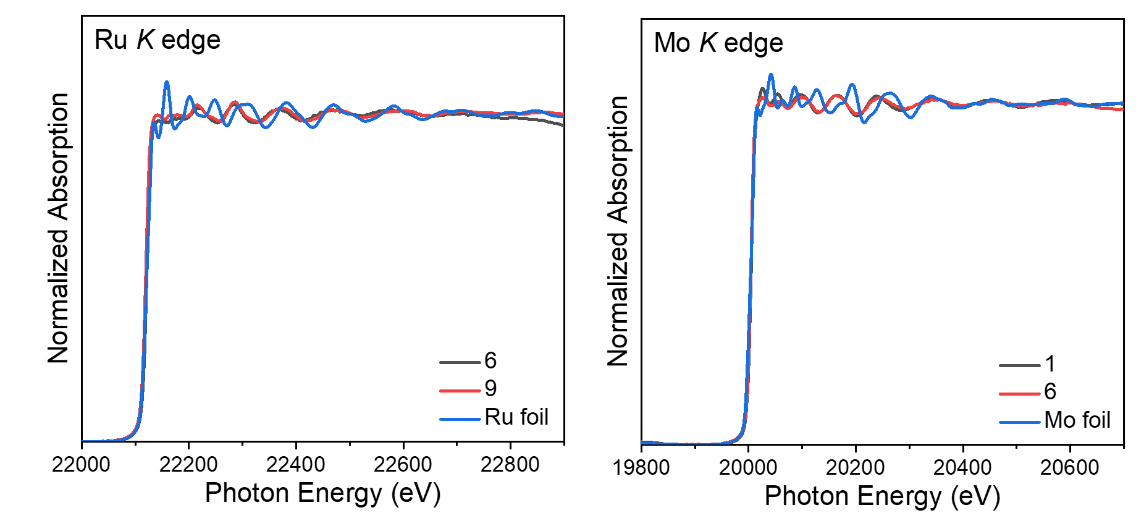


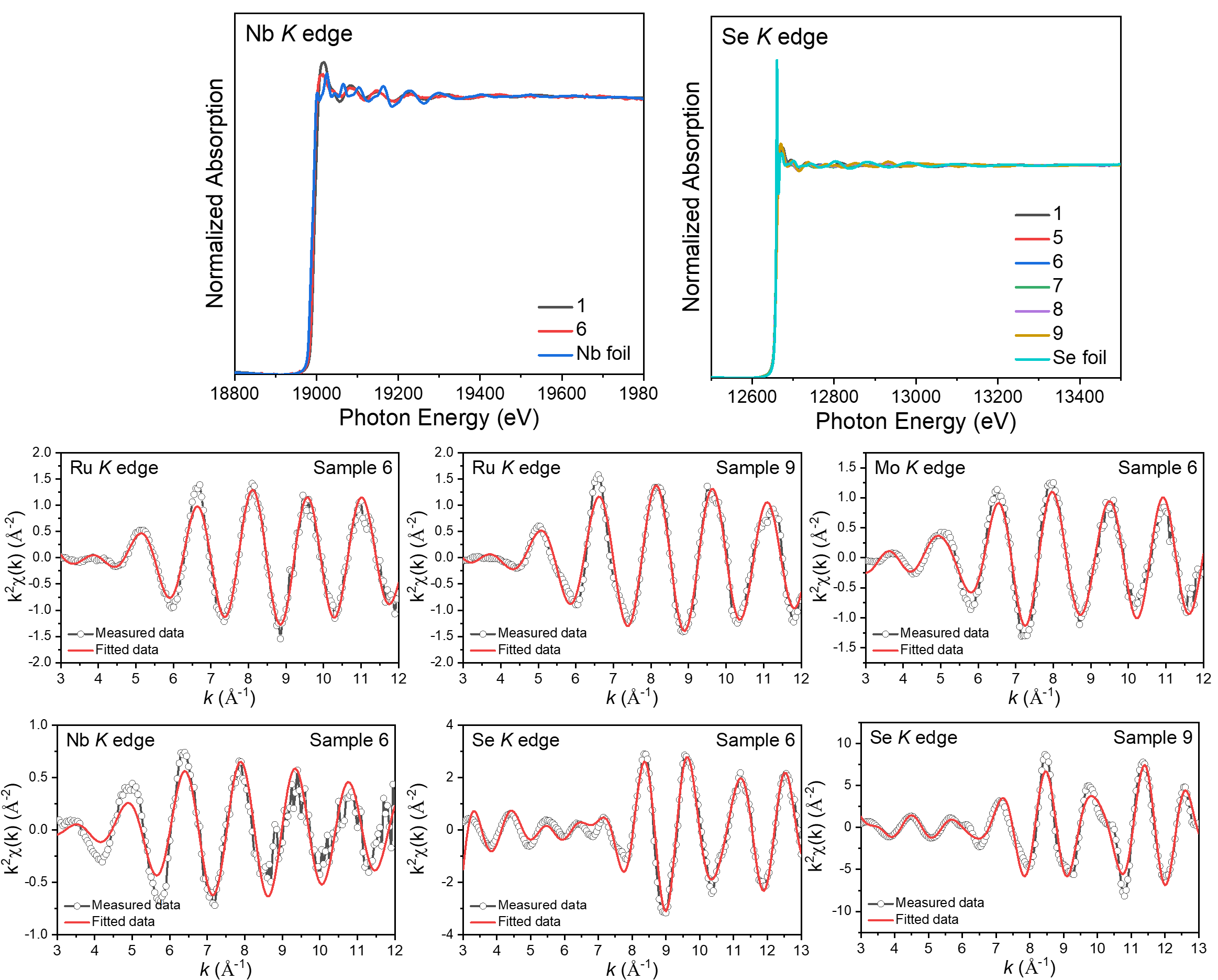


(d)


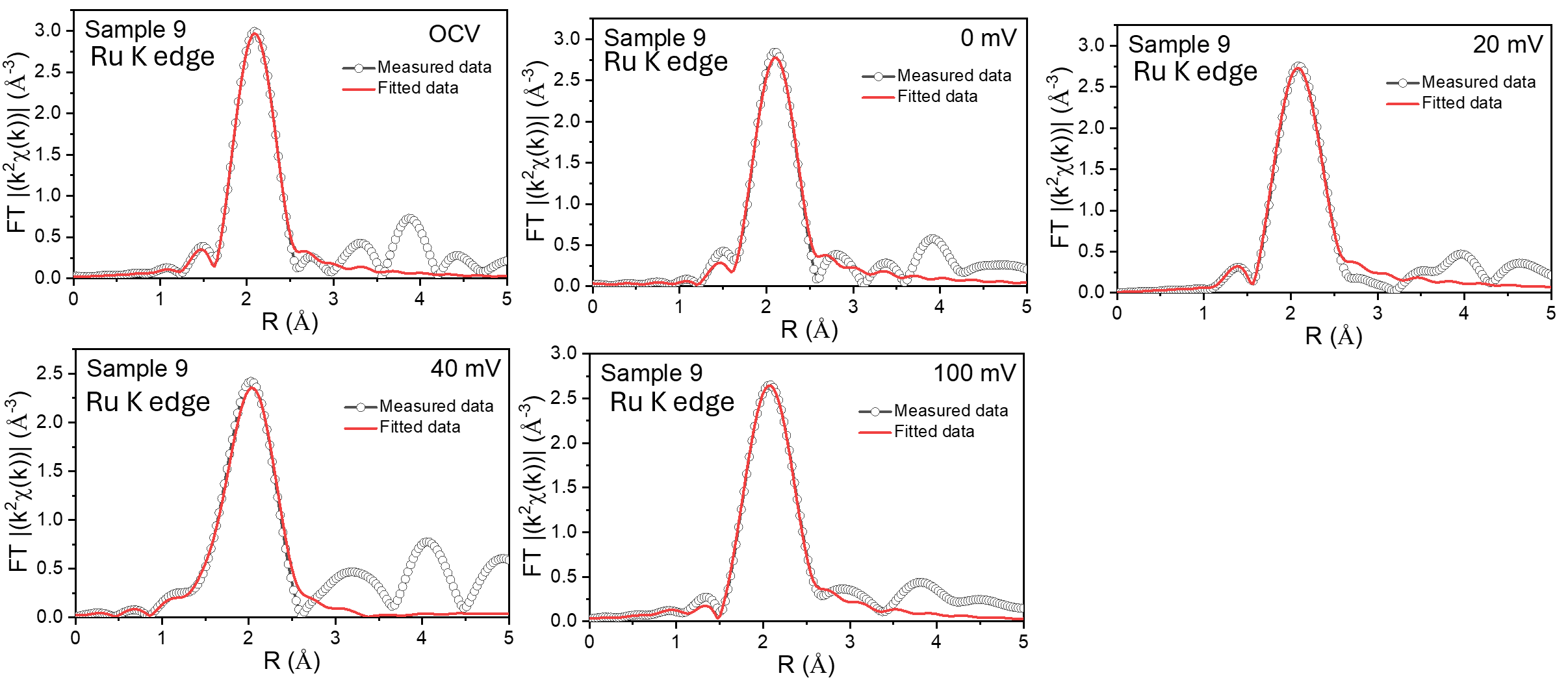


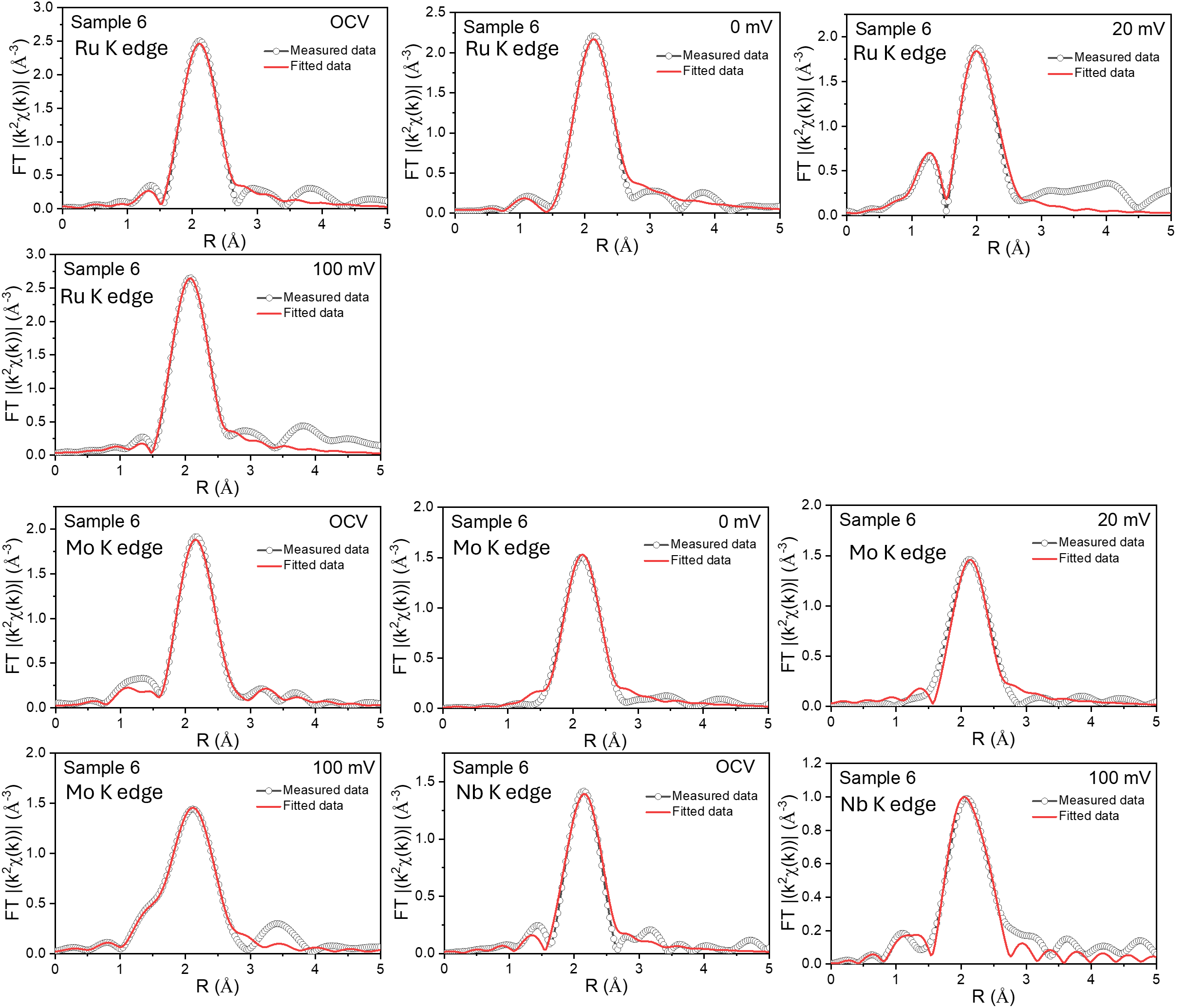


(e)


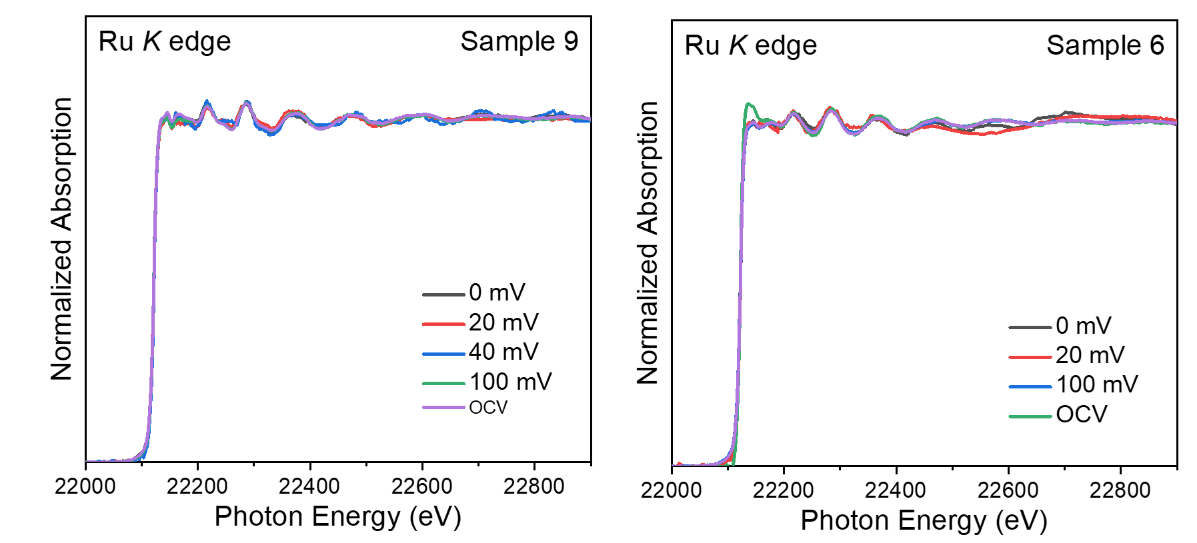


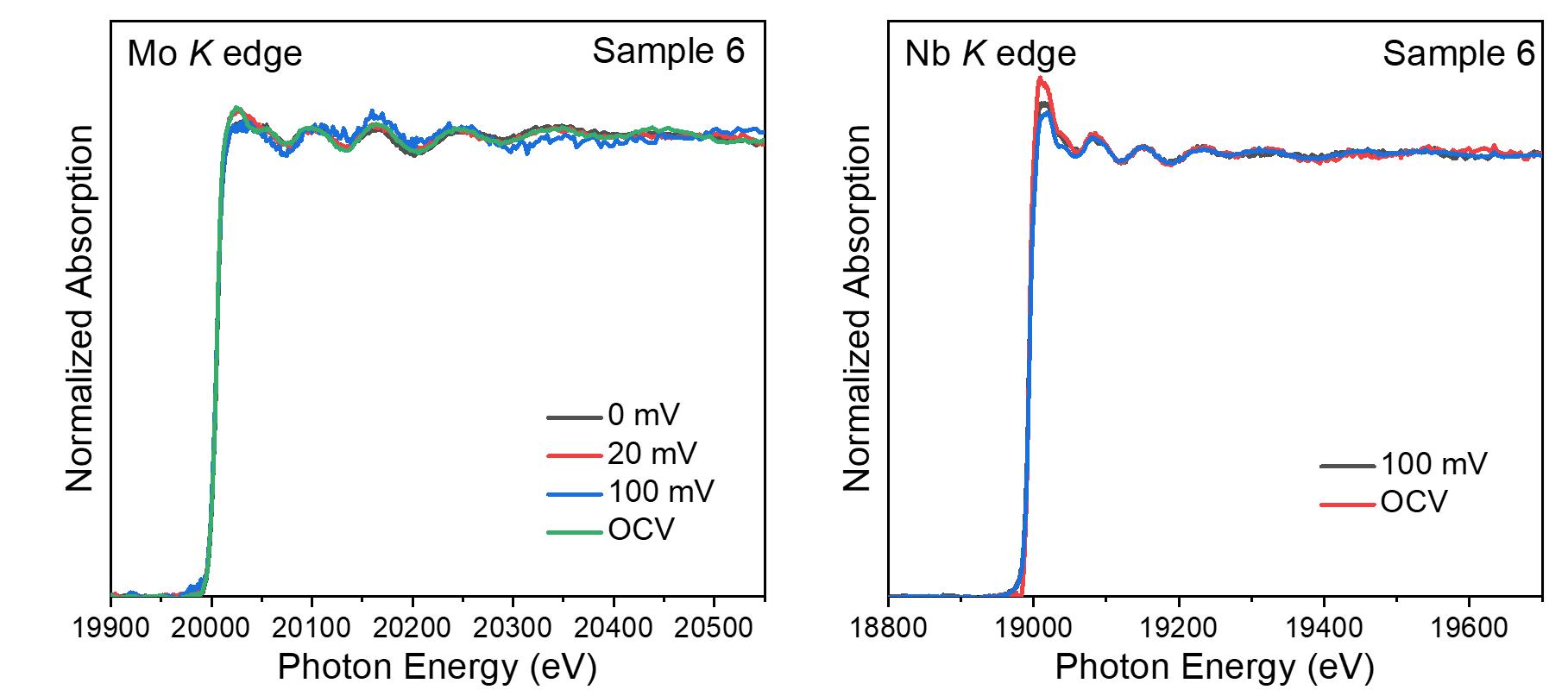

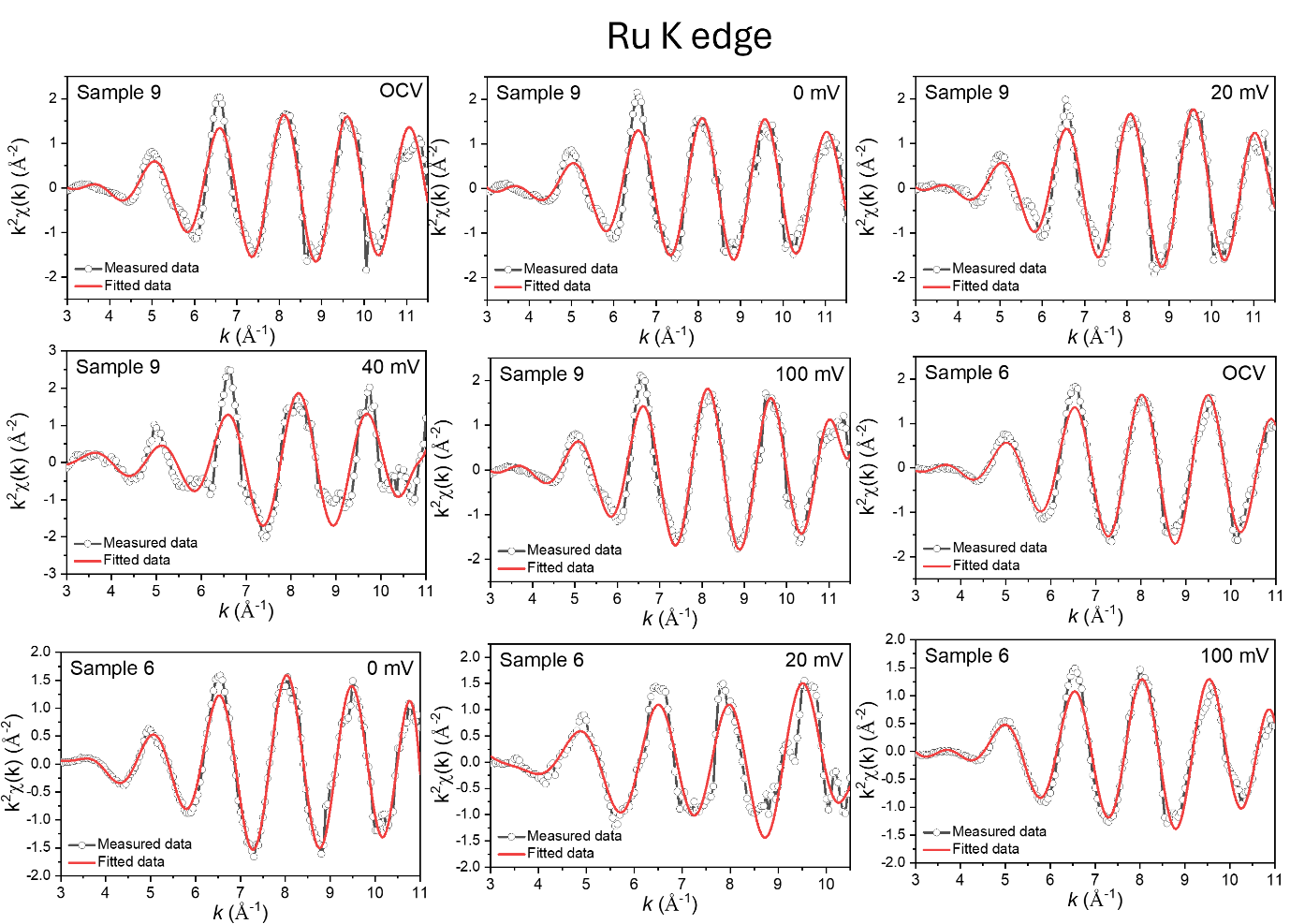
**Figure S5**. (a) Curve fitting of k^2^-weighted FT EXAFS above the Ru K edge (22.1 keV), Mo K edge (20.01 keV), and Nb K edge (18.98 keV) for metal (Ru, no, and Nb) foil, samples **1**, **6**, and **9** (**Figure 3a)**. (b) XANES, FT EXAFS, and curve fitting of k^3^-weighted FT EXAFS above the Se K edge (at 12.64 keV) for Se foil, samples **1**, **5**, **6**, **7**, **8**, and **9**. Interatomic distance and coordination number were obtained by the curve fitting. The fitting parameters are summarized in **Table S2**. (b) The crystal structure of C phase RuSe_2_ shows the two distances of Se-Se with *d*_Se-Se_ = 2.470 and 3.367 Å and *d*_Ru-Se_ = 2.486 Å, consistently the fitting parameters of sample **9**. To support the peak fitting, FT EXAFS data for bulk MoSe_2_, NbSe_2_, and VSe_2_ are shown. The Ru-Se peak increases with increasing *x*_Ru_, showing the successful composition tuning from the ternary alloy (sample 1) to quaternary alloys (samples **5**-**8**), and C-phase RuSe_2_. (c) The raw absorption spectrum (E space) and the k-space oscillation functions χ(k) for Ru K-edge, Mo K edge, Nb-edge, and Se-edge revealed the validity of fitting curves. (d) Curve fitting of *in-situ*/e*x-situ* (non-phase-corrected) *k*^2^-weighted FT EXAFS above Ru K edge, Mo K edge, and Nb K edge for sample **6** and **9** after applying overpotential 𝜂 = 0–100 mV under HER conditions (**Figure 5c**). Interatomic distance and coordination number were obtained by the curve fitting, as summarized in **Table S2**. (e) The raw absorption spectrum (E space) and the k-space oscillation functions χ(k) for *in-situ*/e*x-situ* Ru K edge of samples **6** and **9** revealed the validity of fitting curves,


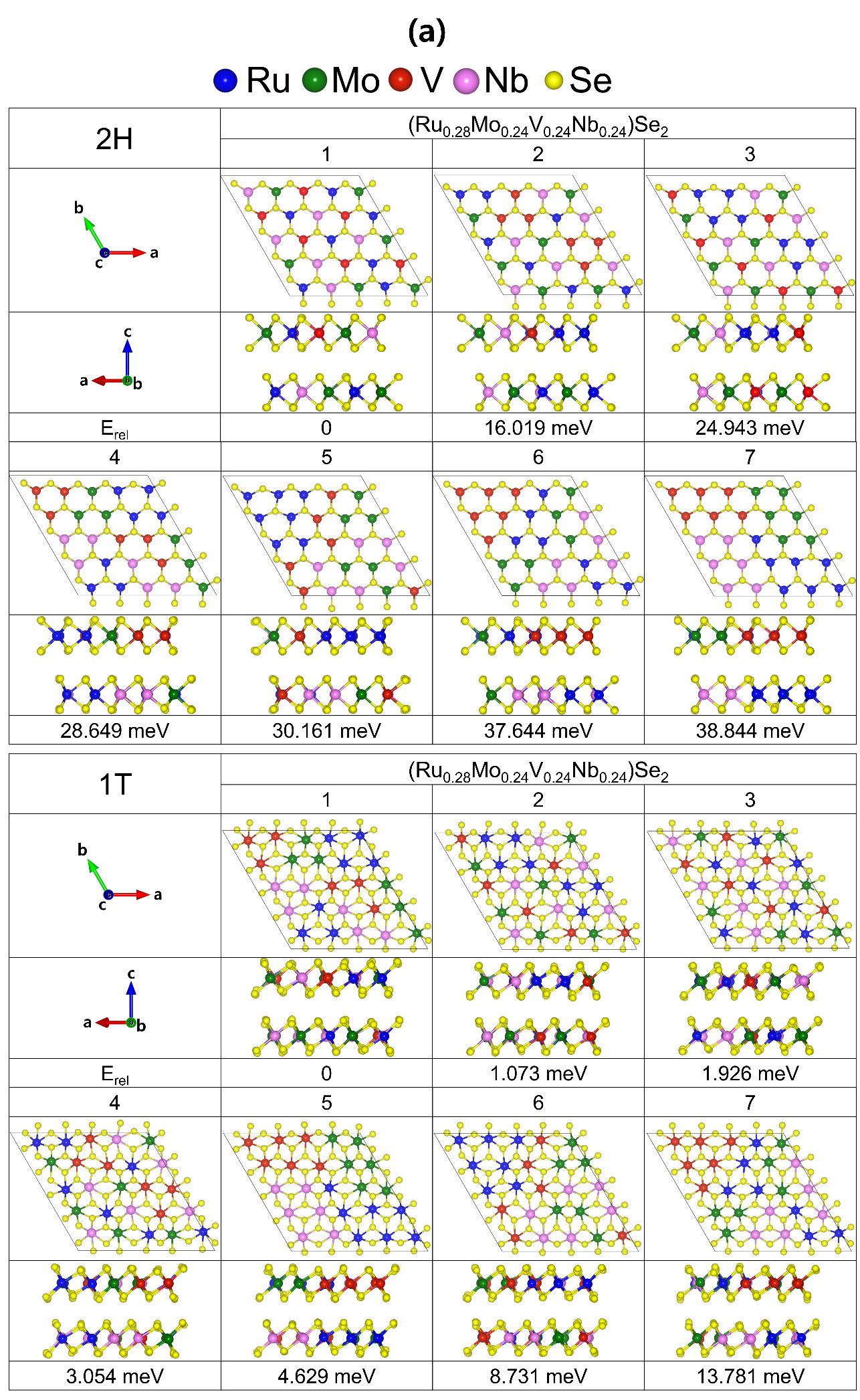


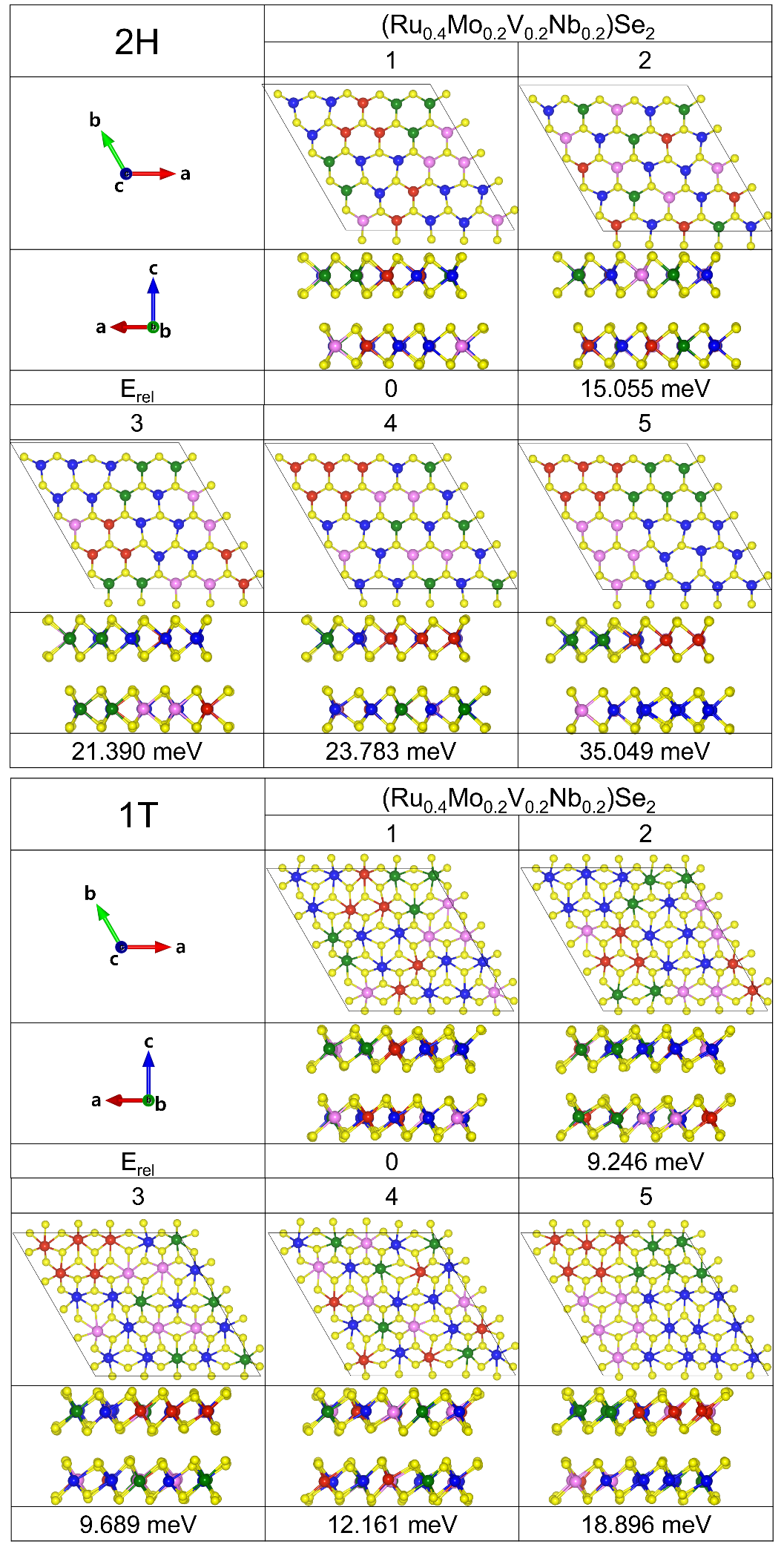


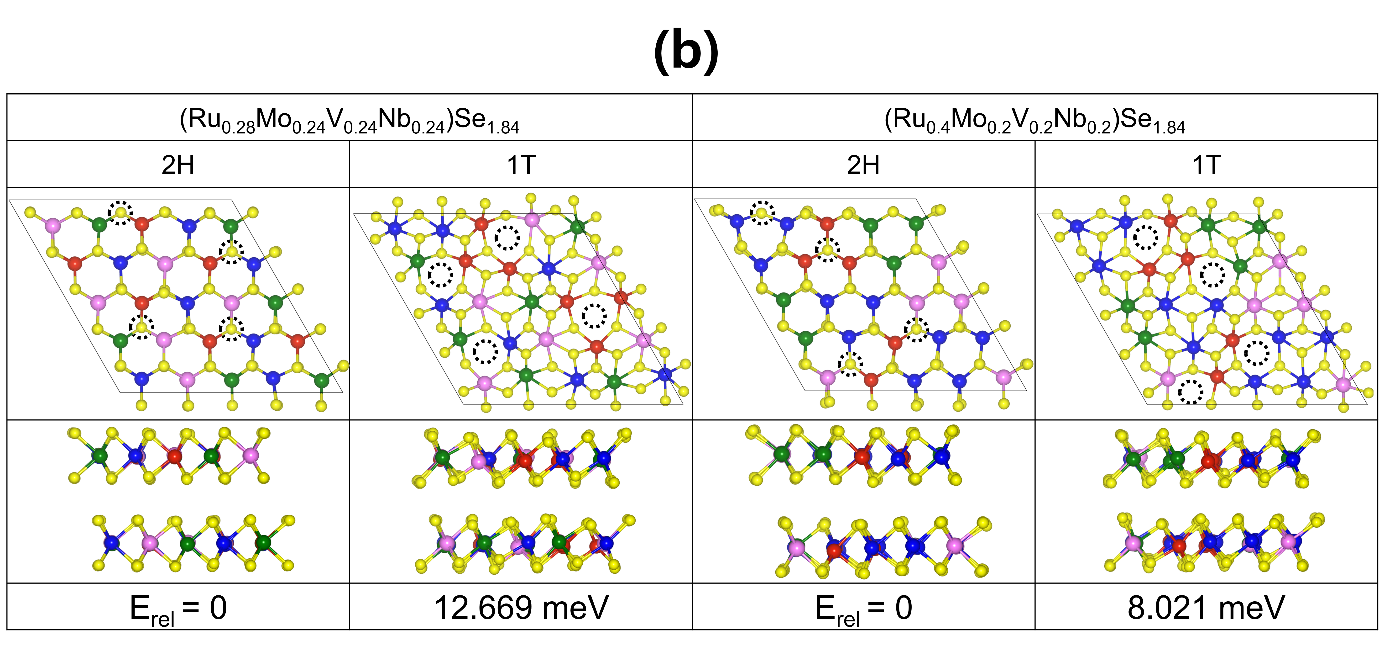
 **Figure S6**. Configurations of 2H and 1T phase (Ru_0.28_Mo_0.24_V_0.24_Nb_0.24_)Se_2_ (*x*_Ru_ = 0.28) and (Ru_0.4_Mo_0.2_V_0.2_Nb_0.2_)Se_2_ (*x*_Ru_ = 0.4). The (a) pristine and (b) Se vacancy models were built from (5 × 5 × 2) supercell. Blue, green, red, pink, and yellow balls represent Ru, Mo, V, Nb, and Se atoms, respectively. The relative energy (E_rel_) per atom is with respect to the 2H phase. The Se vacancies are marked by dotted circles.


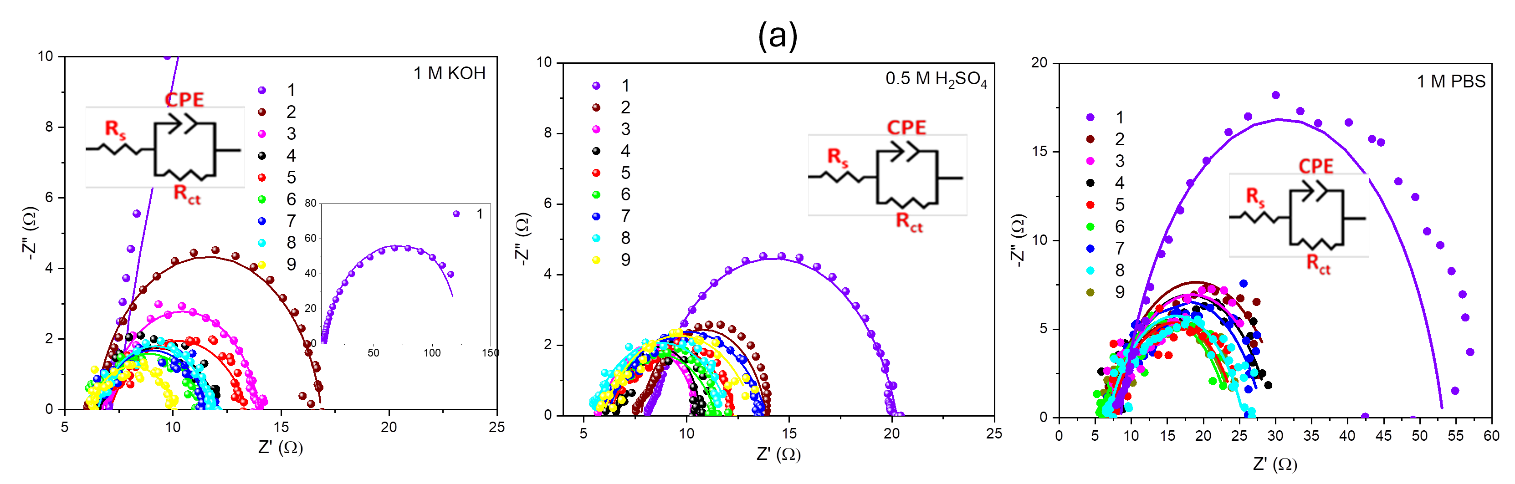

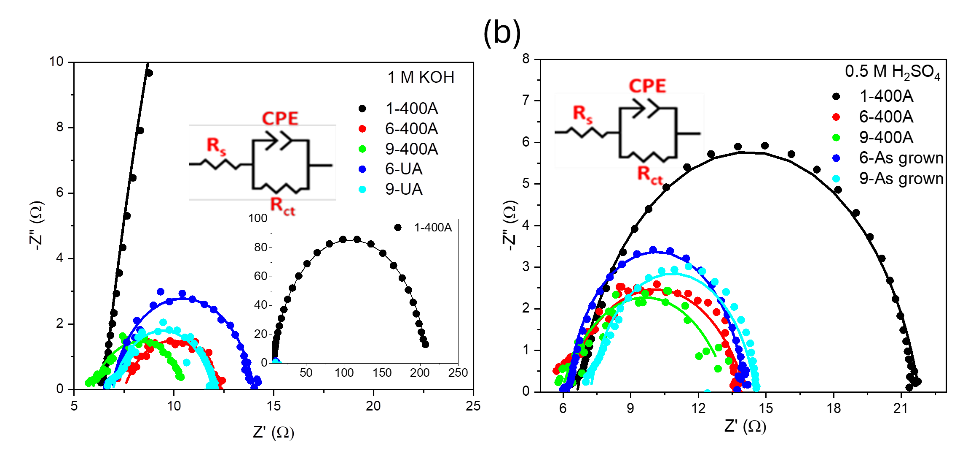


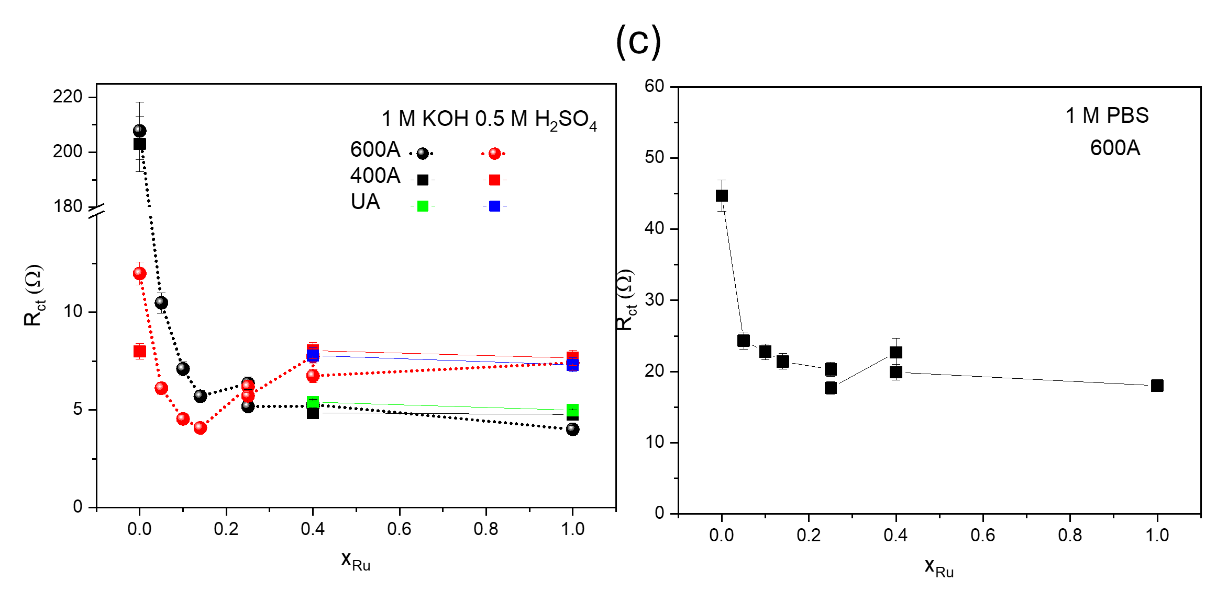


**Figure S7**. Nyquist plots of (a) 600A and (b) 400A/UA samples **1**-**9** using the frequency in the range from 100 kHz to 0.1 Hz at a potential of -0.20 V, -0.10 V, and -0.20 V in 1 M KOH, 0.5 M H_2_SO_4_, and 1 M PBS electrolyte respectively. The modified Randles circuit for fitting is shown in the inset. (c) *R*_ct_ *vs.* *x*_Ru_ for (RuMoVNb)Se_2_. The error bar represents ±SD obtained from an average of measurements of at least 3 samples.

Electrochemical impedance spectroscopy (EIS) measurements of the samples were performed using an amplitude of 10 mV. In the high-frequency limit and under non-Faradaic conditions, the electrochemical system is approximated by the modified Randles circuit shown in the inset, where *R*_s_ denotes the solution resistance, CPE is a constant-phase element related to the double-layer capacitance, and *R*_ct_ is the charge-transfer resistance from any residual Faradaic processes. A semicircle in the low-frequency region of the Nyquist plots represents the charge transfer process, with the diameter of the semicircle reflecting the charge-transfer resistance. The real (*Z*′) and negative imaginary (-*Z*′′) components of the impedance are plotted on the *x* and *y* axes, respectively. The simulation of the EIS spectra using an equivalent circuit model allowed us to determine the charge transfer resistance, *R*_ct_, which is a key parameter for characterizing the catalyst-electrolyte charge transfer process. The fitting parameters are summarized as follows. A smaller *R_ct_* with increasing *x*_V_ implies more facile electron transfer kinetics that enhances catalytic activity.

[**Fitting Impedance parameters for the equivalent circuit**]

(a) 600A

[1 M KOH]

| Sample No*.* | R_S_ (Ω) | CPE (mF) | R_ct_ (Ω) |
| --- | --- | --- | --- |
| 1 | 7.01 ± 0.21 | 5.96 ± 0.18 | 207.80 ± 4.30 |
| 2 | 6.38 ± 0.19 | 9.66 ± 0.29 | 10.48 ± 0.52 |
| 3 | 6.89 ± 0.21 | 9.22 ± 0.28 | 7.10 ± 0.31 |
| 4 | 6.40 ± 0.91 | 0.14 ± 0.01 | 0.5.70 ± 0.23 |
| 5 | 6.90 ± 0.21 | 9.01 ± 0.27 | 6.36 ± 0.23 |
| 6 | 6.25 ± 0.19 | 7.95 ± 0.24 | 5.18 ± 0.12 |
| 7 | 6.44 ± 0.19 | 8.93 ± 0.27 | 5.19 ± 0.11 |
| 8 | 6.52 ± 0.20 | 0.11 ± 0.01 | 5.28 ± 0.15 |
| 9 | 6.12 ± 0.18 | 0.13 ± 0.01 | 4.01 ± 0.16 |

[0.5 M H_2_SO_4_]

| *x* | R_S_ (Ω) | CPE (mF) | R_ct_ (Ω) |
| --- | --- | --- | --- |
| 1 | 7.82 ± 0.24 | 6.68 ± 0.29 | 11.98 ± 0.43 |
| 2 | 7.67 ± 0.23 | 4.78 ± 0.14 | 6.11 ± 0.33 |
| 3 | 5.92 ± 0.18 | 4.48 ± 0.13 | 4.54 ± 0.21 |
| 4 | 6.56 ± 0.20 | 4.07 ± 0.12 | 4.08 ± 0.10 |
| 5 | 6.13 ± 0.18 | 6.44 ± 0.19 | 7.75 ± 0.25 |
| 6 | 5.72 ± 0.17 | 7.52 ± 0.23 | 5.72 ± 0.13 |
| 7 | 6.24 ± 0.19 | 5.94 ± 0.18 | 6.19 ± 0.15 |
| 8 | 5.38 ± 0.16 | 7.35 ± 0.22 | 6.74 ± 0.15 |
| 9 | 5.92 ± 0.18 | 7.82 ± 0.23 | 7.41 ± 0.23 |

[1 M PBS]

| *x* | R_S_ (Ω) | CPE (mF) | R_ct_ (Ω) |
| --- | --- | --- | --- |
| 1 | 7.46 ± 0.22 | 2.80 ± 0.08 | 44.70 ± 2.21 |
| 2 | 6.57 ± 0.20 | 0.18 ± 0.01 | 24.34 ± 1.25 |
| 3 | 6.90 ± 0.21 | 0.16 ± 0.01 | 22.80 ± 1.13 |
| 4 | 7.20 ± 0.22 | 0.16 ± 0.01 | 21.41 ± 1.14 |
| 5 | 7.30 ± 0.22 | 0.11 ± 0.01 | 20.30 ± 1.08 |
| 6 | 6.00 ± 0.18 | 0.15 ± 0.01 | 17.70 ± 0.95 |
| 7 | 7.12 ± 0.21 | 0.19 ± 0.01 | 22.70 ± 1.04 |
| 8 | 6.50 ± 0.20 | 0.14 ± 0.01 | 19.91 ± 1.17 |
| 9 | 6.30 ± 0.19 | 0.14 ± 0.01 | 18.01 ± 0.83 |

(b) 400A and UA

[1 M KOH]

| Sample No*.* | R_S_(Ω) | CPE (mF) | R_ct_ (Ω) |
| --- | --- | --- | --- |
| 1-400A | 6.48 ± 0.19 | 2.45 ± 0.07 | 203.0 ± 4.0 |
| 6-400A | 7.49 ± 0.22 | 8.95 ± 0.27 | 4.86 ± 0.15 |
| 9-400A | 5.90 ± 0.18 | 0.16 ± 0.01 | 4.77 ± 0.15 |
| 6-UA | 6.96 ± 0.21 | 8.62 ± 0.26 | 5.41 ± 0.23 |
| 9-UA | 6.94 ± 0.21 | 0.11 ± 0.01 | 4.99 ± 0.16 |

[0.5 M H_2_SO_4_]

| Sample No*.* | R_S_(Ω) | CPE (mF) | R_ct_ (Ω) |
| --- | --- | --- | --- |
| 1-400A | 6.29 ± 0.19 | 10.73 ± 0.32 | 7.99 ± 0.30 |
| 6-400A | 6.60 ± 0.20 | 0.10 ± 0.01 | 8.04 ± 0.30 |
| 9-400A | 6.92 ± 0.21 | 7.41 ± 0.22 | 7.66 ± 0.25 |
| 6-UA | 6.23 ± 0.19 | 0.16 ± 0.01 | 7.79 ± 0.25 |
| 9-UA | 6.99 ± 0.21 | 3.18 ± 0.01 | 7.31 ± 0.20 |


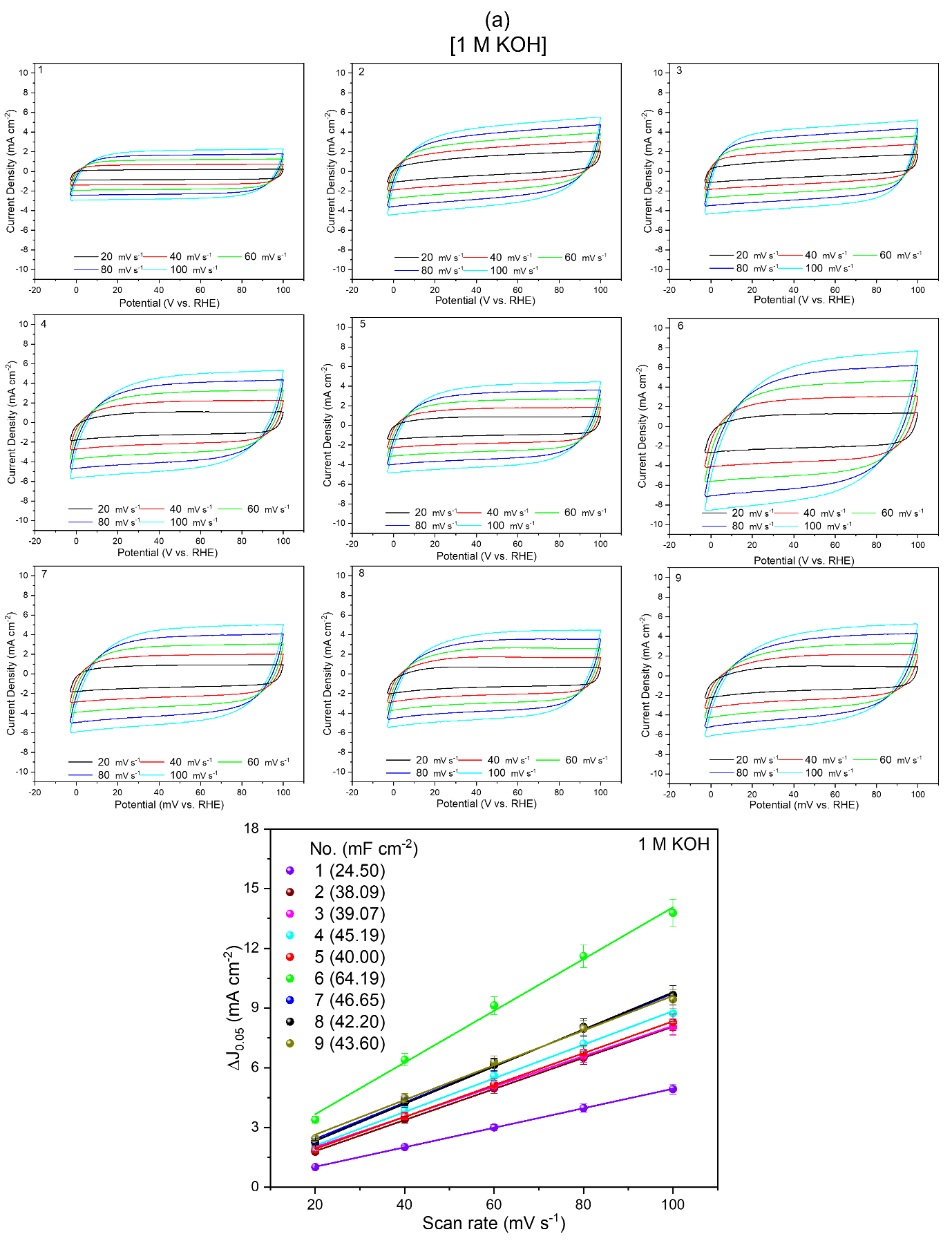

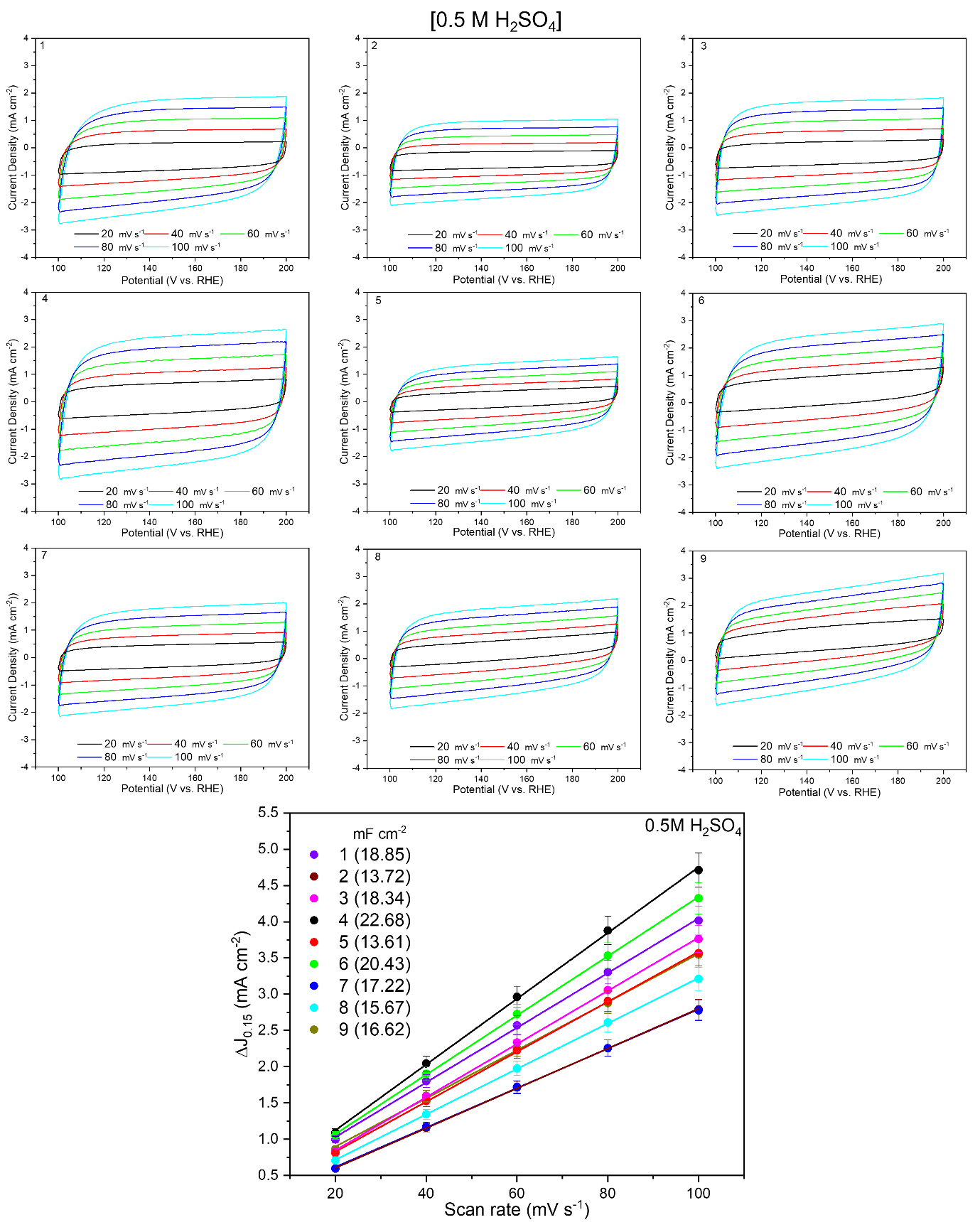


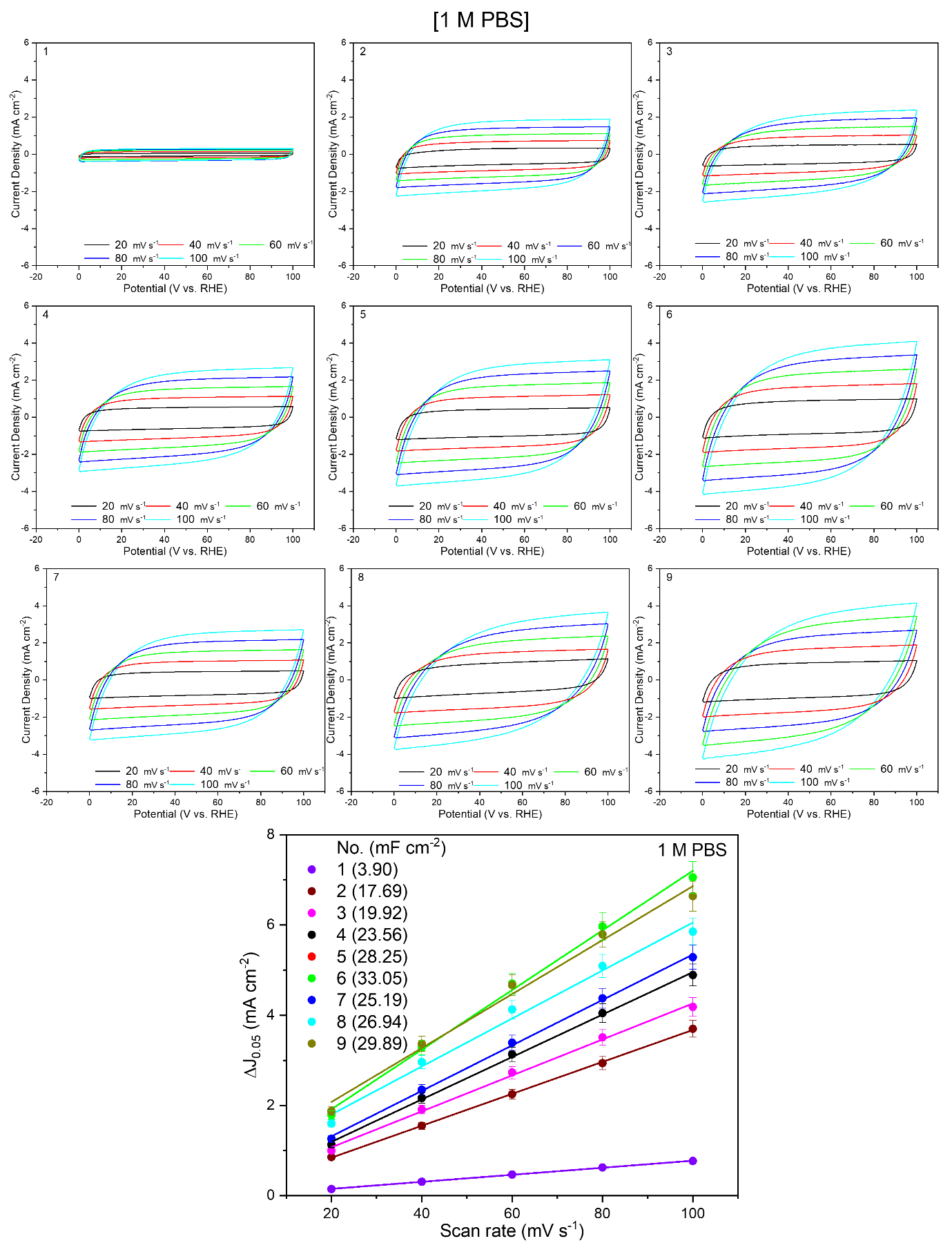


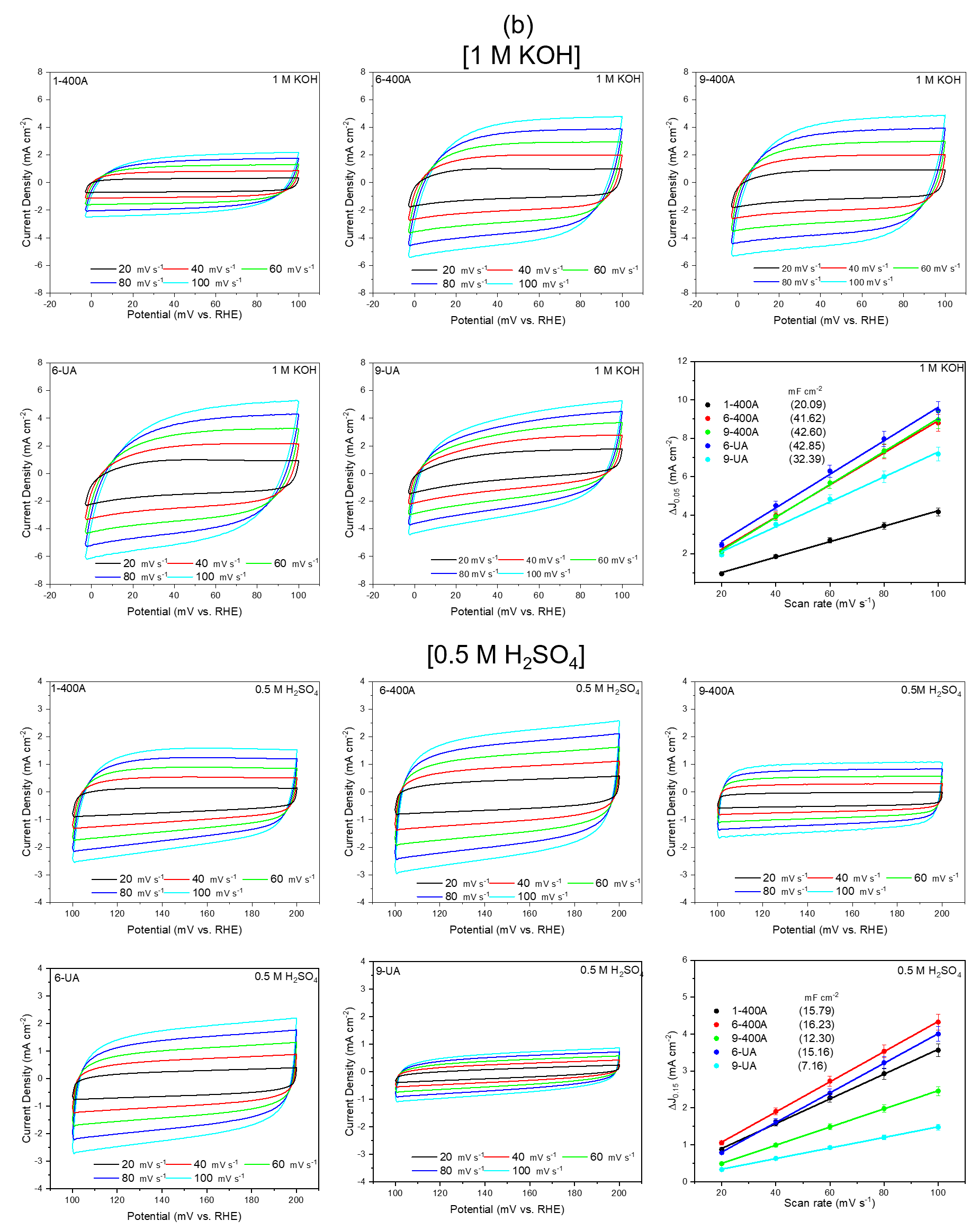
**Figure S8**. Cyclic voltammetry (CV) curves of (a) 600A and (b) 400A/UA samples in 1 M KOH, 0.5 M H_2_SO_4_, and 1 M PBS electrolytes in a non-Faradaic region at 20-100 mV s^-1^ scan rates (with a step of 20 mV s^-1^). The scan range is 0−0.1 V, 0.1−0.2 V, and 0-0.1 V *vs.* RHE, in 1 M KOH, 0.5 M H_2_SO_4_, and 1 M PBS electrolyte respectively. Difference (Δ*J*) between the anodic charging and cathodic discharging currents measured at 0.05 V, 0.15 V, and 0.05 V *vs.* RHE in 1 M KOH, 0.5 M H_2_SO_4_, and 1 M PBS, respectively and plotted as a function of the scan rate. The value in parenthesis represents the C*_dl_*, obtained by the half of the linear slope. The error bar represents ±SD obtained from an average of measurements of at least 3 samples.


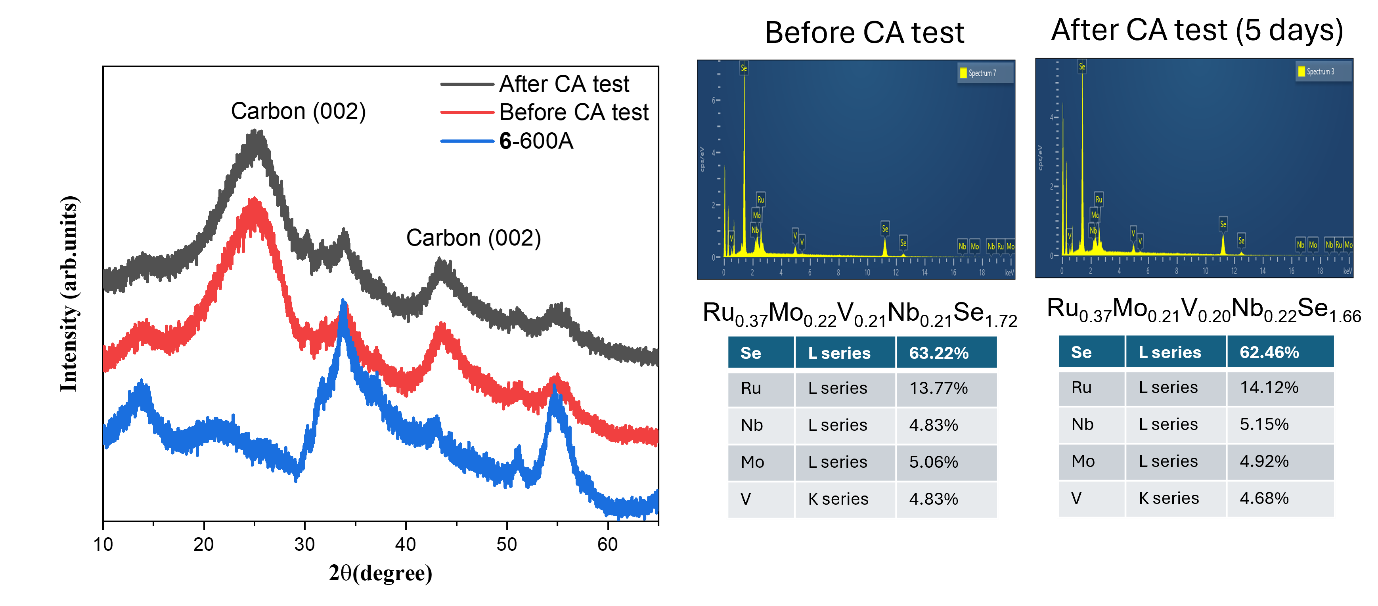

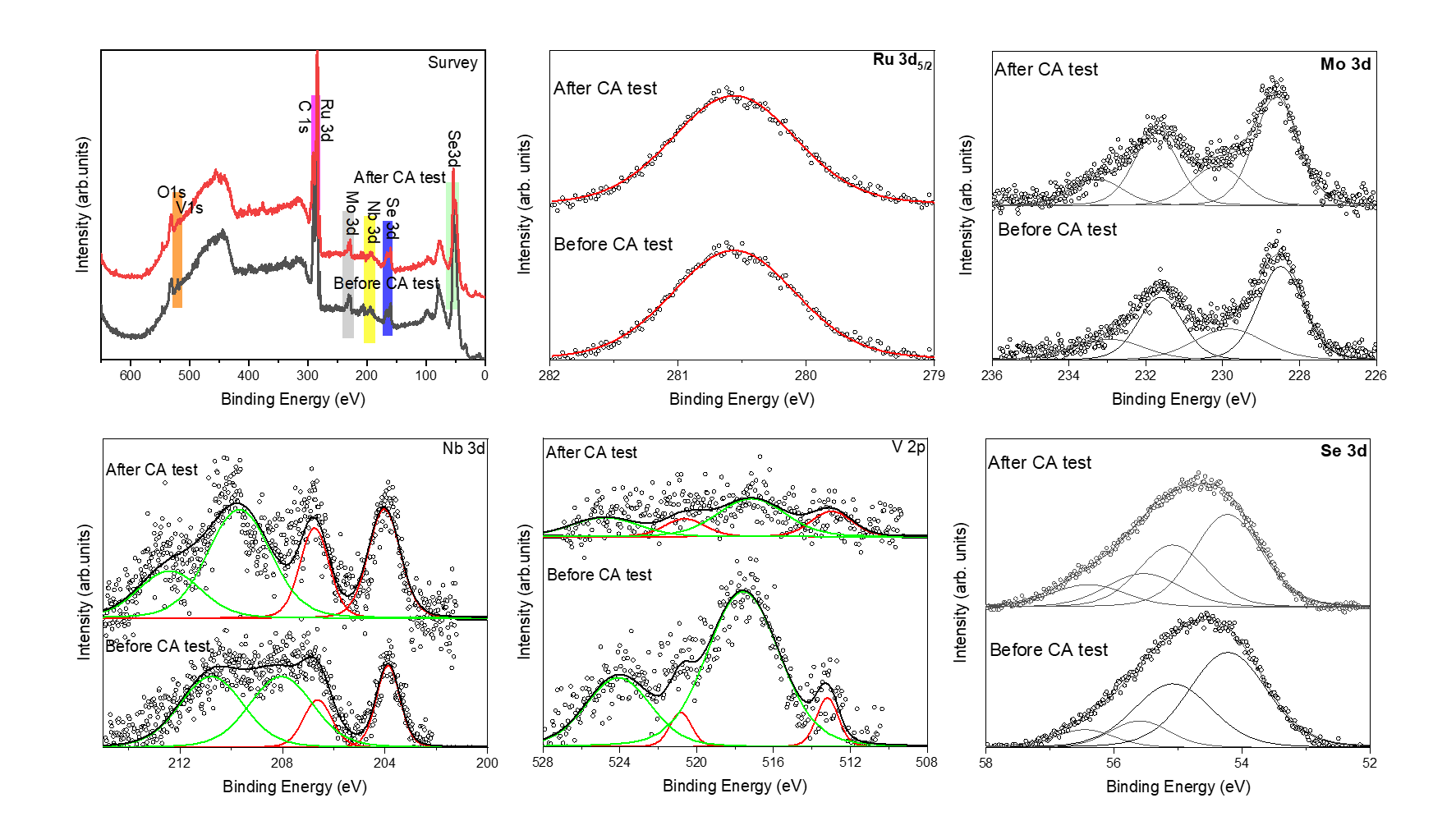


**Figure S9**. Characterization of sample **6** before/after 5-day CA test. XRD patterns showed that the phase maintains after CA test. The EDX spectrum shows that the composition of Ru, Mo, V, Nb, and Se atoms remain the same. No Pt contamination from the dissolved Pt counter electrode was not detected. The XPS also confirmed that no Pt deposition occurred (no 4*f*_5/2_ and 4*f*_7/2_ peaks at 74 and 71 eV, respectively), and the electronic structures of the samples retained during the HER. The experimental data (open circles) are fitted by a Voigt function, after the background correction with a Shirley-type baseline. After the CA test, the adsorption of electrolyte increases the higher-oxidation states for V. Nevertheless, the metallic properties remained.


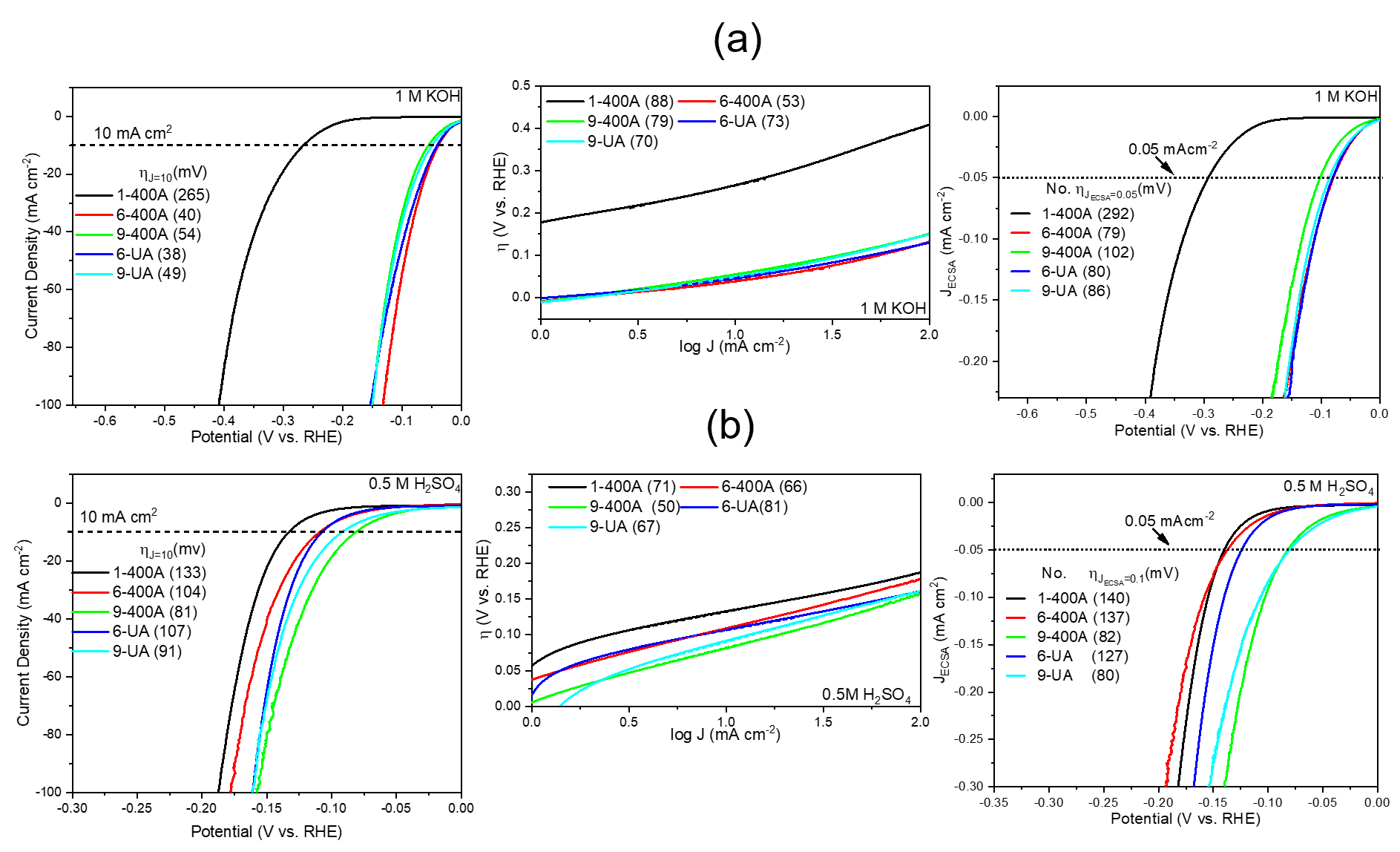


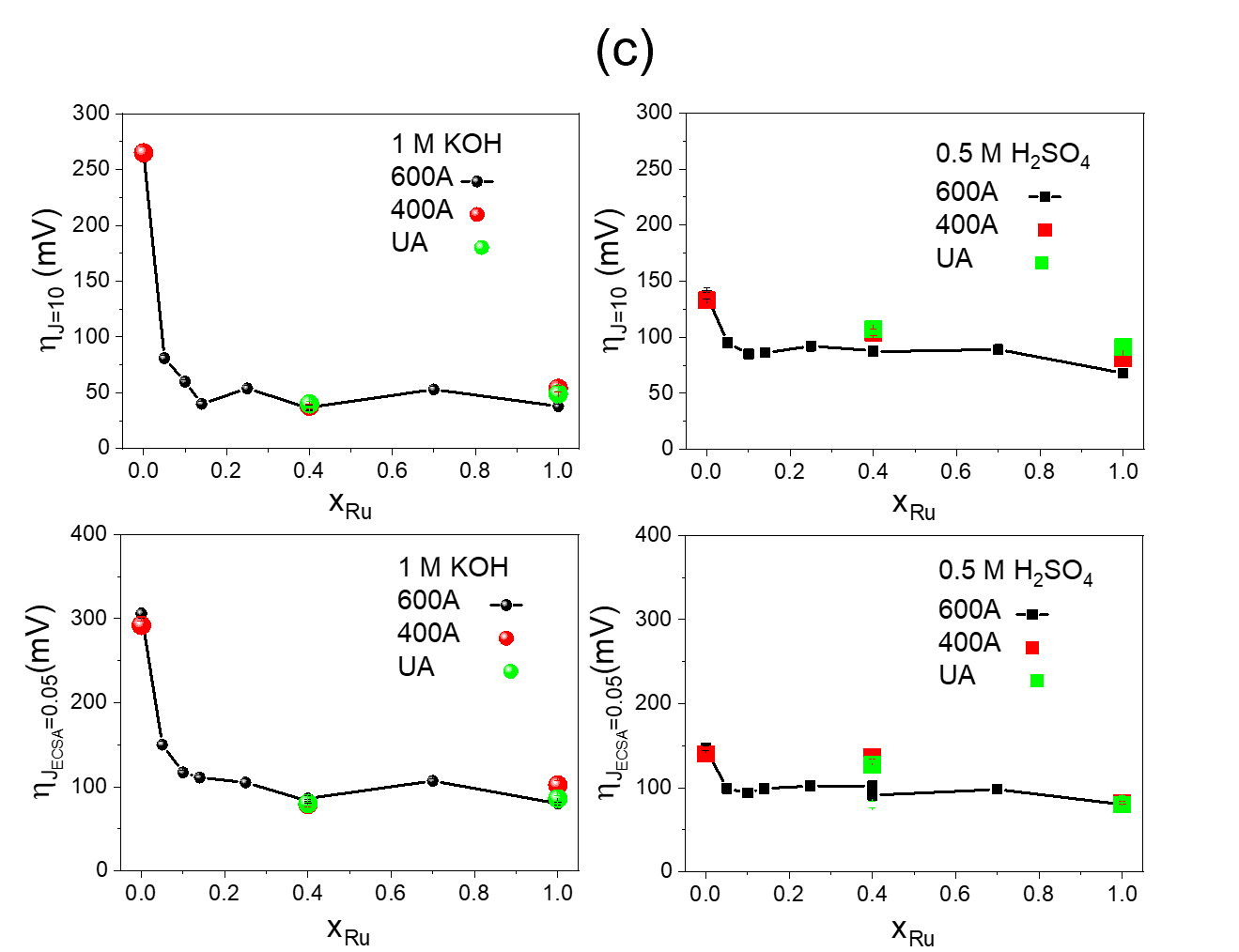


**Figure S10**. (a) LSV curves (scan rate: 2 mV s^–1^) *vs.* RHE for 400A and UA samples of **1**, **6**, and **9** toward HER in H_2_-saturated 1 M KOH. The values in parenthesis correspond to η*_J_*_=10_. Tafel plots (η *vs*. log *J*) derived from the LSV curves, based on the equation η = *b* log(*J*/*J*_0_), where *b* is the Tafel slope, and *J*_0_ is the exchange current density (extrapolated value at η = 0). Linear fit provides the *b* values (in parenthesis). (b) LSV curves and Tafel plots for the samples in. 0.5 M H_2_SO_4_. ECSA-normalized LSV. The corresponding η_J=10_, *b*, and η_JECSA=0.1_ values are listed in the parenthesis. (c) η*_J_*_=10_ and η*_J_*_ECSA=0.05_ *vs. x*_Ru_, showing a comparison with the values of 600A samples. The error bar represents ±SD obtained from an average of measurements of at least 3 samples. The quaternary alloy sample (sample **6**) exhibited enhanced HER activity relative to the ternary alloy one (sample **1**). In average, annealing at higher temperature (600 ℃) improves the HER performance.


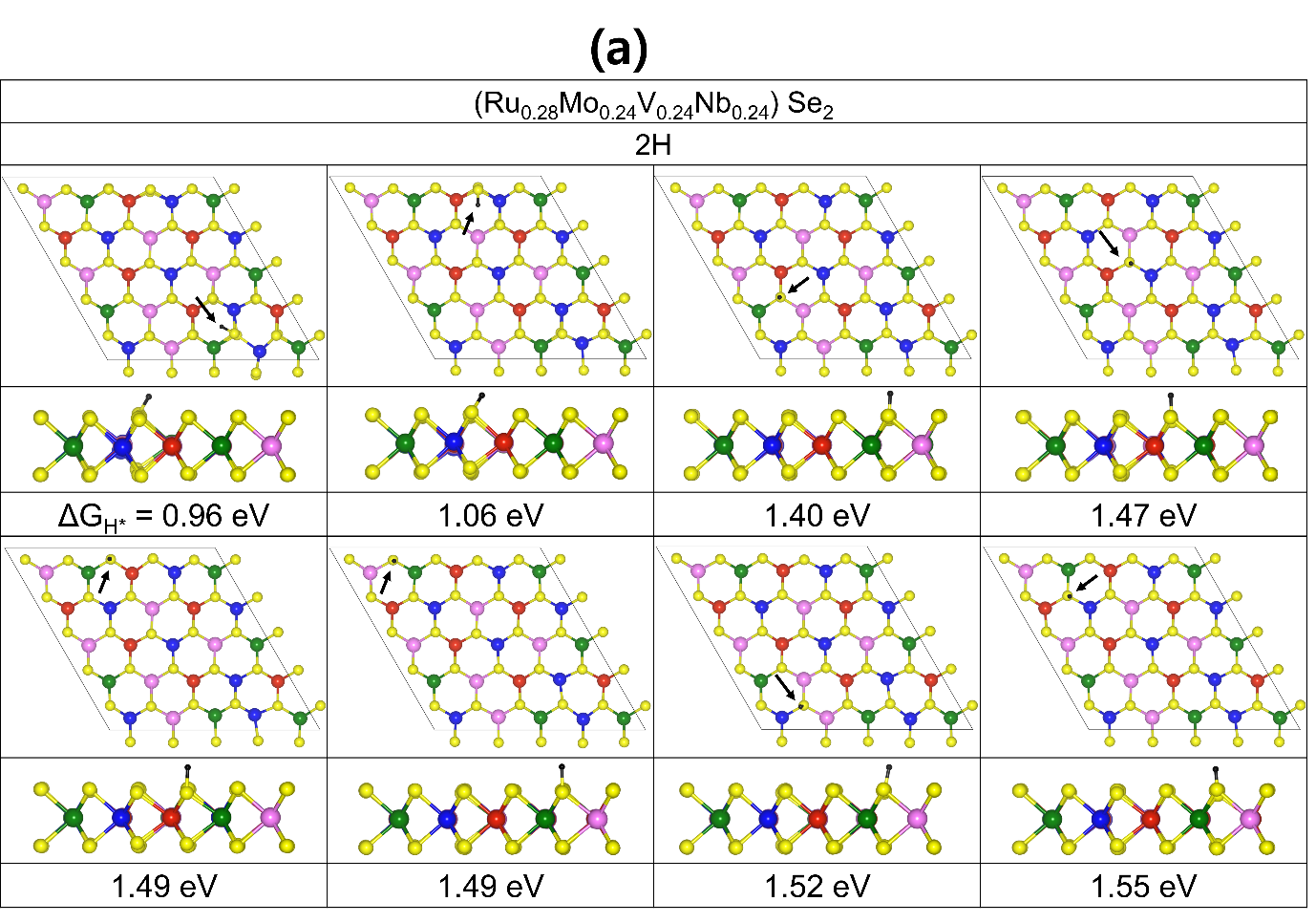


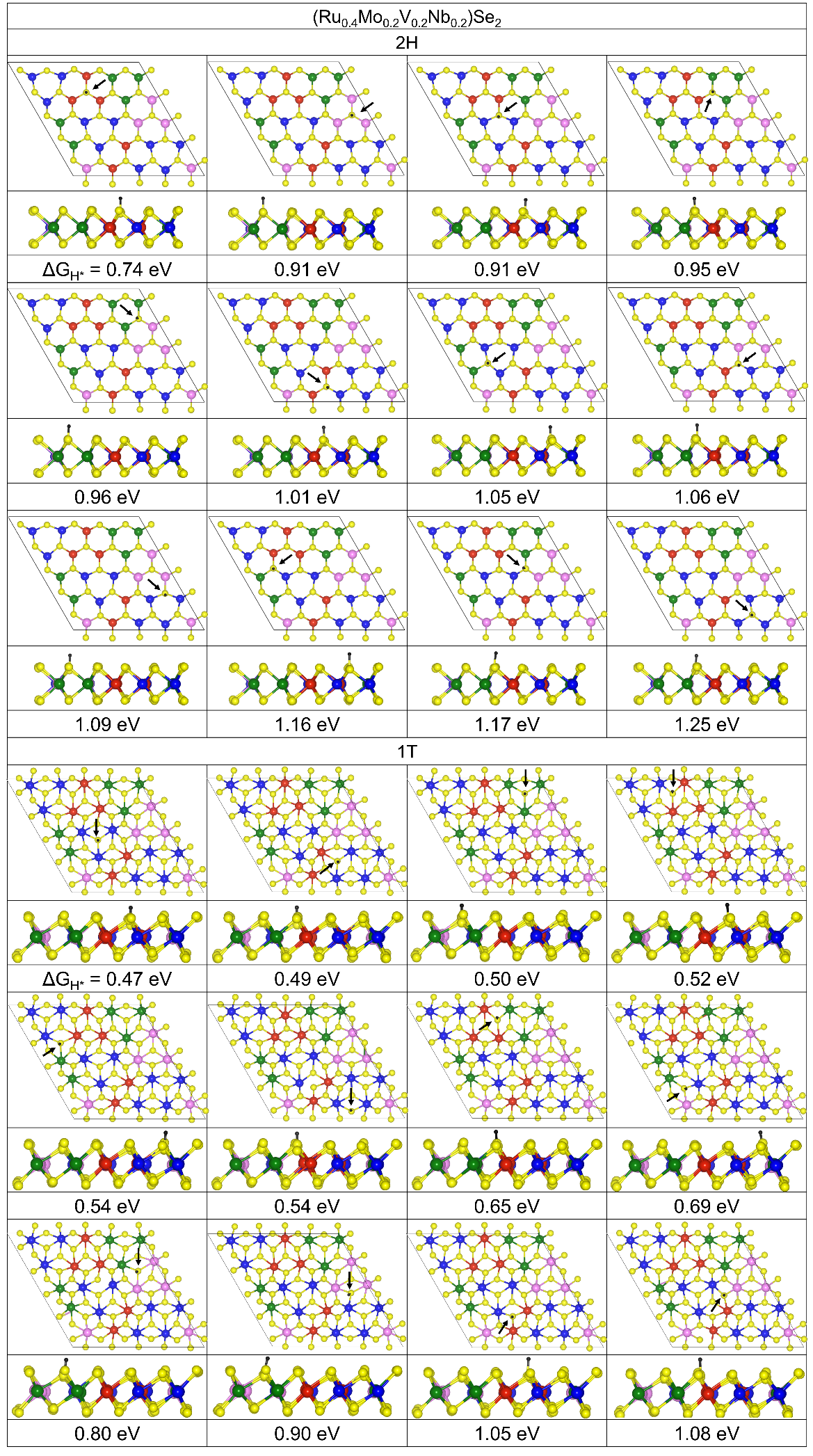


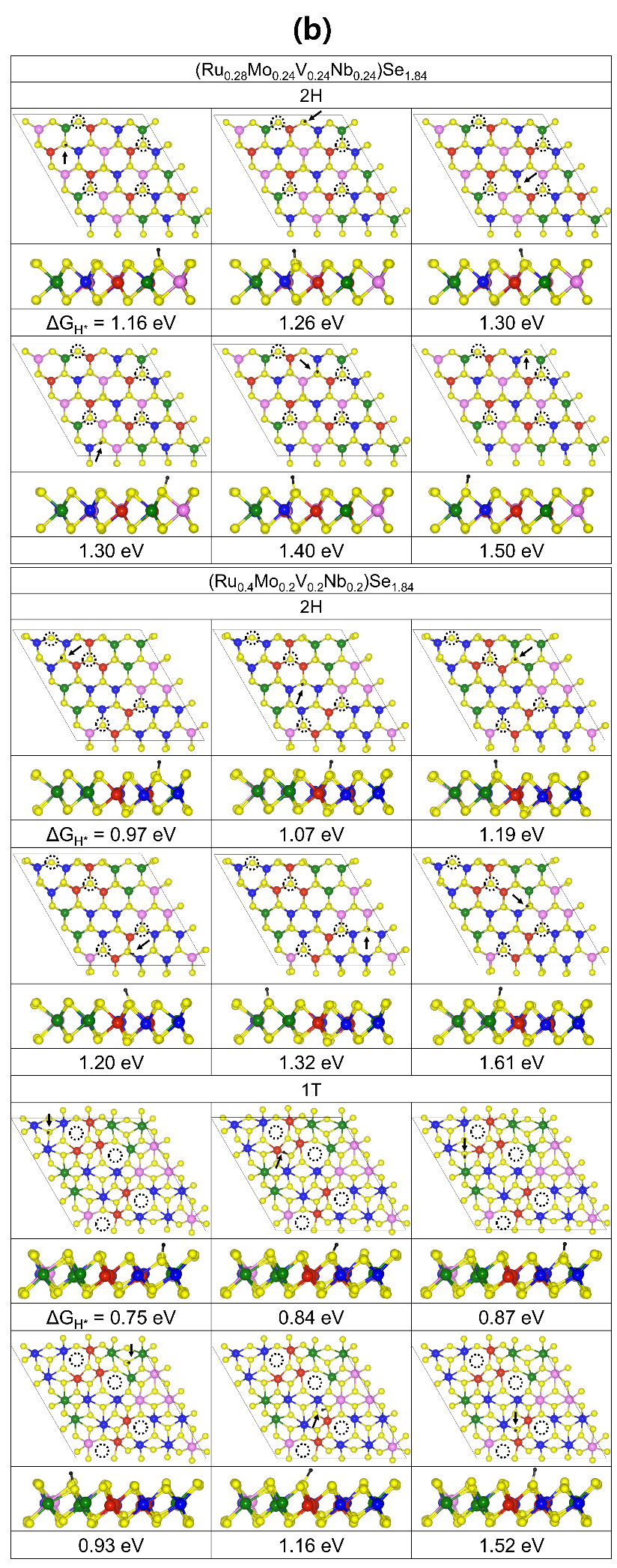


**
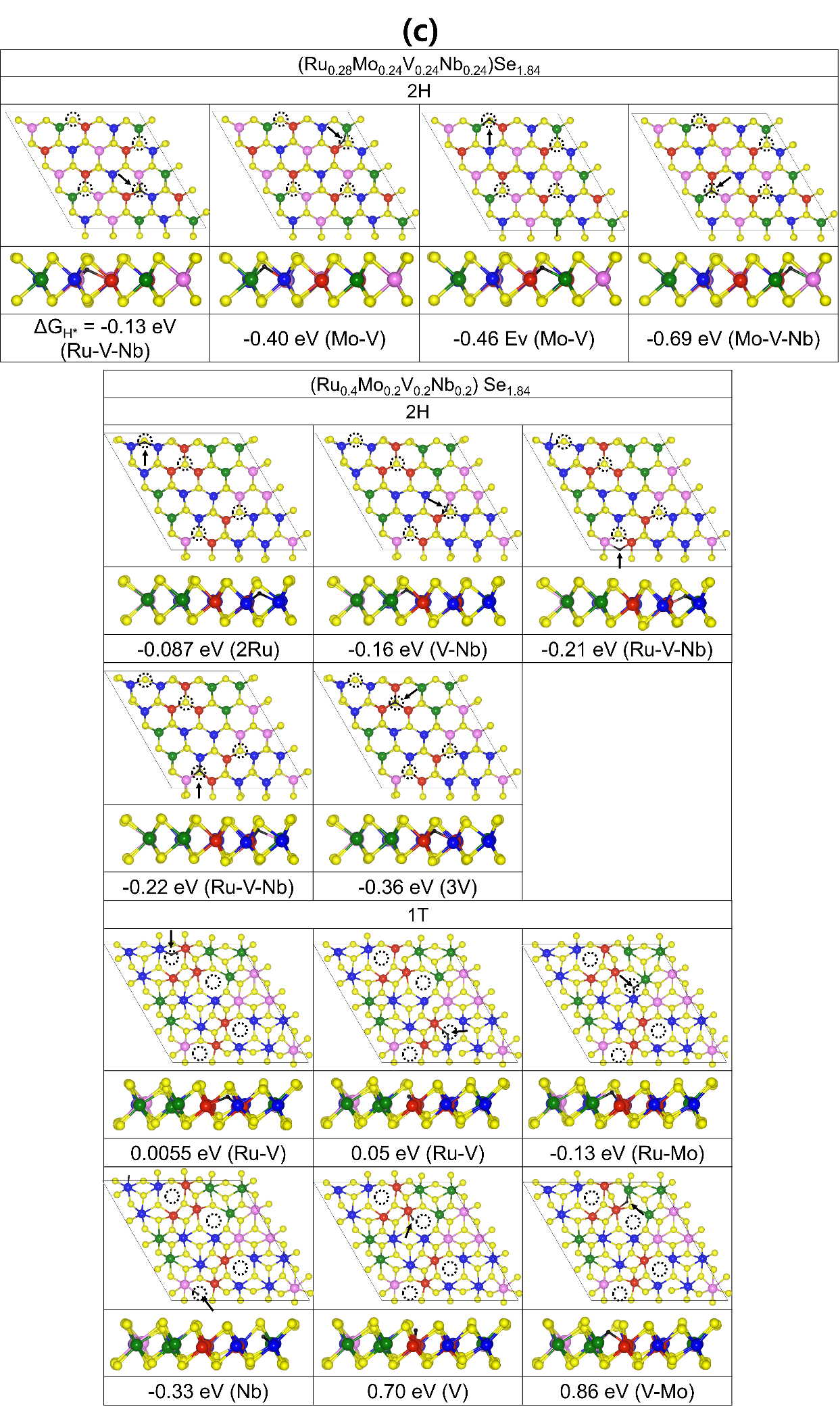
**

**Figure S11**. Crystal structures of the HER intermediates for (a) Se sites of 2H phase (Ru_0.28_Mo_0.24_V_0.24_Nb_0.24_)Se_2_ (*x*_Ru_ = 0.28) and 2H/1T phase (Ru_0.4_Mo_0.2_V_0.2_Nb_0.2_)Se_2_ (*x*_Ru_ = 0.4). (b) Se and (c) metal sites of Se vacancy models with 2H phase (Ru_0.28_Mo_0.24_V_0.24_Nb_0.24_)Se_1.84_ (*x*_Ru_ = 0.28) and 2H/1T phase (Ru_0.4_Mo_0.2_V_0.2_Nb_0.2_)Se_1.84_ (*x*_Ru_ = 0.4). Blue, pink, red, green, and yellow balls represent Ru, Mo, V, Nb, and Se atoms, respectively. Gibbs free energy for H adsorption (ΔG_H*_) is given for each site. Black balls represent H atoms. The Se vacancies are marked by dotted circles.

**References**

1. L. Li, Z. Qin, L. Ries, S. Hong, T. Michel, J. Yang, C. Salameh, M. Bechelany, P. Miele, M. Kaplan, M. Chhowalla, D. Voiry, *ACS Nano*, **2019**, *13*, 6824−6834.
2. G. Kresse, J. Furthmüller, *Phys. Rev.* *B*, **1996**, *54*, 11169−11186.
3. G. Kresse, J. Furthmüller, *Comput. Mater. Sci.*, **1996**, *6*, 15−50.
4. P. E. Blöchl, *Phys. Rev. B*, **1994**, *50*, 17953−17979.
5. S. Grimme, *J. Comput. Chem*., **2006**, *27*, 1787−1799.
6. S. Grimme, J. Antony, S. Ehrlich, H. A. Krieg, *J. Chem. Phys.*, **2010**, *132*, 154104.
7. J. Mou, Y. Gao, J. Wang, J. Ma, H. Ren, *RSC Advances*, **2019**, *9*, 11755−11761.
8. C. J. Cramer. *Essentials of Computational Chemistry: Theories and Models, 2^nd^ Edition*; John Wiley & Sons Ltd.: West Sussex, **2004**; Chapter 10.
9. P. Li, X. Duan, S. Wang, L. Zheng, Y. Li, H. Duan, Y. Kuang, X Sun, *Small,* **2019**, *15***,** 1904043.
10. T. L. Jin, X. Liu, H. Wang, X. Wu, Y Zhang, *Carbon*, **2020**, *162***,** 172−180.
11. K. Wang, B. Li, W. Wei, J. Wang, Q. Shen, P. Qu, *Nanoscale*, **2020**, *12***,** 23740−23747.
12. Y. Zhao, H. Cong, P. Li, D. Wu, S. Chen, W. Luo, *Angew. Chem. Int. Ed.*, **2021**, *60*, 7013−7017.
13. Z. Zhang, C. Jiang, P. Li, K. Yao, Z. Zhao, J. Fan, H. Li, H. Wang, *Small*, **2021**, *17*, 2007333.
14. D. Chen, R. Lu, Y. Yao, D. Wu, H. Zhao, R. Yu, Z. Pu, P. Wang, J. Zhu, J. Yu, P. Ji, Z. Kou, H. Tang, S. Mu, *J. Mater. Chem. A*, **2022**, *10*, 7637.
15. Z. Y. Fu, H. M. Xu, W. H. Li, G. P. Jin, S. K. Han, *Inorg. Chem.,* **2022**, *62*, 583−590.
16. D. Li, M. Zha, L. Feng, G. Hu, C. Hu, X. Wu, X. Wang, *Nanoscale*, 2022, **14,** 790−796.
17. W. Zhan, N. Li, S. Zuo, Z. Guo, C. Qiang, Z. Li, J. Ma, *CrystEngComm*, **2022**, *24*, 620–627.
18. N. Li, M. Shi, W. Zhan, W. Shen, M. Wu, G. Sun, Q. Li, J. Ma, *CrystEngComm*, **2023**, *25*, 981–987.
19. K. Wang, J. Zhou, M. Sun, F. Lin, B. Huang, F. Lv, L. Zeng, Q. Zhang, L. Gu, M. Luo, S. Guo, *Adv. Mater.*, **2023**, *35*, 2300980.
20. W. Fu, N. Li, M. Shi, M. Wu, G. Sun, W. Shen, Q. Li, J. Ma, *Langmuir*, **2023**, *39*, 13189−13196.
21. C. Cheng, W. Ao, H. Ren, Z. Shen, Z. Fan, T. Xu, W. Liu, Q. Zhang, P. Yin, L. Dai, *Appl. Catal. B: Environ*., **2023**, *331*, 122681.
22. N. Li, L. Huo, Q. Dong, B. Zhu, L. Huang, J. Ma, *Nanotech.*, **2024**, *35*, 115602.
23. Y. Sun, H. He, D. Zheng, F. Wang, F. Meng, W. Xu, G. Huang, J. Zhao, D. Mo, M. C Wiliiams, Q. Fang, *Int. J. Hydrogen Energy*, **2024**, 49, 955–963.
24. T. Zhu, J. Han, T. Sun, J. Zhao, X. Pi, J. Xu, K. Chen, *ACS Catal*., **2024**, *14*, 1914−1921.
25. Y. Chen, Y. Liu, L. Li, T. Sakthive, Z. Guo, Z Dai, *Adv. Funct. Mater*., **2024**, *34*, 2406587.
26. M. Zhu, H. Yu, C. Yang, Q. Deng, H. Liu, J. Huang, Y. Zhang, *Appl. Surf. Sci*., **2024**, *659*, 159916.
27. J. Li, A. W. Lashkova, M. Deconinck, M. Göbel, Y. Vaynzof, V. Lesnyak, A. Eychmüller, *ACS Appl. Mater. Interfaces*, **2024**, *16*, 36315−36321.
28. F. Sun, W. Bao, C. Feng, J. Bi, C. Yue, C. Zhang, N. Liu, H. Hao, Y. Lu, *Fuel*, **2024**, *370*, 131815.
29. J. Li, R. Miró, A. Wrzesińska-Lashkova, J. Yu, J. Arbiol, Y. Vaynzof, A. Shavel, V Lesnyak, *Adv. Funct. Mater.*, **2024**, *34*, 2404565.
30. Y. Ding, J. Zhu, M. Jiang, X. Zhan, J. Qin, X. Jiang, S. Wang, T. Meng, M. Cao, *J. Mater. Chem. A*, **2025**, *13*, 8456–8465.
31. I. Taguchi, H. P. Vaterlausf, R. Bichsel, F. Levy, H. Berger, M. Yumoto, *J. Phys. C: Solid State Phys.*, **1987**, *20*, 4241−4250.
32. C. R. Huang, M. C. Lee, Y. S. Huang, S. S. Lin, T. E. Dann, F. Z. Chien, *J. Phys. Chem. Solids*, **1990**, *51*, 387−390.
33. I. S. Kwon, S. J. Lee, J. Y. Kim, I. H. Kwak, S. J. Yoo, J.-G. Kim, J. Park, H. S. Kang, *ACS Nano*, **2023**, *17*, 2968−2979.
34. I. S. Kwon, I. H. Kwak, G. M. Zewdie, S. J. Lee, J. Y. Kim, S. J. Yoo, J.-G. Kim, J. Park, H. S. Kang, *ACS Nano*, **2022**, *16*, 12569−12579
35. I. H. Kwak, I. S. Kwon, J. Y. Kim, G. M. Zewdie, S. J. Lee, S. J. Yoo, J.-G. Kim, J. Park, H. S. Kang, *ACS Nano*, **2022**, *16*, 13949−13958.
